# Enantioselective addition of diphenyl phosphonate to ketimines derived from isatins catalyzed by binaphthyl-modified organocatalysts

**DOI:** 10.3762/bjoc.12.149

**Published:** 2016-07-20

**Authors:** Hee Seung Jang, Yubin Kim, Dae Young Kim

**Affiliations:** 1 Department of Chemistry, Soonchunhyang University, Soonchunhyang-Ro 22, Asan, Chungnam 31538, Korea

**Keywords:** 3-amino-3-phosphonyl-substituted oxindole, α-aminophosphonates, bifunctional organocatalyst, ketimines, organocatalysis, squaramide

## Abstract

Chiral binaphthyl-modified squaramide-catalyzed enantioselective addition of diphenyl phosphonate to ketimines derived from isatins has been achieved. This method affords practical and efficient access to chiral 3-amino-3-phosphonyl-substituted oxindole derivatives in high yields with excellent enantioselectivities (up to 99% ee).

## Introduction

α-Aminophosphonate derivatives are important compounds as structural mimics of natural α-amino acids [1–3]. Chiral α-aminophosphonates have been shown a wide range of biological activities including antibacterial [4] and anticancer properties [5], enzyme inhibition [6], peptide mimetic function [7], and herbicidal properties [8]. Since the biological activity of α-aminophosphonate derivatives is dependent upon the chirality of the α-position to the phosphorus atom, asymmetric synthesis of α-aminophosphonates has received considerable attention, and numerous catalytic enantioselective methods using chiral catalysts have been reported [9–13].

Oxindole and its derivatives can be exploited as important synthons to synthesize various alkaloid natural products and biologically active compounds [14–16]. In particular, 3,3-disubstituted oxindoles bearing a quaternary stereogenic center at the C3-position have been reported to be biologically active against a variety of targets [17–19]. Consequently, the asymmetric synthesis of 3,3-disubstituted oxindole derivatives has received significant research attention over the past few decades [20–22]. General approaches for the synthesis of chiral 3-substituted-3-aminooxindole derivatives include the amination of various 3-monosubstituted oxindoles [23–27] and the nucleophilic addition to ketimines derived from isatin derivatives [28–35]. Recently, there were a few reports on the synthesis of chiral 3-amino-3-phosphonyl-substituted oxindole derivatives by the catalytic enantioselective hydrophosphonation of ketimines [36–37]. The previous synthetic procedures suffered from several drawbacks, such as a high catalyst loading, long reaction time, and low temperature required for good enantioselectivity. Thus, new approaches for the organocatalytic enantioselective addition of diphenyl phosphonate to isatin imines are highly desired.

**Figure 1:**
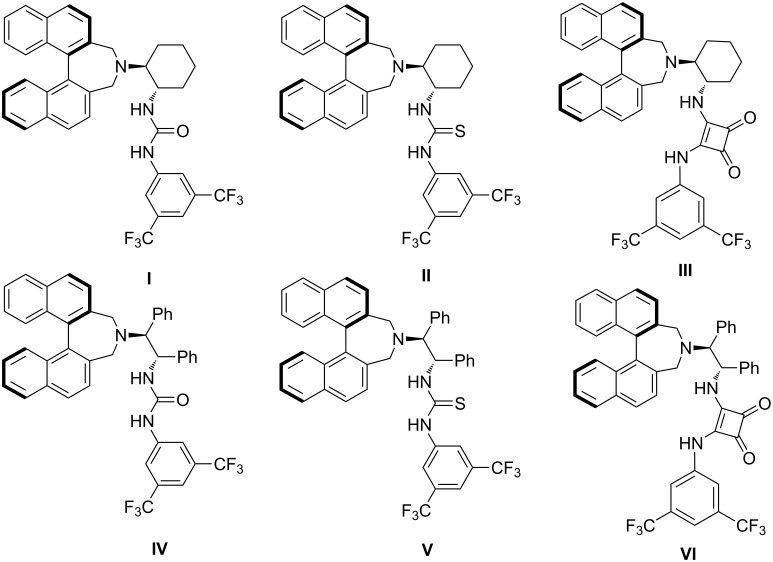
Structure of chiral bifunctional organocatalysts.

In connection with our ongoing research program on the design and application in asymmetric catalysis of organocatalysts [38–45], we have reported the catalytic asymmetric decarboxylative aldol addition reaction of isatins with benzoylacetic acids catalyzed by chiral binaphthyl-based squaramide [46]. Here we wish to report the enantioselective addition reaction of diphenyl phosphonate to ketimines derived from isatins catalyzed by binaphthyl-modified bifunctional organocatalysts (Figure 1).

## Results and Discussion

To determine suitable reaction conditions for the organocatalytic enantioselective addition reaction of diphenyl phosphonate to ketimines derived from isatins, we initially investigated a reaction system with ketimine **1a** derived from *N*-allylisatin and diphenyl phosphonate (**2**) with organocatalyst in the presence of 4 Å molecular sieves. We first surveyed the effect of the structure of bifunctional organocatalysts **I**–**VI** (Figure 1) on enantioselectivity in ethyl acetate at room temperature (Table 1, entries 1–6). Catalyst **III**, which is a binaphthyl-modified squaramide bifunctional organocatalyst, was the best catalyst for this enantioselective addition reaction (90% ee, Table 1, entry 3). In order to improve the selectivity, different solvents were tested in the presence of 10 mol % of catalyst **III** together with ketimine **1a** and diphenyl phosphonate (**2**). We obtained excellent results in ethyl acetate (85% yield, 90% ee, Table 1, entry 3), while a slight decrease in enationselectivities was observed when dichloromethane, chloroform, tetrahydrofuran, toluene, and methanol were used as the solvent (Table 1, entries 7–11). Under low catalyst loading of 2.5 mol %, this enantioselective addition reaction proceeded successfully to give **3a** without compromising the reactivity and enantioselectivity (Table 1, entries 3 and 12–14). Finally, lowering the reaction temperature to 0 °C with catalyst **III** improved the enantioselectivity (93% ee, Table 1, entry 15). Performing the reaction without 4 Å molecular sieves generated a lower yield (Table 1, entry 16).

**Table 1:** Optimization of the reaction conditions. ^a^

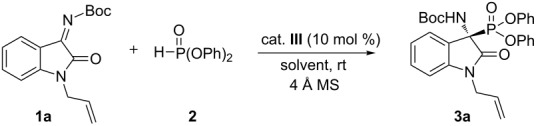					

entry	cat.	solvent	time (h)	yield^b^ (%)	ee^c^ (%)

1	**I**	EtOAc	9	**3a**, 85	73
2	**II**	EtOAc	11	**3a**, 94	62
3	**III**	EtOAc	9	**3a**, 85	90
4	**IV**	EtOAc	12	**3a**, 85	54
5	**V**	EtOAc	12	**3a**, 85	78
6	**VI**	EtOAc	9	**3a**, 95	74
7	**III**	CH_2_Cl_2_	3	**3a**, 92	87
8	**III**	CHCl_3_	7	**3a**, 82	80
9	**III**	THF	3	**3a**, 88	85
10	**III**	PhMe	6	**3a**, 75	87
11	**III**	MeOH	8	**3a**, 54	84
12^d^	**III**	EtOAc	16	**3a**, 82	90
13^e^	**III**	EtOAc	19	**3a**, 80	90
14^f^	**III**	EtOAc	25	**3a**, 76	81
15^e,g^	**III**	EtOAc	21	**3a**, 80	93
16^e,h^	**III**	EtOAc	21	**3a**, 58	93

^a^Reaction conditions: ketimine (**1a**, 0.3 mmol), diphenyl phosphonate (**2**, 0.45 mmol), catalyst (0.03 mmol), solvent (3.0 mL) in the presence of 150 mg molecular sieves. ^b^Isolated yield. ^c^Enantiopurity was determined by HPLC analysis using Chiralpak IB column. ^d^5 mol % catalyst loading. ^e^2.5 mol % catalyst loading. ^f^1.3 mol % catalyst loading. ^g^Reaction was performed at 0 °C. ^h^Reaction was performed without 4 Å molecular sieves.

With the optimized conditions in hand, we proceeded to investigate the scope of the enantioselective addition of diphenyl phosphonate (**2**) with various ketimines **1** in the presence of 2.5 mol % of binaphthyl-modified squaramide-tertiary amine catalyst **III** in ethyl acetate at 0 °C (Table 2). The corresponding addition products **3a**–**l** were formed in high yields (74–94%) with excellent enantioselectivities (up to 99% ee). The reaction of diphenyl phosphonate (**2**) with *N*-allylated and 5-halo-*N*-allylated isatin imines provided adducts **3a**–**d** in good yields (80–94%) with excellent enantioselectivities (93–97% ee, Table 2, entry 1–4). The addition of diphenyl phosphonate (**2**) to 5-chloro-*N*-substituted isatin imines **1e** and **1f** provided 3-amino-3-phosphonyl-substituted oxindole derivatives **3e** and **3f** in high yields (84% and 70%) with good enantioselectivities (99% ee and 88% ee, Table 2, entries 5 and 6). *N*-Benzylisatin imine **1g** and 5-halogen-*N*-benzylisatin imines **1h**–**j** reacted well with diphenyl phosphonate (**2**), giving 3-amino-3-phosphonyl-substituted oxindole derivatives **3g**–**j** in high yields (78–88%) with excellent enantioselectivities (98–99% ee) (Table 2, entries 7–10). Ketimine **1k** containing an electron donating group gave the desired product **3k** in high yield (79%) with excellent enantioselectivity (99% ee, Table 2, entry 11). The nucleophilic addition of diphenyl phosphonate (**2**) to ketimine **2l** derived from *N*-unprotected isatin was also studied. The adduct **3l** was isolated in 74% yield with 73% ee (Table 2, entry 12). Unfortunately, the reaction of diphenyl phosphonate (**2**) with *N*-Boc-ketimine **2m** provided adduct **3m** with low yield and enantioselectivity (Table 2, entry 13). The absolute configuration of adducts **3** was determined to be *R* by comparison of the specific rotations and HPLC properties with literature values [36.37].

**Table 2:** Substrate scope.^a^

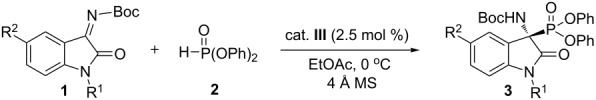				

entry	**1** (R^1^, R^2^)	time (h)	yield (%)^b^	ee (%)^c^

1	**1a** (R^1^ = CH_2_CH=CH_2_, R^2^ = H)	21	**3a**, 80	93
2	**1b** (R^1^ = CH_2_CH=CH_2_, R^2^ = F)	15	**3b**, 94	94
3	**1c** (R^1^ = CH_2_CH=CH_2_, R^2^ = Cl)	12	**3c**, 90	94
4	**1d** (R^1^ = CH_2_CH=CH_2_, R^2^ = Br)	19	**3d**, 84	97
5	**1e** (R^1^ = CH_2_C(CH_3_)=CH_2_, R^2^ = Cl)	48	**3e**, 84	99
6	**1f** (R^1^ = CH_2_CH=CHCH_3_, R^2^ = Cl)	47	**3f**, 70	88
7	**1g** (R^1^ = CH_2_C_6_H_5_, R^2^ = H)	21	**3g**, 87	99
8	**1h** (R^1^ = CH_2_C_6_H_5_, R^2^ = F)	20	**3h**, 88	99
9	**1i** (R^1^ = CH_2_C_6_H_5_, R^2^ = Cl)	16	**3i**, 78	98
10	**1j** (R^1^ = CH_2_C_6_H_5_, R^2^ = Br)	32	**3j**, 84	99
11	**1k** (R^1^ = CH_2_C_6_H_5_, R^2^ = OMe)	48	**3k**, 79	99
12	**1l** (R^1^ = H, R^2^ = Cl)	31	**3l**, 74	73
13	**1m** (R^1^ = Boc, R^2^ = H)	48	**3m**, 45	26

^a^Reaction conditions: ketimines (**1**, 0.3 mmol), diphenyl phosphonate (**2**, 0.45 mmol), catalyst (**III**, 7.5 μmol), EtOAc (3.0 mL) at 0 °C in the presence of 150 mg molecular sieve. ^b^Isolated yield. ^c^Enantiopurity was determined by HPLC analysis using Chiralpak IA (for **3f**), IB (for **3a**), IC (for **3b**–**e**, **3g**–**j**), and AD-H (for **3k**, **3l**) columns.

**Figure 2:**
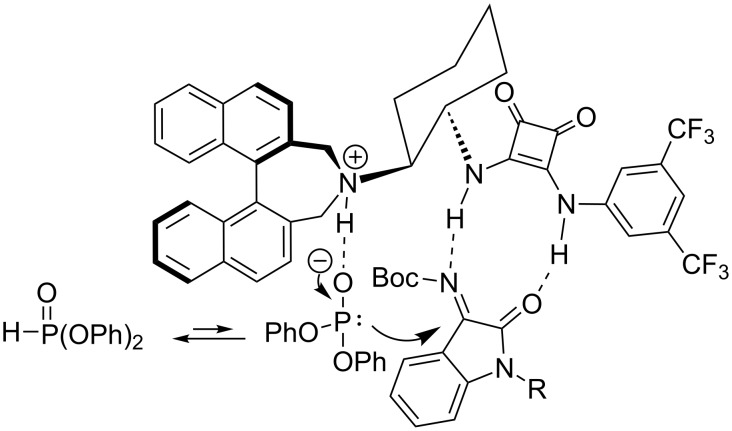
Proposed stereochemical model.

The stereochemical outcome in the above addition reaction was rationalized by a proposed stereochemical model. We propose that ketimine **1** is activated by the squaramide moiety through hydrogen bonding, and diphenyl phosphonate (**2**) is activated by the basic nitrogen atom in the tertiary amine of catalyst **III**. Then, diphenyl phosphonate (**2**) attacks the *re*-face of the carbon in ketimine **1** as shown in Figure 2.

**Scheme 1:**
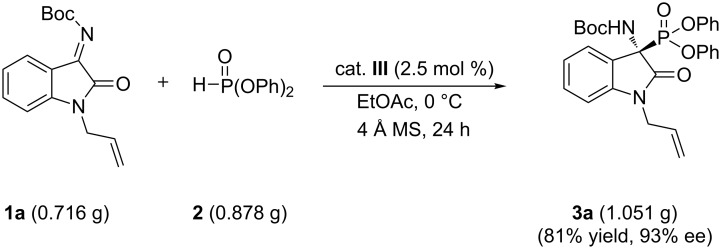
Gram scale addition of ketimine **1a** and diphenyl phosphonate (**2**).

To further demonstrate the synthetic potential of this method, we performed the addition reaction at the gram scale. As shown in Scheme 1, when ketimine **1a** was treated with diphenyl phosphonate (**2**) in the presence of 2.5 mol % of catalyst **III** at 0 °C, the desired product **3a** was obtained in 81% yield and 93% ee (Scheme 1).

## Conclusion

In conclusion, we have developed a practical and efficient catalytic enantioselective addition reaction of diphenyl phosphonate (**2**) with various ketimines **1** derived from isatins. This transformation is catalyzed by binaphthyl-modified squaramide catalyst **III** with low catalyst loading (2.5 mol %). Chiral 3-amino-3-phosphonyl-substituted oxindole derivatives were obtained in high yields and excellent enantioselectivities were observed (up to 99% ee). This reaction affords valuable and easy access to chiral 3-amino-3-phosphonyl-substituted oxindole derivatives.

## Experimental

**General procedure for the enantioselective addition of diphenyl phosphonate (2) to ketimines derived from isatins 1**: To a solution of ketimine **1** (0.3 mmol), diphenyl phosphonate (**2**, 0.45 mmol), and 4 Å molecular sieves (150 mg) in ethyl acetate (3 mL), the catalyst (**III**, 7.5 μmol) was added at 0 °C. The reaction mixture was stirred for 12–48 h. After completion of the reaction, the resulting solution was concentrated in vacuo and the obtained residue was purified by flash chromatography (EtOAc–hexane) to afford the corresponding adducts **3**.

## Supporting Information

File 1 Experimental and analytical data.
