# Supplementary material for: Enantioselective addition of diphenyl phosphonate to ketimines derived from isatins catalyzed by binaphthyl-modified organocatalysts
Source: Beilstein J Org Chem. 2016 Jul 20;12:1551–6. doi: 10.3762/bjoc.12.149 (PMC4979900; doi:10.3762/bjoc.12.149)
Supplement: File 1 — Experimental and analytical data. [file Beilstein_J_Org_Chem-12-1551-s001.pdf]

Supporting Information

for

Enantioselective addition of diphenyl phosphonate to ketimines derived from isatins catalyzed by binaphthyl-modified organocatalysts

Hee Seung Jang, Yubin Kim and Dae Young Kim*

Address: Department of Chemistry, Soonchunhyang University, Soonchunhyang-Ro 22, Asan, Chungnam 31538, Korea

Email: Dae Young Kim* - dyoung@sch.ac.kr

*Corresponding author

Experimental and analytical data

1. General

All commercial reagents and solvents were used without purification. TLC analyses were carried out on pre-coated silica gel plates with F_{254} indicator. Visualization was accomplished by UV light (254 nm), I_2, *p*-anisaldehyde, ninhydrin, and phosphomolybdic acid solution as an indicator. Purification of reaction products was carried out by flash chromatography using E. Merck silica gel 60 (230–400 mesh). ^{1}H NMR and ^{13}C NMR spectra were recorded on a Jeol 400 MHz NMR (400 MHz for ^{1}H, 100 MHz for ^{13}C). Chemical shift values (δ) are reported in ppm relative to Me_4Si (δ 0.0 ppm). Optical rotations were measured on a JASCO-DIP-1000 digital polarimeter with a sodium lamp. The enantiomeric excesses (ees) were determined by HPLC. HPLC analyses were performed on Younglin M930 Series and Younglin M9100 Series machines and were measured at 254 nm using the indicated chiral column.

2. General procedure for the enantioselective addition of diphenyl phosphonate (2) to ketimines derived from isatins 1.

To a solution of ketimine **1** (0.3 mmol), diphenyl phosphonate (**2**, 0.45 mmol), 4 Å MS (150 mg) in ethyl acetate (3 mL), the catalyst (**III**, 7.5 μmol) was added at 0 °C. The reaction mixture was stirred for 12–48 h. After completion of the reaction, the resulting solution was concentrated in vacuo and the obtained residue was purified by flash chromatography (EtOAc–hexane) to afford the corresponding adducts **3**. Products **3** are known compounds, and their data were identical to those reported in the literature [1,2].

3. Characterization data of products 3.

(*R*)-*tert*-Butyl (1-allyl-3-(diphenoxyphosphoryl)-2-oxoindolin-3-yl)carbamate (3a)

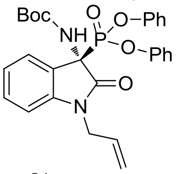

[α]$^{24}_{D}$ = -15.92 (c = 1, $CHCl_3$); ^{1}H NMR ($CDCl_3$, 400 MHz) δ: 1.29 (s, 9H), 4.24 (d, *J* = 13.2 Hz, 1H), 4.64 (d, *J* = 15.6 Hz, 1H), 5.18 (d, *J* = 10.4 Hz, 1H), 5.38 (d, *J* = 17.6 Hz, 1H), 5.80–5.91 (m, 2H), 6.69 (d, *J* = 8.9 Hz, 2H), 6.867(d, *J* = 8.0 Hz, 1H), 7.07–7.26 (m, 7H), 7.31–7.40 (m, 3H), 7.53 (dd, *J* = 1.4 and 7.4 Hz, 1H); ^{13}C NMR ($CDCl_3$, 100 MHz) δ: 28.0, 43.0, 81.3, 109.3, 117.7, 120.2 (d, *J* = 3.8 Hz), 120.9 (d, *J* = 3.8 Hz), 122.9 (d, *J* = 3.8 Hz), 125.2 (d, *J* = 3.9 Hz), 125.6, 125.8, 129.6, 129.8, 130.0 (d, *J* = 3.1 Hz), 130.8, 143.6 (d, *J* = 7.7 Hz), 150.0 (dd, *J* = 10.6 and 19.3 Hz), 153.5 (d, *J* = 18.4 Hz), 170.7; ^{31}P NMR ($CDCl_3$, 162 MHz) δ: 7.25; HPLC (90 : 10, *n*-hexane : *i*-PrOH, 254nm, 1mL/min) Chiralpak IB column, t_R = 7.8 (major), t_R = 11.4 (minor), 93% ee.

(*R*)-*tert*-Butyl (1-allyl-3-(diphenoxyphosphoryl)-5-fluoro-2-oxoindolin-3-yl)carbamate (3b)

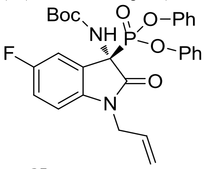

[α]$^{25}_{D}$ = -11.08 (c = 1, $CHCl_3$); ^{1}H NMR ($CDCl_3$, 400 MHz) δ: 1.32 (s, 9H), 4.24 (d, *J* = 14.8 Hz, 1H), 4.60 (d, *J* = 15.6 Hz, 1H), 5.19 (d, *J* = 10.8 Hz, 1H), 5.37 (d, *J* = 17.2 Hz, 1H), 5.78–5.90 (m, 2H), 6.78 (m, 3H), 6.91–7.40 (m, 8H), 7.28–7.36 (m, 3H); ^{13}C NMR ($CDCl_3$, 100 MHz) δ: 28.1, 43.2, 81.6, 110.0 (d, *J* = 6.8 Hz), 113.1 (d, *J* = 3.8 Hz), 113.3 (d, *J* = 4.8 Hz), 116.2 (d, *J* = 2.9 Hz), 116.5 (d, *J* = 2.8 Hz), 117.8, 120.1 (d, *J* = 3.8 Hz), 120.8 (d, *J* = 3.9 Hz), 125.7, 126.0, 129.7, 129.9, 130.6, 149.8 (d, *J* = 9.6 Hz), 153.5 (d, *J* = 19.3 Hz), 170.6; ^{31}P NMR ($CDCl_3$, 162 MHz) δ: 6.57; HPLC (70 : 30, *n*-hexane : *i*-PrOH, 254nm, 1mL/min) Chiralpak IC column, t_R = 5.6 (minor), t_R = 7.1 (major), 94% ee.

(*R*)-*tert*-Butyl (1-allyl-5-chloro-3-(diphenoxyphosphoryl)-2-oxoindolin-3-yl)carbamate (3c)

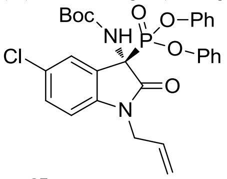

[α]$^{27}_{D}$ = 5.64 (c = 1, $CHCl_3$); ^{1}H NMR ($CDCl_3$, 400 MHz) δ: 1.32 (s, 9H), 4.26 (s, 1H), 4.59 (d, J = 15.6 Hz, 1H), 5.19 (d, J = 10.8 Hz, 1H), 5.37 (d, J = 17.2 Hz, 1H), 5.78–5.90 (m, 2H), 6.80 (t, J = 8.6 Hz, 2H), 7.12–7.23 (m, 6H), 7.29–7.43 (m, 3H), 7.50 (s, 1H); ^{13}C NMR ($CDCl_3$, 100 MHz) δ: 28.1, 43.2, 81.7, 110.4, 117.9, 120.1 (d, *J* = 4.8 Hz), 120.7 (d, *J* = 3.8 Hz), 125.4 (d, *J* = 3.8 Hz), 125.7, 125.9, 128.3 (d, *J* = 3.8 Hz), 129.7, 129.9, 130.5, 142.2 (d, *J* = 6.7 Hz), 149.9 (dd, *J* = 10.1, 13.0 Hz), 153.4 (d, *J* = 18.3 Hz), 170.4; ^{31}P NMR ($CDCl_3$, 162 MHz) δ: 6.38; HPLC (70 : 30, *n*-hexane : *i*-PrOH, 254nm, 1mL/min) Chiralpak IC column, t_R = 6.5 (minor), t_R = 8.6 (major), 94% ee.

(*R*)-*tert*-Butyl (1-allyl-5-bromo-3-(diphenoxyphosphoryl)-2-oxoindolin-3-yl)carbamate (3d)

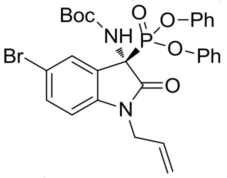

[α]$^{26}_{D}$ = 16.36 (c = 1, $CHCl_3$); ^{1}H NMR ($CDCl_3$, 400 MHz) δ: 1.32 (s, 9H), 4.24 (d, *J* = 15.2 Hz, 1H), 4.58 (d, *J* = 14.8 Hz, 1H), 5.19 (d, *J* = 11.2 Hz, 1H), 5.37 (d, *J* = 16.8 Hz, 1H), 5.77–5.90 (m, 2H), 6.74 (d, *J* = 8.4 Hz, 1H), 6.82 (d, *J* = 6.8 Hz, 2H), 7.11–7.24 (m, 6H), 7.32–7.51 (m, 3H), 7.63 (t, *J* = 2.4 Hz, 1H); ^{13}C NMR ($CDCl_3$, 100 MHz) δ: 28.1, 43.2, 81.7, 110.8, 115.4 (d, *J* = 3.8 Hz), 117.9, 120.1 (d, *J* = 3.8 Hz), 120.7 (d, *J* = 3.8 Hz), 125.6,

126.0, 128.1 (d, J = 3.8 Hz), 129.7, 129.9, 130.4, 132.8 (d, J = 2.8 Hz), 142.7 (d, J = 6.8 Hz), 149.9 (dd, J = 9.6, 13.5 Hz), 153.4 (d, J = 17.3 Hz), 170.3; ^{31}P NMR ($CDCl_3$, 162 MHz) δ: 6.36; HPLC (70 : 30, *n*-hexane : *i*-PrOH, 254nm, 1mL/min) Chiralpak IC column, t_R = 6.1 (minor), t_R = 7.8 (major), 97% ee.

(*R*)-*tert*-Butyl (5-chloro-3-(diphenoxyphosphoryl)-1-(2-methylallyl)-2-oxoindolin-3-yl)carbamate (3e)

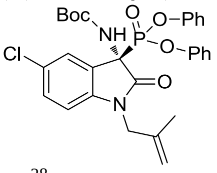

$[\alpha]^{28}_D$ = 9.84 (c = 1, $CHCl_3$); ^{1}H NMR ($CDCl_3$, 400 MHz) δ: 1.32 (s, 9H), 1.77(s, 3H), 4.14 (s, 1H), 4.53 (d, J = 13.2 Hz, 1H), 4.92 (s, 1H), 5.04 (s, 1H), 5.89(d, J = 2.9 Hz, 1H), 6.79–6.82 (m, 3H), 7.10-7.14 (m, 1H), 7.19–7.23 (m, 5H), 7.28–7.36 (m, 3H), 7.50 (t, J = 2.4 Hz, 1H); ^{13}C NMR ($CDCl_3$, 100 MHz) δ: 19.9, 28.1, 46.9, 81.7, 110.4, 113.2, 120.2 (d, J = 4.8 Hz), 120.8 (d, J = 3.8 Hz), 125.3 (d, J = 4.8 Hz), 125.8, 126.0, 128.2 (d, J = 3.8 Hz), 129.7, 129.9, 138.6, 142.5 (d, J = 6.8 Hz), 149.9 (t, J = 10.6 Hz), 153.4 (d, J = 17.4 Hz), 170.6; ^{31}P NMR ($CDCl_3$, 162 MHz) δ: 6.54; HPLC (70 : 30, *n*-hexane : *i*-PrOH, 254nm, 1mL/min) Chiralpak IC column, t_R = 7.0 (major), t_R = 10.0 (minor), >99% ee.

(*R*)-*tert*-Butyl (1-(*trans*-but-2-en-1-yl)-5-chloro-3-(diphenoxyphosphoryl)-2-oxoindolin-3-yl)carbamate (3f)

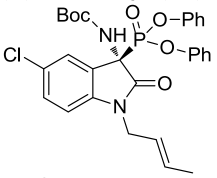

$[\alpha]^{26}_D$ = 12.24 (c = 1, $CHCl_3$); ^{1}H NMR ($CDCl_3$, 400 MHz) δ: 1.32 (s, 9H), 1.61(s, 3H), 4.10-4.33 (m, 1H), 4.50-4.63 (m, 1H), 5.39-5.46 (m, 1H), 5.69-5.88 (m, 1H), 6.75-6.83 (m, 3H), 7.10-7.13 (m, 1H), 7.19–7.23 (m, 5H), 7.28–7.44 (m, 3H), 7.48-7.50 (m, 1H); ^{13}C NMR ($CDCl_3$, 100 MHz) δ: 17.6, 28.1, 42.2, 42.6, 53.4, 81.7, 110.1, 110.3, 120.1 (d, J = 4.8 Hz), 120.7 (d, J = 3.8 Hz), 123.5, 123.6, 125.4 (d, J = 3.8 Hz), 125.7, 125.9, 128.1, 129.0, 129.5, 129.7, 129.9, 141.5, 142.3 (d, J = 6.7 Hz), 149.9 (t, J = 11.6 Hz), 153.5 (d, J = 17.2 Hz), 170.3; ^{31}P NMR ($CDCl_3$, 162 MHz) δ: 6.38; HPLC (70 : 30, *n*-hexane : *i*-PrOH, 254nm, 1mL/min) Chiralpak IA column, t_R = 16.6, 19.4 (minor), t_R = 22.7, 35.15 (major), 88, 85% ee.

(*R*)-*tert*-Butyl (1-benzyl-3-(diphenoxyphosphoryl)-2-oxoindolin-3-yl)carbamate (3g)

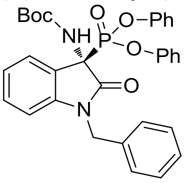

$[\alpha]^{25}_D$ = -3.32 (c = 1, $CHCl_3$); ^{1}H NMR ($CDCl_3$, 400 MHz) δ: 1.30 (s, 9H), 4.76 (d, J = 13.6 Hz, 1H), 5.30 (d, J = 15.6 Hz, 1H), 5.93 (d, J = 11.6 Hz, 1H), 6.61 (d, J = 7.6 Hz, 1H), 6.72 (d, J = 7.6 Hz, 1H), 7.03–7.24 (m, 10H), 7.30–7.40 (m, 5H), 7.54 (d, J = 7.2 Hz, 1H); ^{13}C NMR ($CDCl_3$, 100 MHz) δ: 28.0, 44.6, 81.4, 109.5, 120.1 (d, J = 3.8 Hz), 120.9 (d, J = 4.7 Hz), 122.9, 125.2 (d, J = 3.8 Hz), 125.5, 125.8, 127.2, 127.5, 129.6, 129.8, 130.0, 135.4, 143.6 (d, J = 4.7 Hz), 149.9 (dd, J = 10.6, 21.6 Hz), 153.8 (d, J = 18.3 Hz), 171.1; ^{31}P NMR ($CDCl_3$, 162 MHz) δ: 7.27; HPLC (70 : 30, *n*-hexane : *i*-PrOH, 254nm, 1mL/min) Chiralpak IC column, t_R = 8.0 (minor), t_R = 10.6 (major), >99% ee.

(*R*)-*tert*-Butyl (1-benzyl-3-(diphenoxyphosphoryl)-5-fluoro-2-oxoindolin-3-yl)carbamate (3h)

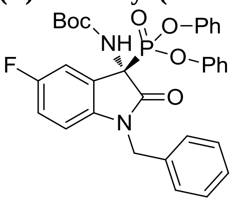

$[\alpha]^{25}_D$ = -5.40 (c = 1, $CHCl_3$); ^{1}H NMR ($CDCl_3$, 400 MHz) δ: 1.34 (s, 9H), 4.79 (d, J = 16.0 Hz, 1H), 5.25 (d, J = 15.2 Hz, 1H), 5.94 (d, J = 11.6 Hz, 1H), 6.62 (dd, J = 4.0 and 8.8 Hz, 1H), 6.71 (d, J = 8.4 Hz, 2H), 6.91–6.95 (m, 1H), 7.08-7.24 (m, 8H), 7.27–7.39 (m, 6H); ^{13}C NMR ($CDCl_3$, 100 MHz) δ: 28.1, 44.8, 81.7, 110.1 (d, J =

9.6 Hz), 113.1, 116.3, 116.5, 120.1 (d, *J* = 3.8 Hz), 120.8 (d, *J* = 4.7 Hz), 125.7, 126.0, 127.2, 127.6, 128.8, 128.9, 129.7, 130.0, 135.1, 149.9 (dd, *J* = 9.6, 13.0 Hz), 153.5 (d, *J* = 17.4 Hz), 171.0; ^{31}P NMR ($CDCl_3$, 162 MHz) δ: 6.63; HPLC (70 : 30, *n*-hexane : *i*-PrOH, 254nm, 1mL/min) Chiralpak IC column, t_R = 6.5 (minor), t_R = 8.2 (major), >99% ee.

(*R*)-*tert*-Butyl (1-benzyl-5-chloro-3-(diphenoxyphosphoryl)-2-oxoindolin-3-yl)carbamate (3i)

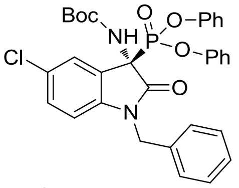

$[\alpha]^{26}_D$ = 17.48 (c = 1, $CHCl_3$); ^{1}H NMR ($CDCl_3$, 400 MHz) δ: 1.34 (s, 9H), 4.79 (d, *J* = 8.8 Hz, 1H), 5.23 (d, *J* = 15.2 Hz, 1H), 5.93 (d, *J* = 11.2 Hz, 1H), 6.62 (d, *J* = 8.8 Hz, 1H), 6.74 (d, *J* = 8.4 Hz, 2H), 7.17-7.25 (m, 10H), 7.31–7.38 (m, 4H), 7.50 (t, *J* = 2.2 Hz, 1H); ^{13}C NMR ($CDCl_3$, 100 MHz) δ: 28.1, 44.7, 81.8, 110.5, 120.1 (d, *J* = 4.8 Hz), 120.8 (d, *J* = 4.8 Hz), 125.4 (d, *J* = 3.9 Hz), 125.7, 126.0, 127.2, 127.7, 128.4 (d, *J* = 3.9 Hz), 128.8, 129.7, 130.0, 134.9, 142.1 (d, *J* = 6.8 Hz), 149.8 (dd, *J* = 9.7, 13.5 Hz), 153.5 (d, *J* = 17.4 Hz), 170.8; ^{31}P NMR ($CDCl_3$, 162 MHz) δ: 6.44; HPLC (70 : 30, *n*-hexane : *i*-PrOH, 254nm, 1mL/min) Chiralpak IC column, t_R = 5.8 (minor), t_R = 7.2 (major), 98% ee.

(*R*)-*tert*-Butyl (1-benzyl-5-bromo-3-(diphenoxyphosphoryl)-2-oxoindolin-3-yl)carbamate (3j)

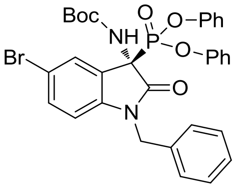

$[\alpha]^{26}_D$ = 34.28 (c = 1, $CHCl_3$); ^{1}H NMR ($CDCl_3$, 400 MHz) δ: 1.34 (s, 9H), 4.80 (s, 1H), 5.22 (d, *J* = 15.6 Hz, 1H), 5.94 (d, *J* = 11.2 Hz, 1H), 6.57 (d, *J* = 8.0 Hz, 1H), 6.74 (d, *J* = 8.4 Hz, 2H), 7.09-7.24 (m, 10H), 7.29–7.38 (m, 6H), 7.63 (t, *J* = 2.0 Hz, 1H); ^{13}C NMR ($CDCl_3$, 100 MHz) δ: 28.1, 44.7, 81.8, 111.0, 115.5 (d, *J* = 3.8 Hz), 120.1 (d, *J* = 3.8 Hz), 120.8 (d, *J* = 3.8 Hz), 125.7, 126.0, 127.2, 127.7, 128.1 (d, *J* = 4.8 Hz), 128.8, 129.7, 129.9, 132.8, 134.9, 142.6 (d, *J* = 7.8 Hz), 149.9 (dd, *J* = 10.6, 13.5 Hz), 153.5 (d, *J* = 18.3 Hz), 170.7; ^{31}P NMR ($CDCl_3$, 162 MHz) δ: 6.41; HPLC (70 : 30, *n*-hexane : *i*-PrOH, 254nm, 1mL/min) Chiralpak IC column, t_R = 6.5 (minor), t_R = 8.1 (major), >99% ee.

(*R*)-*tert*-Butyl (1-benzyl-3-(diphenoxyphosphoryl)-5-methoxy-2-oxoindolin-3-yl)carbamate (3k)

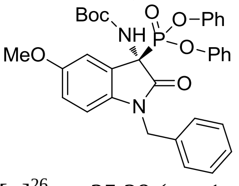

$[\alpha]^{26}_D$ = 35.28 (c = 1, $CHCl_3$); ^{1}H NMR ($CDCl_3$, 400 MHz) δ: 1.32 (s, 9H), 3.69 (s, 3H), 4.76 (s, 1H), 5.26 (d, *J* = 15.6 Hz, 1H), 5.93 (d, *J* = 12.0 Hz, 1H), 6.57-6.80 (m, 4H), 7.05–7.40 (m, 15H); ^{13}C NMR ($CDCl_3$, 100 MHz) δ: 28.1, 44.7, 55.8, 81.8, 110.0, 111.9, 115.0, 120.1 (d, *J* = 4.8 Hz), 120.9 (d, *J* = 3.8 Hz), 125.5, 125.8, 127.2, 127.4, 128.7, 129.5, 129.8, 135.4, 137.0 (d, *J* = 6.8 Hz), 150.0 (dd, *J* = 9.7, 18.3 Hz), 153.6 (d, *J* = 18.3 Hz), 156.1 (d, J = 3.9 Hz), 170.8; ^{31}P NMR ($CDCl_3$, 162 MHz) δ: 7.19; HPLC (70 : 30, *n*-hexane : *i*-PrOH, 254nm, 1mL/min) Chiralpak AD-H column, t_R = 17.0 (minor), t_R = 24.9 (major), >99% ee.

(*R*)-*tert*-Butyl (5-chloro-3-(diphenoxyphosphoryl)-2-oxoindolin-3-yl)carbamate (3l)

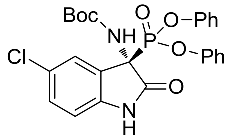

$[\alpha]^{26}_D$ = -1.92 (c = 1, $CHCl_3$); ^{1}H NMR ($CDCl_3$, 400 MHz) δ: 1.39 (s, 9H), 5.85 (s, 1H), 5.98 (d, *J* = 10.8 Hz, 1H), 6.74 (t, *J* = 9.4 Hz, 1H), 6.83 (d, *J* = 7.6 Hz, 2H), 7.10–7.23 (m, 6H), 7.32–7.57 (m, 3H), 8.00–8.26 (m, 1H); ^{13}C NMR ($CDCl_3$, 100 MHz) δ: 28.2, 81.5, 111.4 (d, *J* = 12.5 Hz), 120.2 (d, *J* = 3.8 Hz), 120.7 (d, *J* = 4.7 Hz), 125.6 (d, *J* = 3.8 Hz), 125.8, 126.0, 126.4, 128.2 (d, *J* = 3.8 Hz), 129.7, 129.9, 140.1 (d, *J* = 7.8 Hz), 149.9 (dd, J = 6.8, 9.6 Hz), 154.0, 171.8; ^{31}P NMR ($CDCl_3$, 162 MHz) δ: 5.98; HPLC (70 : 30, *n*-hexane : *i*-PrOH,

254nm, 1mL/min) Chiralpak AD-H column, t_R = 11.7 (major), t_R = 29.0 (minor), 73% ee.

(*R*)-*tert*-Butyl 3-((tert-butoxycarbonyl)amino)-3-(diphenoxyphosphoryl)-2-oxoindoline-1-carboxylate (3m)

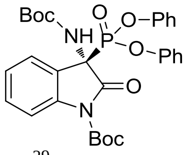

$[\alpha]^{29}_{D}$ = -17.4 (c = 1, $CHCl_3$); ^{1}H NMR ($CDCl_3$, 400 MHz) δ: 1.40 (s, 9H), 1.51 (s, 9H), 5.87 (d, *J* = 8.8 Hz, 1H), 6.96-7.12 (m, 4H), 7.18-7.24 (m, 2H), 7.29–7.44 (m, 7H), 7.88 (d, *J* = 7.6 Hz, 1H); ^{13}C NMR ($CDCl_3$, 100 MHz) δ: 27.5, 28.0, 80.2, 83.8, 119.7 (d, *J* = 4.8 Hz), 120.1 (d, *J* = 4.8 Hz), 122.3, 123.3, 125.2, 125.3, 128.3, 129.4, 129.5, 129.8, 149.7 (d, *J* = 7.6 Hz), 150.1 (d, *J* = 7.6 Hz), 166.6 (d, *J* = 5.7 Hz); ^{31}P NMR ($CDCl_3$, 162 MHz) δ: -12.50; HPLC (97 : 3, *n*-hexane : *i*-PrOH, 254nm, 0.2mL/min) Chiralpak IC column, t_R = 83.4 (minor), t_R = 96.3 (major), 26% ee.

4. References

1. George, J.; Sridhar, B.; Reddy, B. V. S. *Org. Biomol. Chem.* **2014,** *12*, 1595–1602. doi:10.1039/C3OB42026D

2. Kumar, A.; Sharma, V.; Kaur, J.; Kumar, V.; Mahajan, S.; Kumar, N.; Chimni, S. S. *Tetrahedron* **2014,** *70*, 7044–7049. doi:10.1016/j.tet.2014.06.013

5. NMR spectra and HPLC chromatograms of 3.

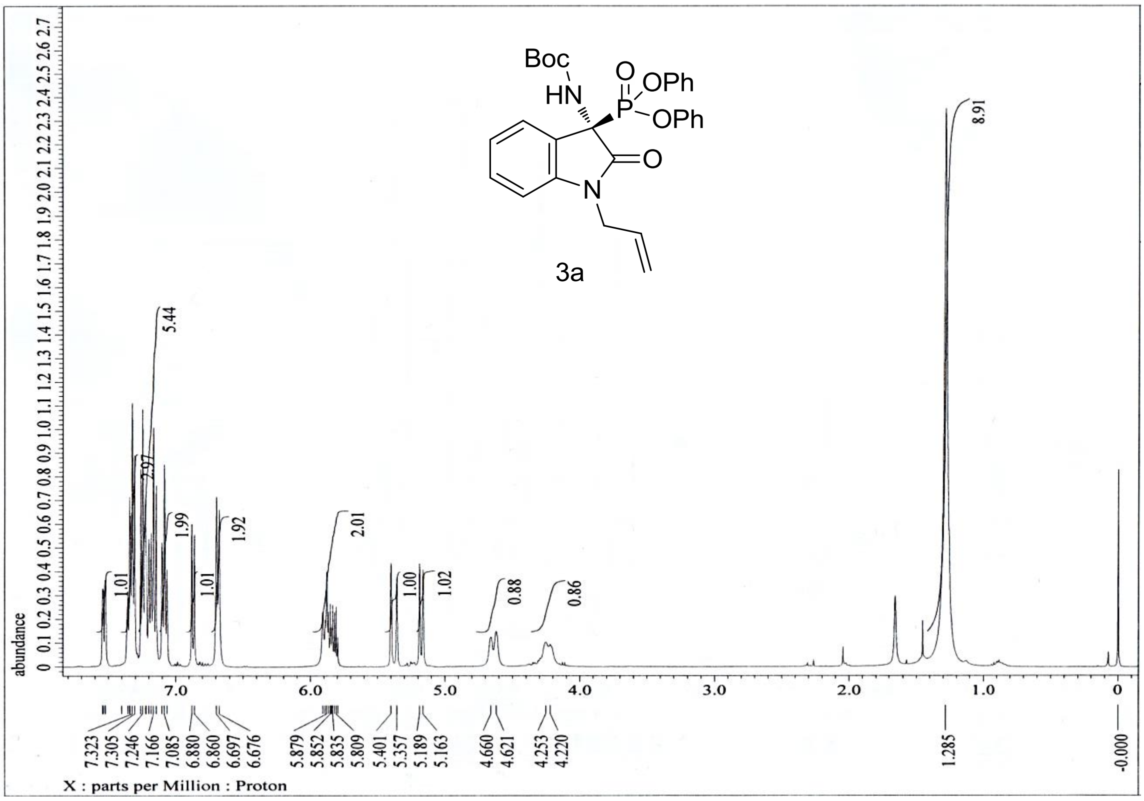

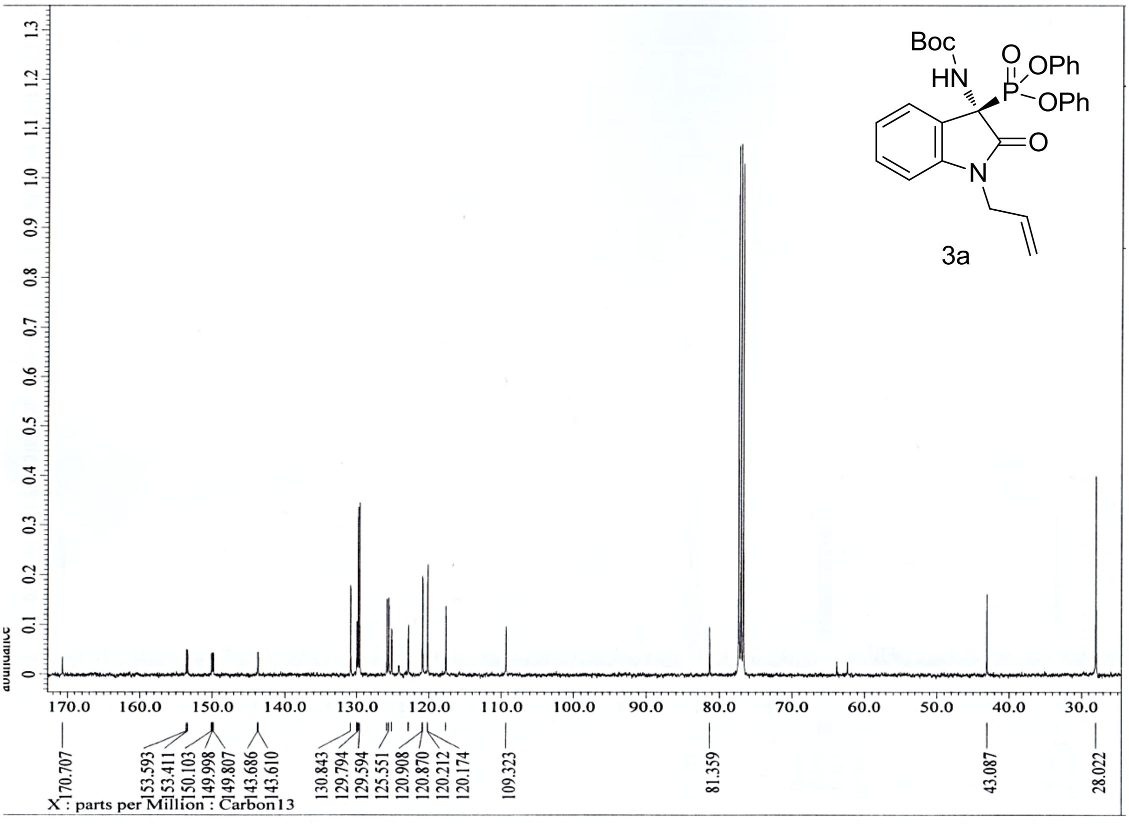

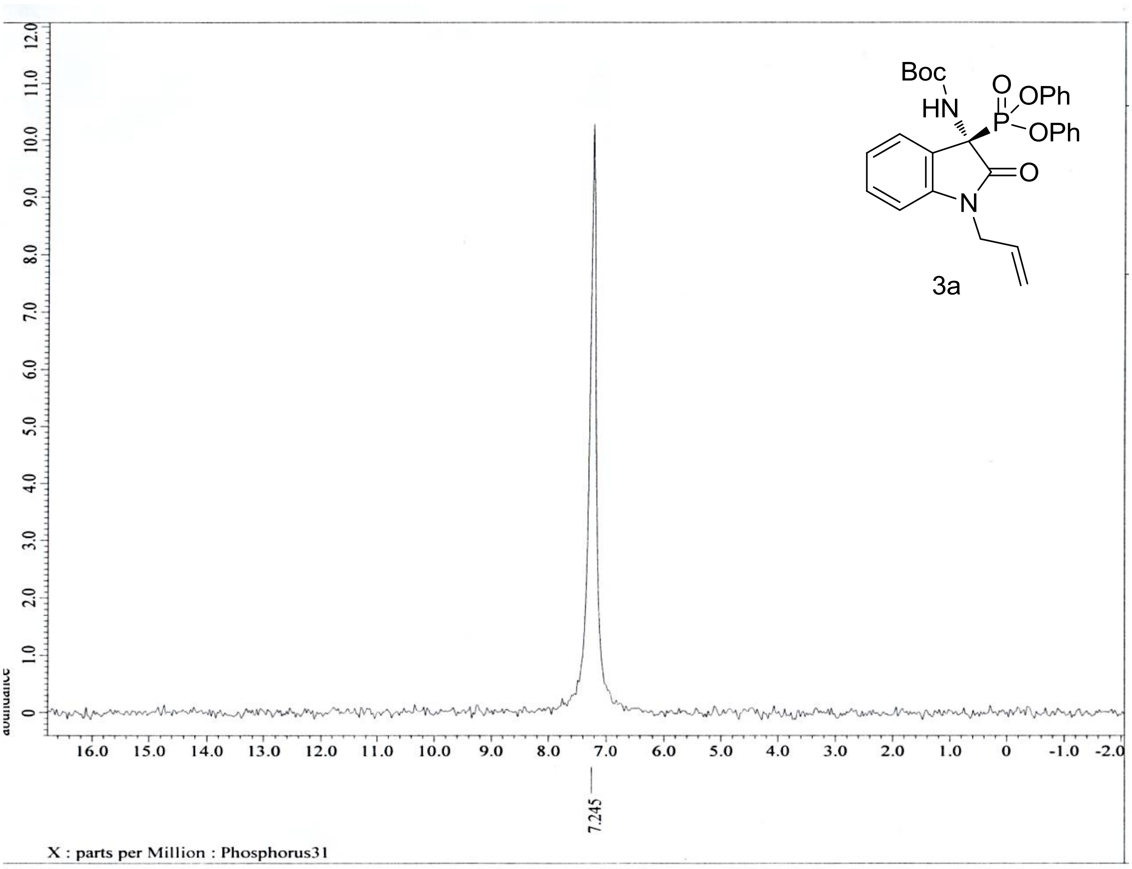
Boc
HN
O
P
OPh
OPh
O
N
3a
abundance
12.0
11.0
10.0
9.0
8.0
7.0
6.0
5.0
4.0
3.0
2.0
1.0
0
16.0
15.0
14.0
13.0
12.0
11.0
10.0
9.0
8.0
7.0
6.0
5.0
4.0
3.0
2.0
1.0
0
-1.0
-2.0
7.245
X : parts per Million : Phosphorus31

크로마토그램

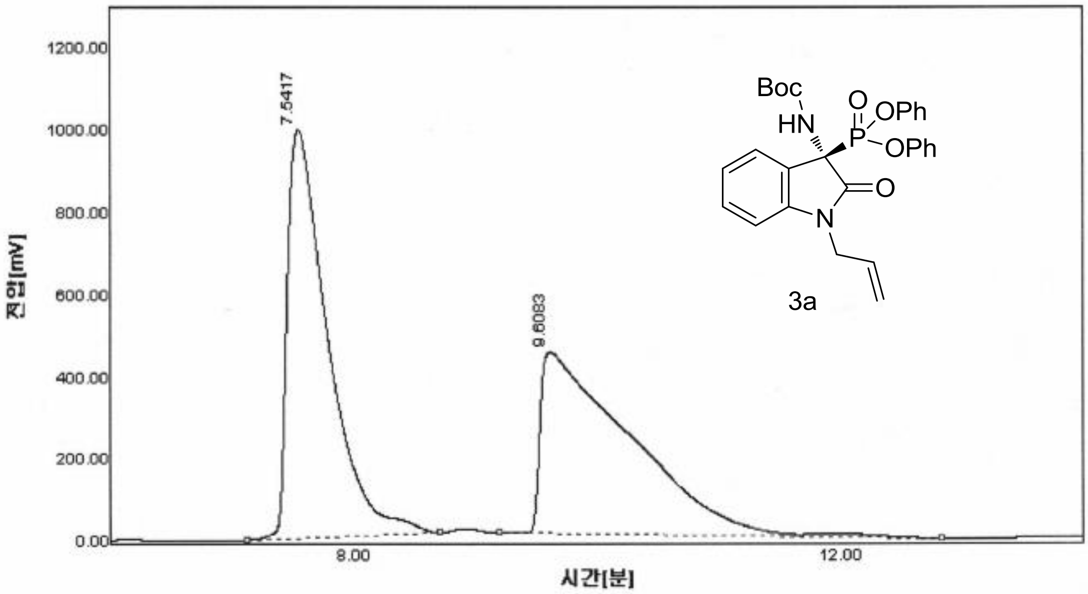

적분 결과

번호	RT[분]	면적비[%]	면적[mV*s]	폭[초]	형태
1	7.5417	50.02	22894.0781	31.35	FF
2	9.6083	49.98	22880.1125	99.39	FF
합계			45774.1906		

크로마토그램

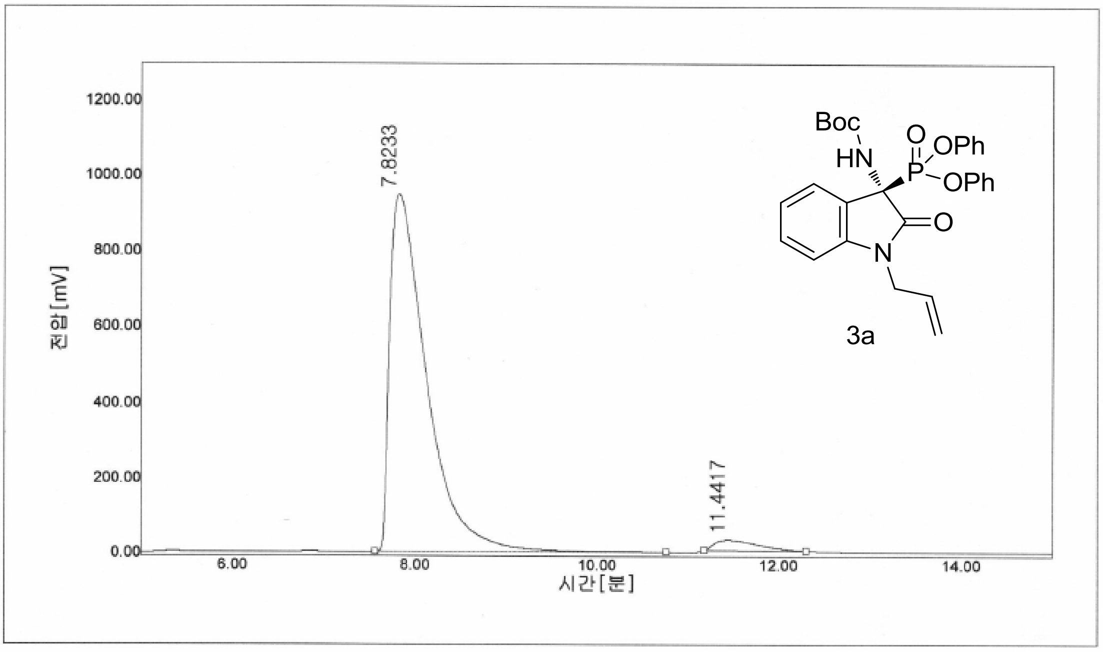

적분 결과

번호	RT[분]	면적비[%]	면적[mV*s]	폭[초]	형태
1	7.8233	96.52	26705.6469	37.48	FF
2	11.4417	3.48	964.2119	53.79	FF
합계			27669.8594		

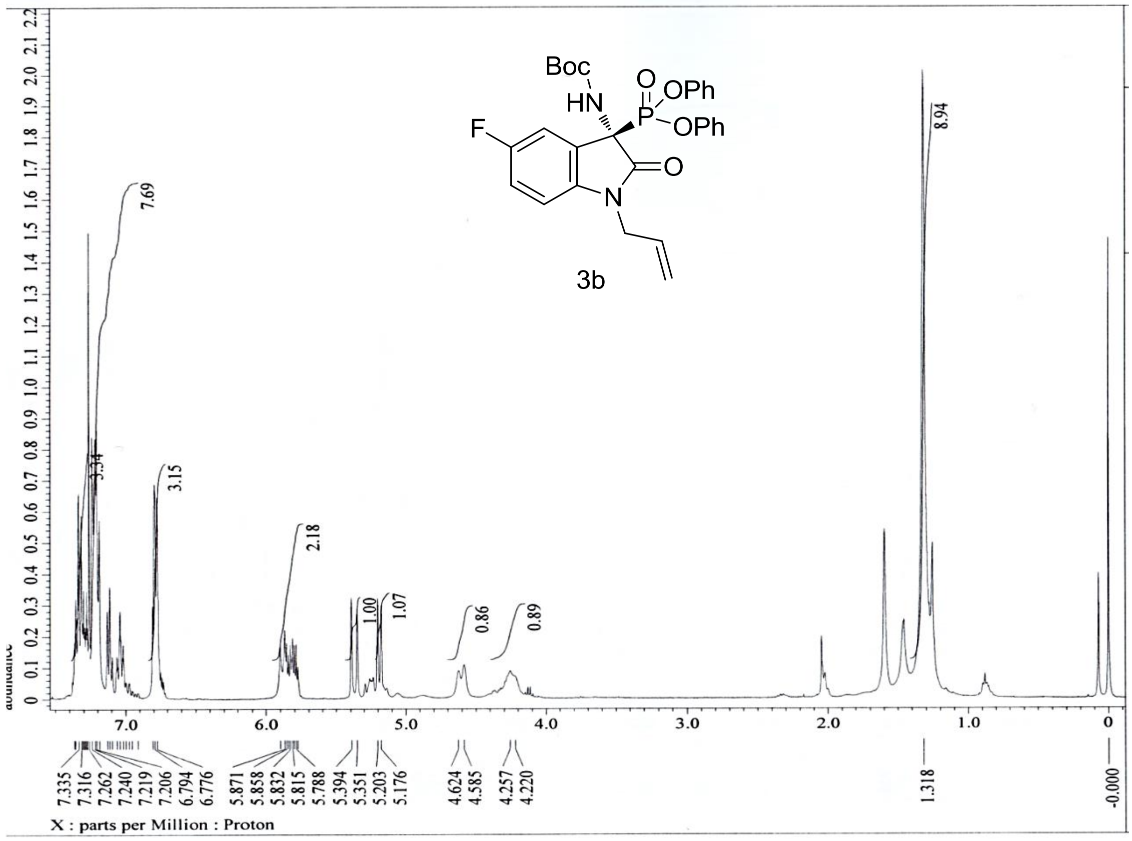

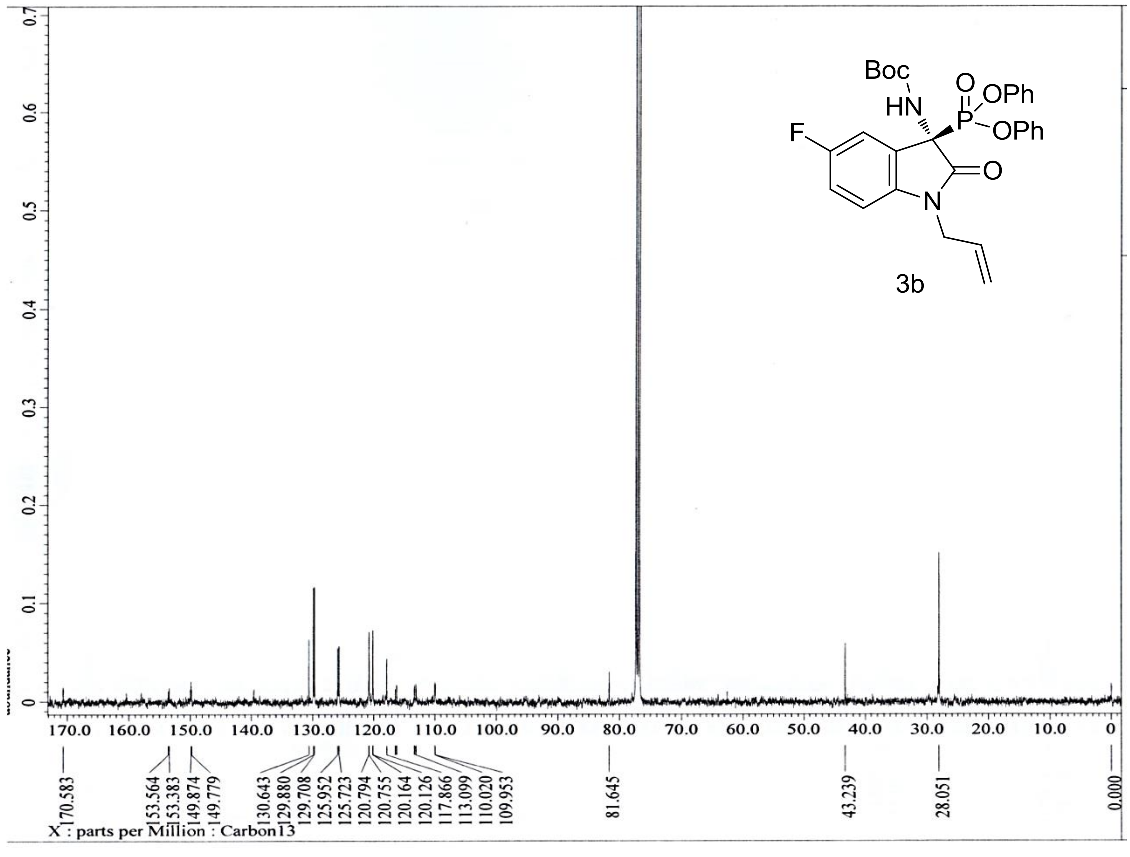

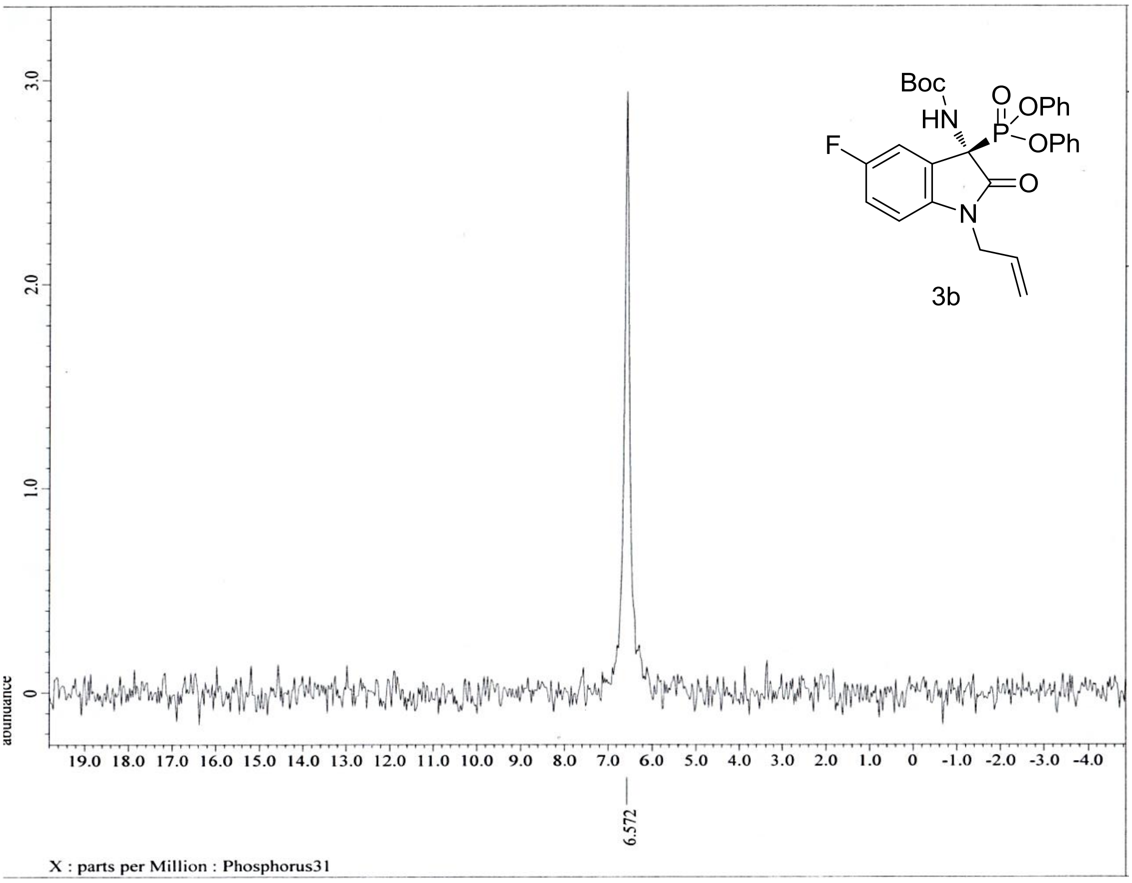

Boc
HN
O
P
OPh
OPh
F
O
N
3b
3.0
2.0
1.0
0
abundance
19.0 18.0 17.0 16.0 15.0 14.0 13.0 12.0 11.0 10.0 9.0 8.0 7.0 6.0 5.0 4.0 3.0 2.0 1.0 0 -1.0 -2.0 -3.0 -4.0
6.572
X : parts per Million : Phosphorus31

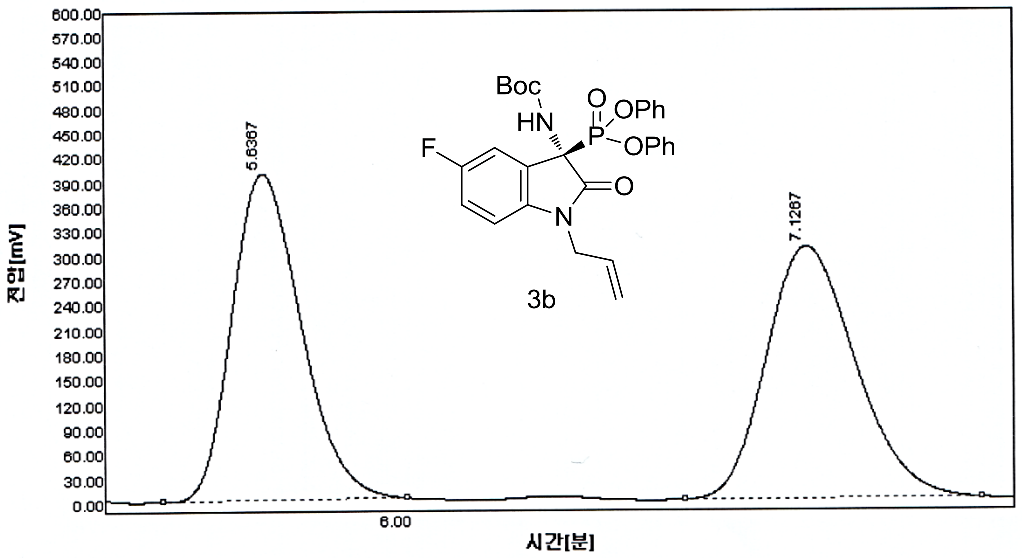

적분 결과

번호	RT[분]	면적비[%]	면적[mV*s]	폭[초]	형태
1	5.6367	49.94	5179.4809	20.63	FF
2	7.1267	50.06	5191.4945	26.82	FF
합계			10370.9754		

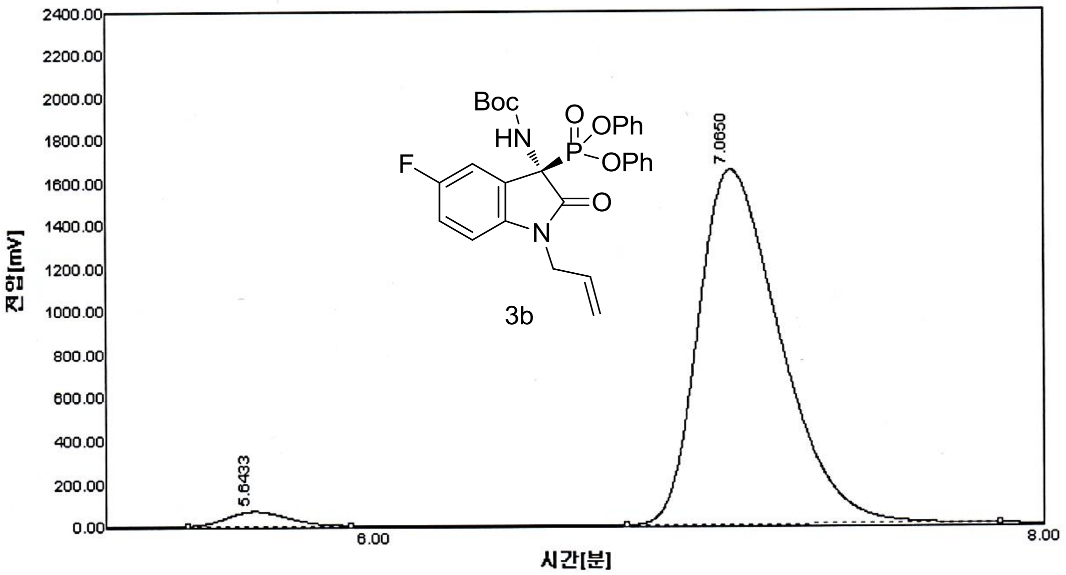

적분 결과

번호	RT[분]	면적비[%]	면적[mV*s]	폭[초]	형태
1	5.6433	2.85	816.2968	19.57	FF
2	7.0650	97.15	27816.0687	23.99	FF
합계			28632.3655		

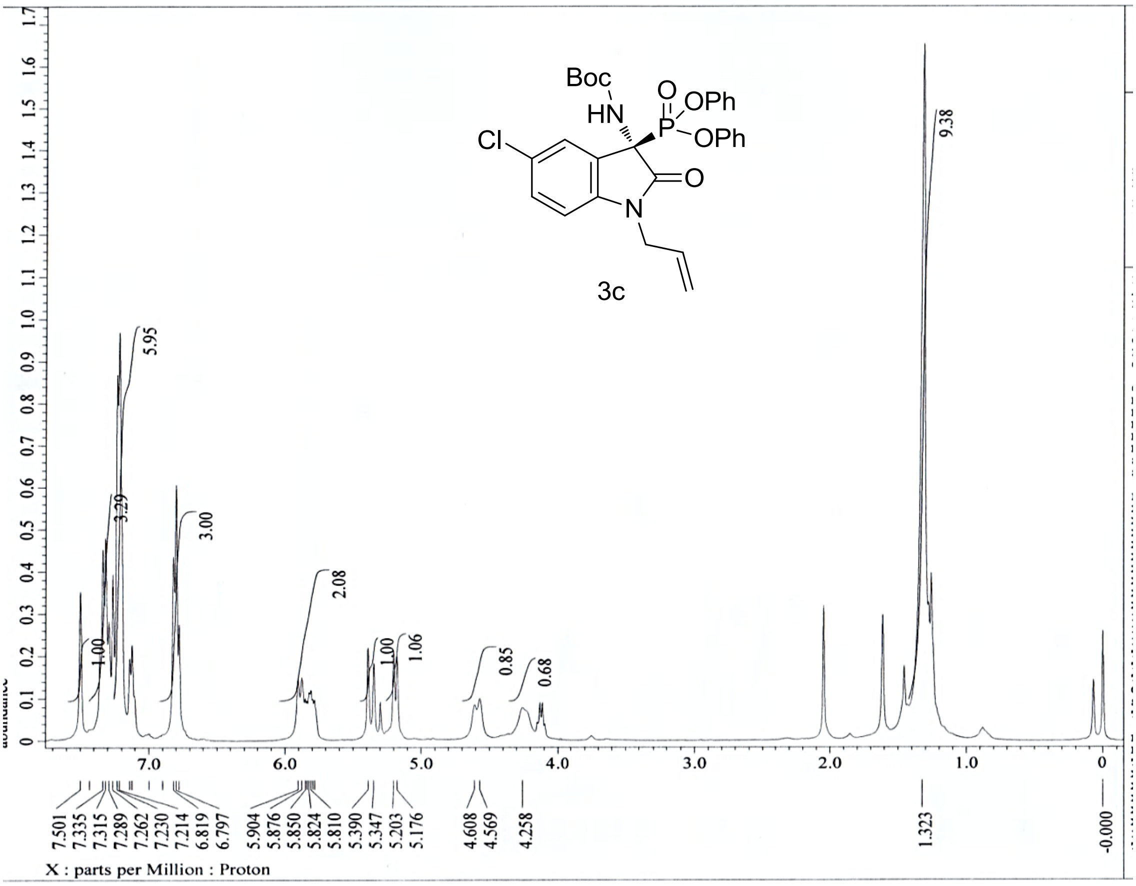
Boc
HN
O
P
OPh
OPh
Cl
O
N
3c
9.38
5.95
3.29
3.00
2.08
1.00
1.00
1.06
0.85
0.68
abundance
7.0
6.0
5.0
4.0
3.0
2.0
1.0
0
7.501
7.335
7.315
7.289
7.262
7.230
7.214
6.819
6.797
5.904
5.876
5.850
5.824
5.810
5.390
5.347
5.203
5.176
4.608
4.569
4.258
1.323
-0.000
X : parts per Million : Proton

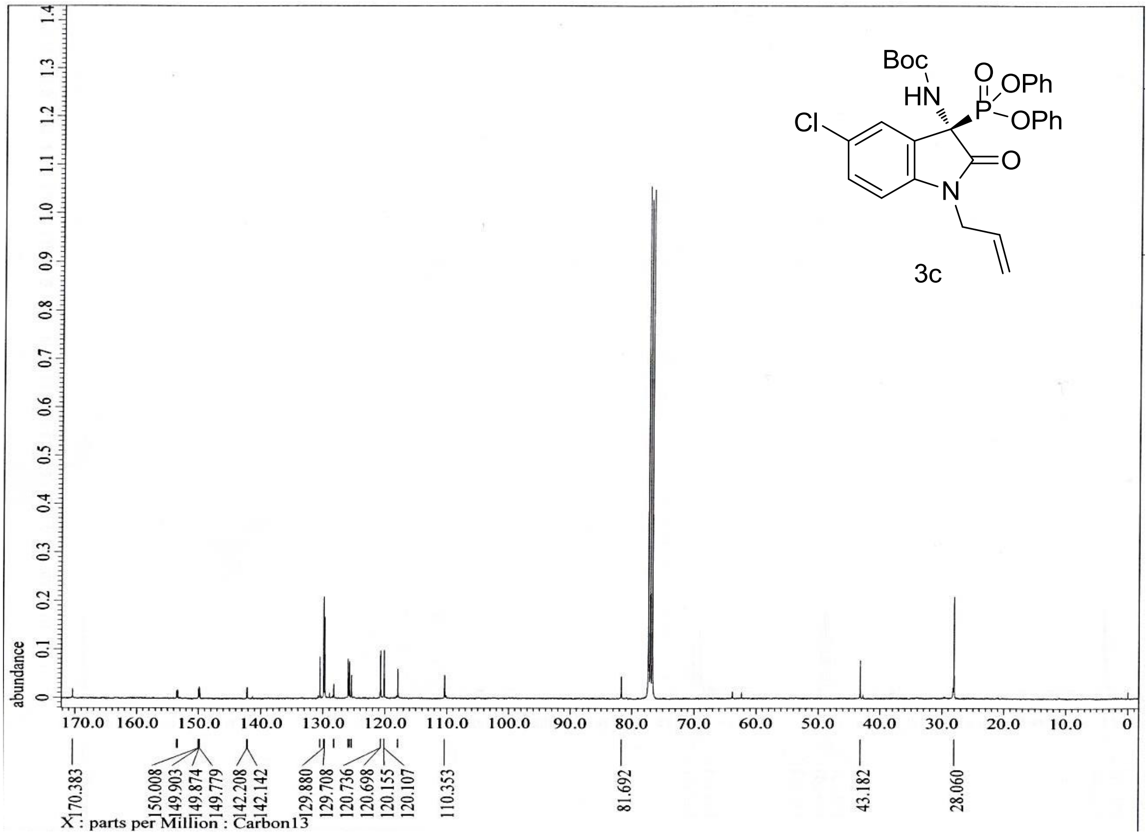
Boc
HN
O
P
OPh
OPh
Cl
O
N
3c
abundance
170.0
160.0
150.0
140.0
130.0
120.0
110.0
100.0
90.0
80.0
70.0
60.0
50.0
40.0
30.0
20.0
10.0
0
170.383
150.008
149.903
149.874
149.779
142.208
142.142
129.880
129.708
120.736
120.698
120.155
120.107
110.353
81.692
43.182
28.060
X : parts per Million : Carbon13

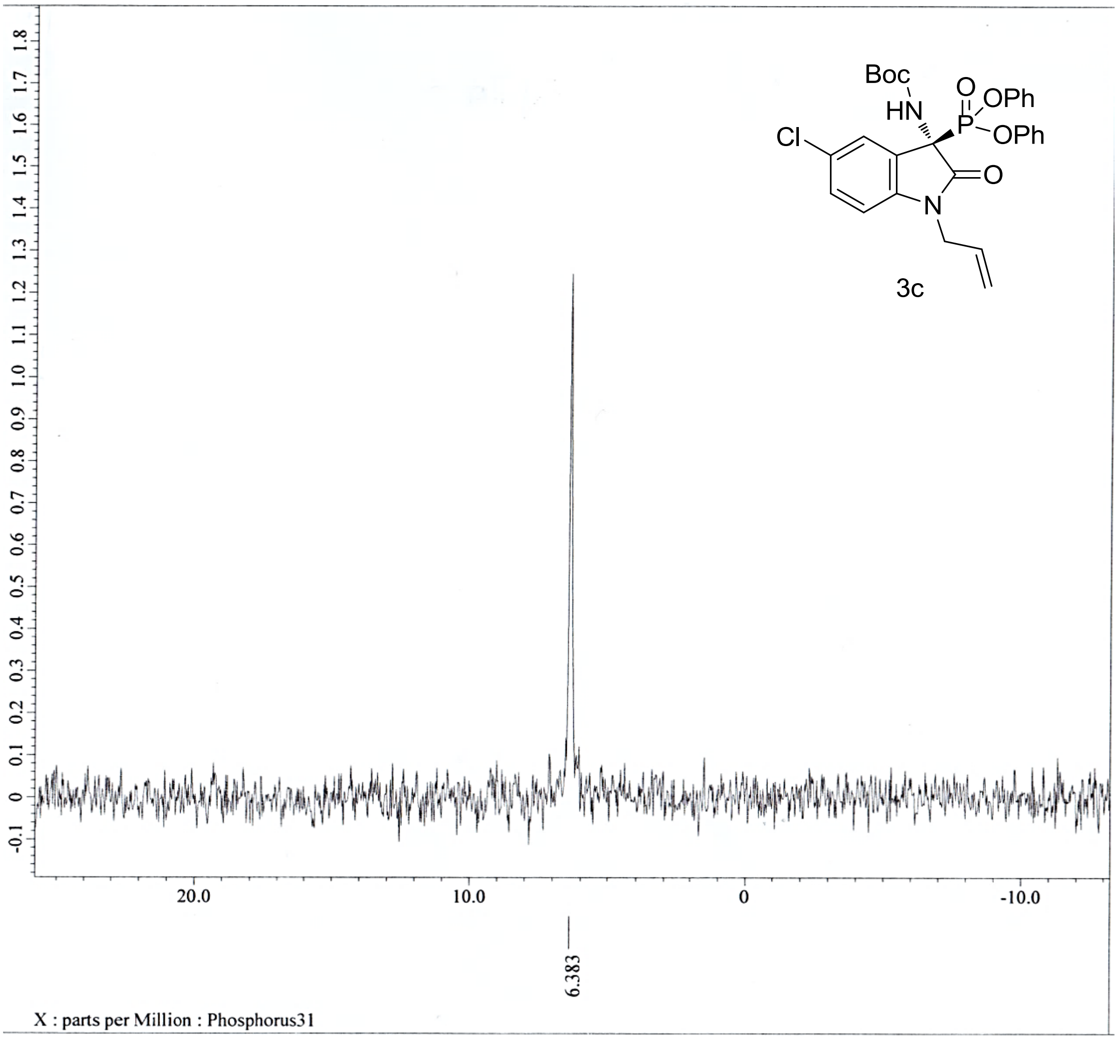
Boc
HN
O
OPh
P
OPh
Cl
O
N
3c
1.8
1.7
1.6
1.5
1.4
1.3
1.2
1.1
1.0
0.9
0.8
0.7
0.6
0.5
0.4
0.3
0.2
0.1
0
-0.1
20.0
10.0
0
-10.0
6.383
X : parts per Million : Phosphorus31

크로마토그램

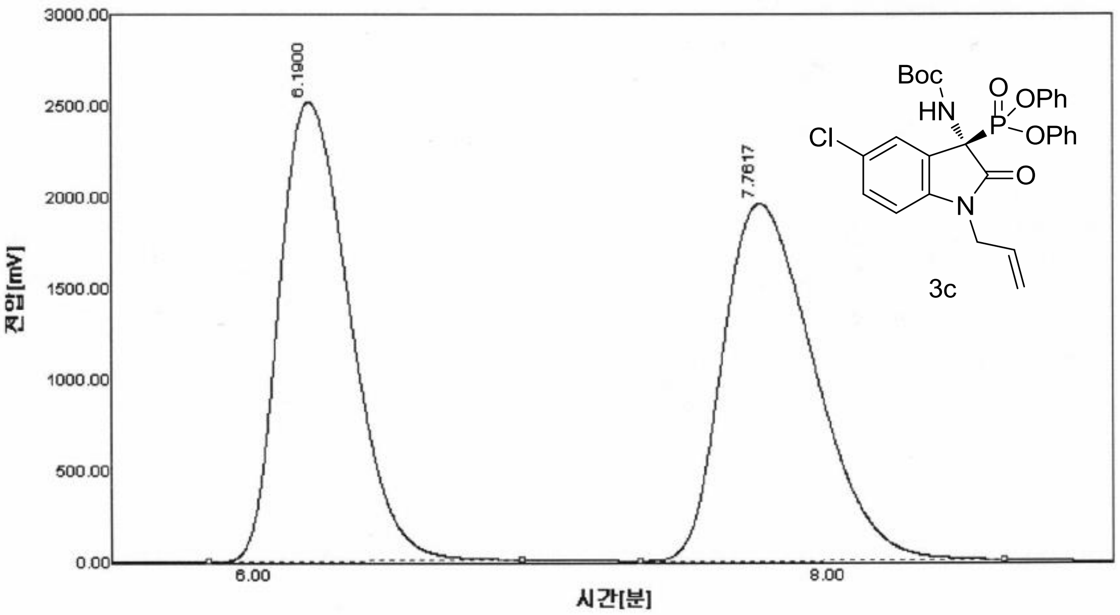

적분 결과

번호	RT[분]	면적비[%]	면적[mV*s]	폭[초]	형태
1	6.1900	49.70	42639.7531	26.60	FF
2	7.7617	50.30	43155.6281	35.05	FF
합계			85795.3812		

크로마토그램

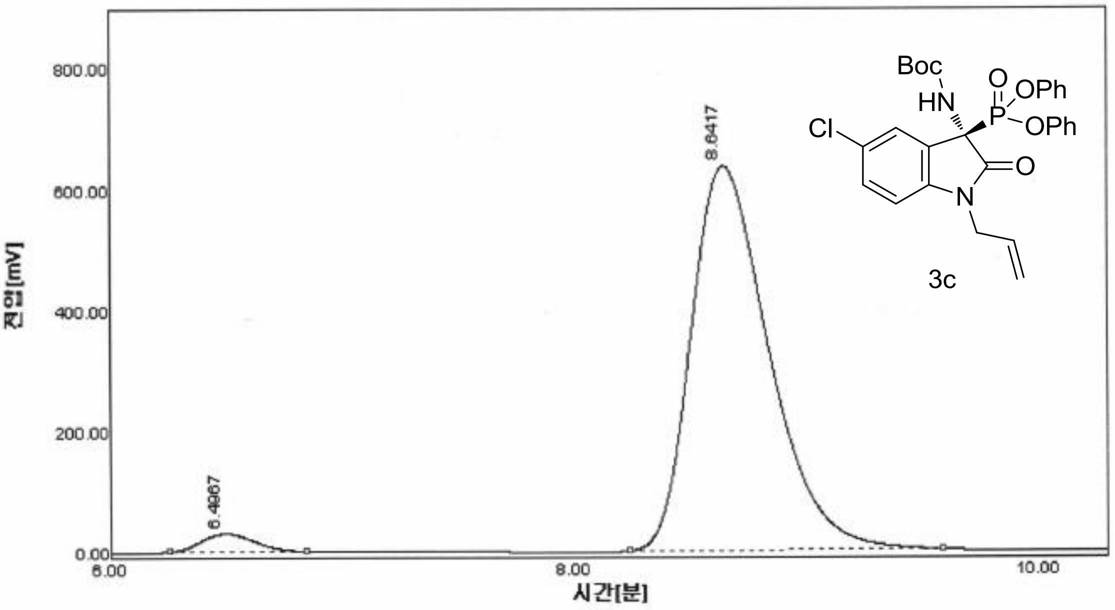

적분 결과

번호	RT[분]	면적비[%]	면적[mV*s]	폭[초]	형태
1	6.4967	3.07	466.6148	25.28	FF
2	8.6417	96.93	14752.4875	33.05	FF
합계			15219.1023		

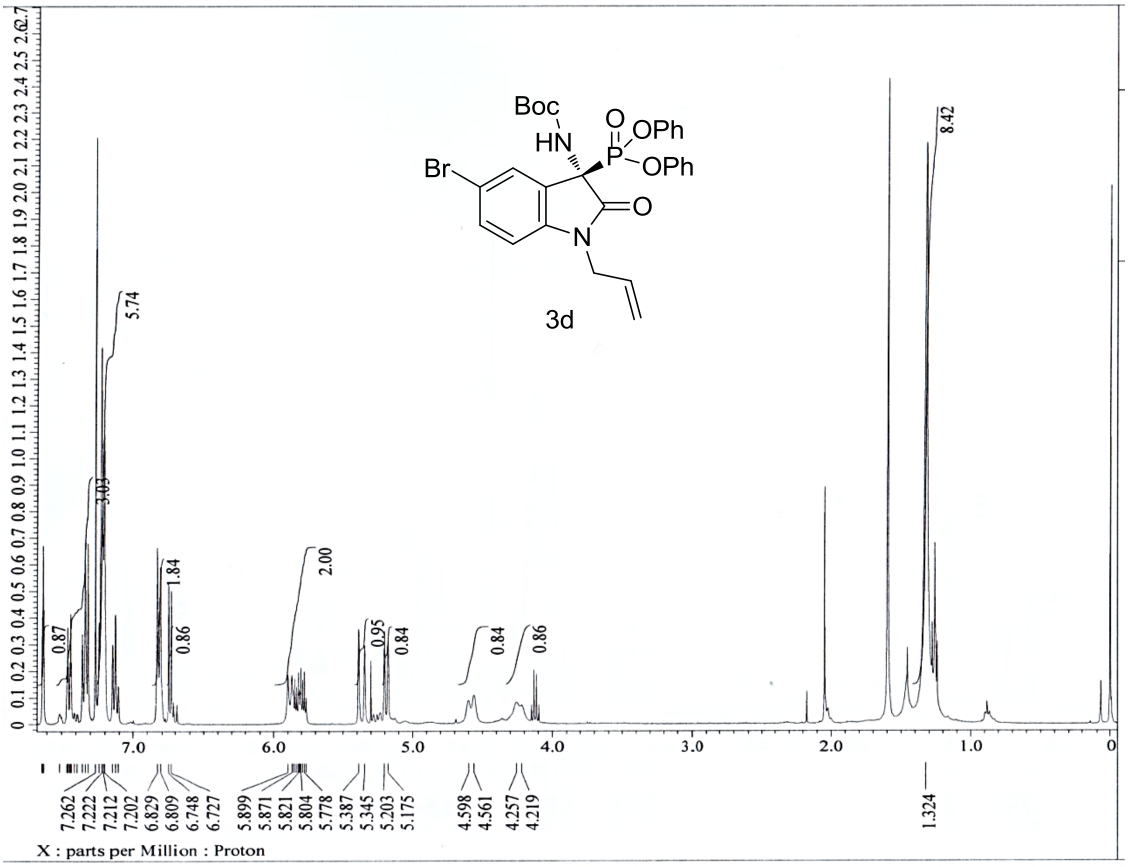
Boc
HN
O
OPh
P
OPh
Br
O
N
3d
5.74
3.03
1.84
0.87
0.86
2.00
0.95
0.84
0.84
0.86
8.42
7.262
7.222
7.212
7.202
6.829
6.809
6.748
6.727
5.899
5.871
5.821
5.804
5.778
5.387
5.345
5.203
5.175
4.598
4.561
4.257
4.219
1.324
X : parts per Million : Proton

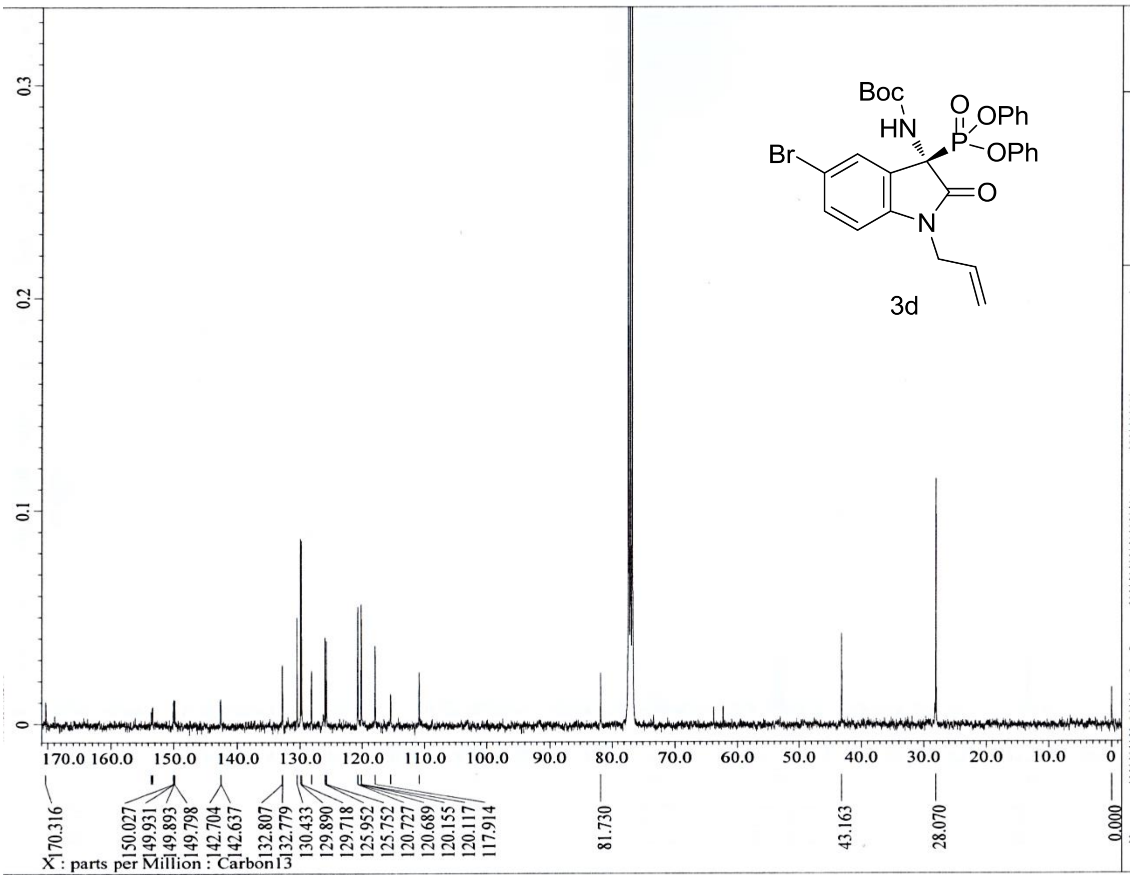
Boc
HN
O
OPh
P
OPh
Br
O
N
3d
170.316
150.027
149.931
149.893
149.798
142.704
142.637
132.807
132.779
130.433
129.890
129.718
125.952
125.752
120.727
120.689
120.155
120.117
117.914
81.730
43.163
28.070
0.000
X : parts per Million : Carbon13

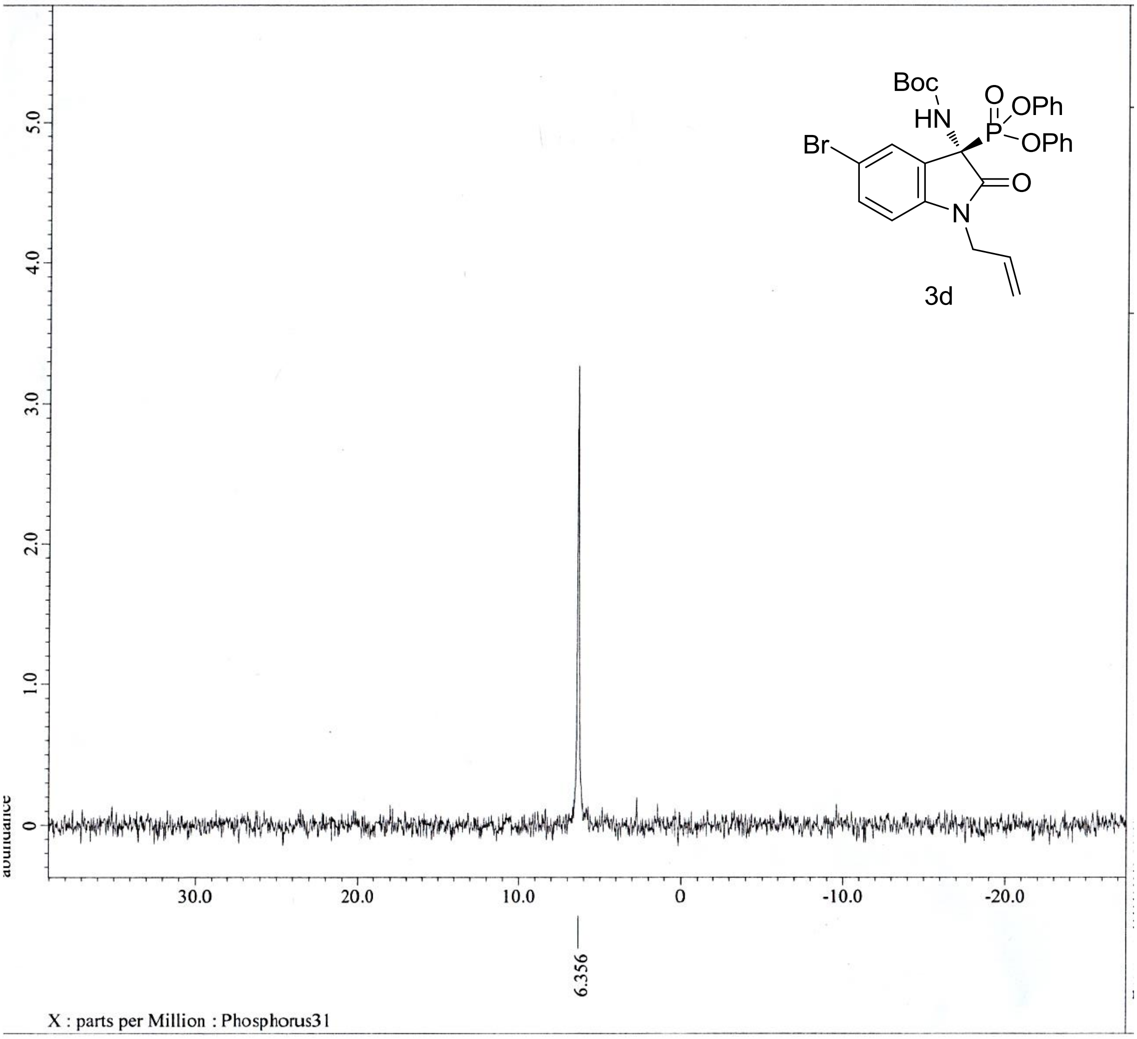
Boc
HN
O
OPh
P
OPh
Br
O
N
3d
abundance
0
1.0
2.0
3.0
4.0
5.0
30.0
20.0
10.0
0
-10.0
-20.0
6.356
X : parts per Million : Phosphorus31

<Chromatogram>

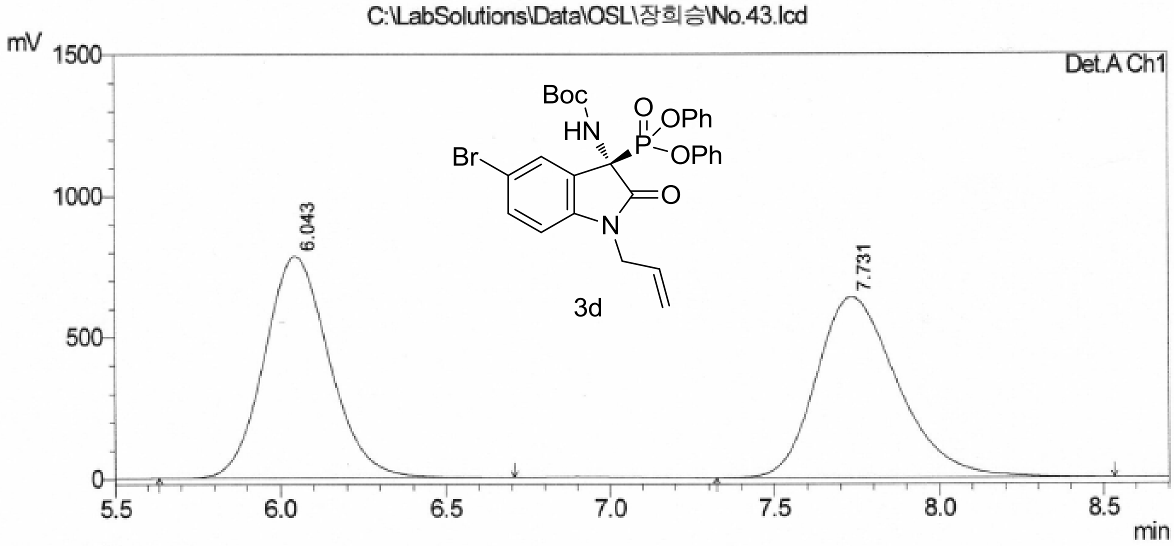

1 Det.A Ch1/254nm

PeakTable

Detector A Ch1 254nm

Peak#	Ret. Time	Area	Height	Area %	Height %
1	6.043	10625675	782521	49.329	55.120
2	7.731	10914619	637152	50.671	44.880
Total		21540293	1419672	100.000	100.000

<Chromatogram>

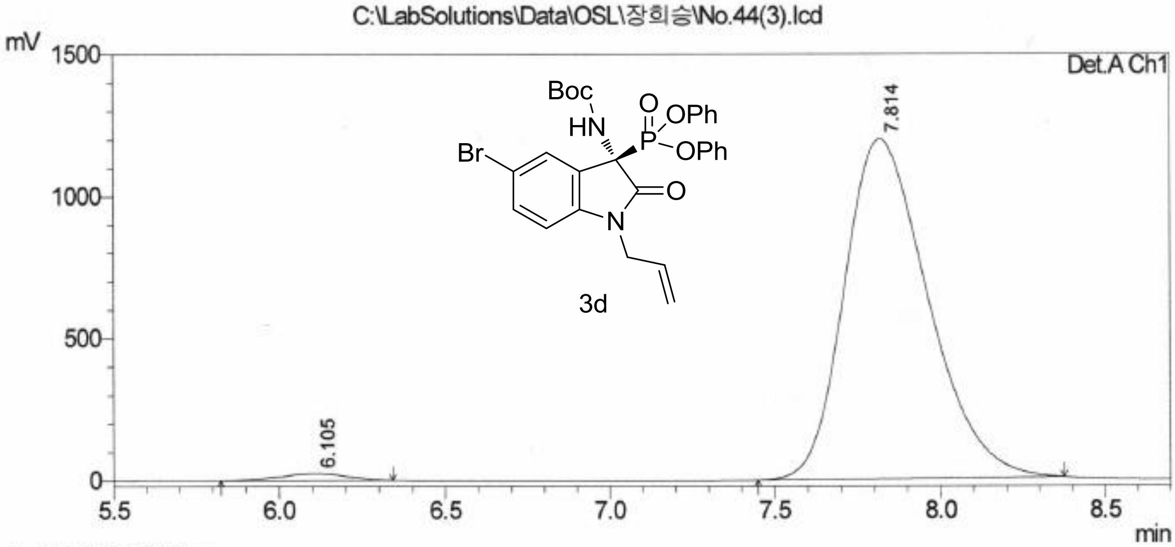

1 Det.A Ch1/254nm

PeakTable

Detector A Ch1 254nm

Peak#	Ret. Time	Area	Height	Area %	Height %
1	6.105	339344	24603	1.592	2.014
2	7.814	20972794	1196913	98.408	97.986
Total		21312137	1221516	100.000	100.000

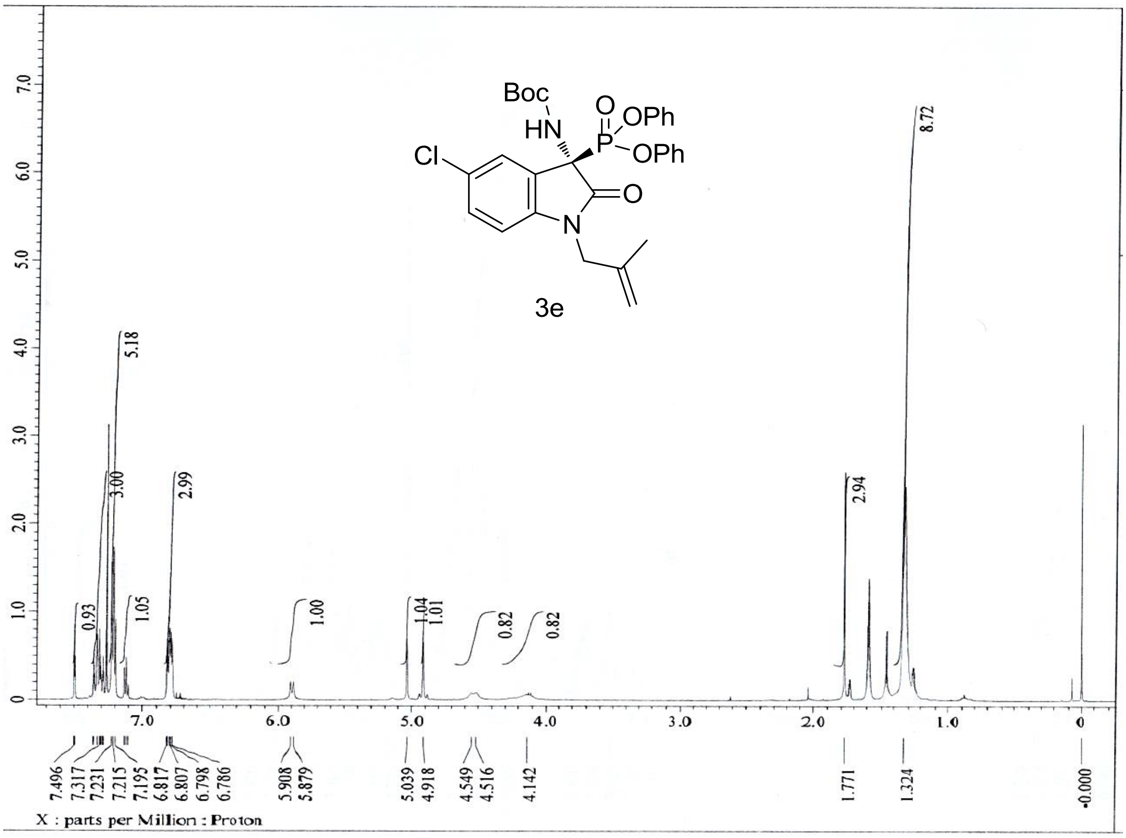
Boc
HN
O
OPh
P
OPh
Cl
O
N
3e
8.72
5.18
3.00
2.99
2.94
1.05
0.93
1.00
1.04
1.01
0.82
0.82
7.496
7.317
7.231
7.215
7.195
6.817
6.807
6.798
6.786
5.908
5.879
5.039
4.918
4.549
4.516
4.142
1.771
1.324
-0.000
X : parts per Million : Proton

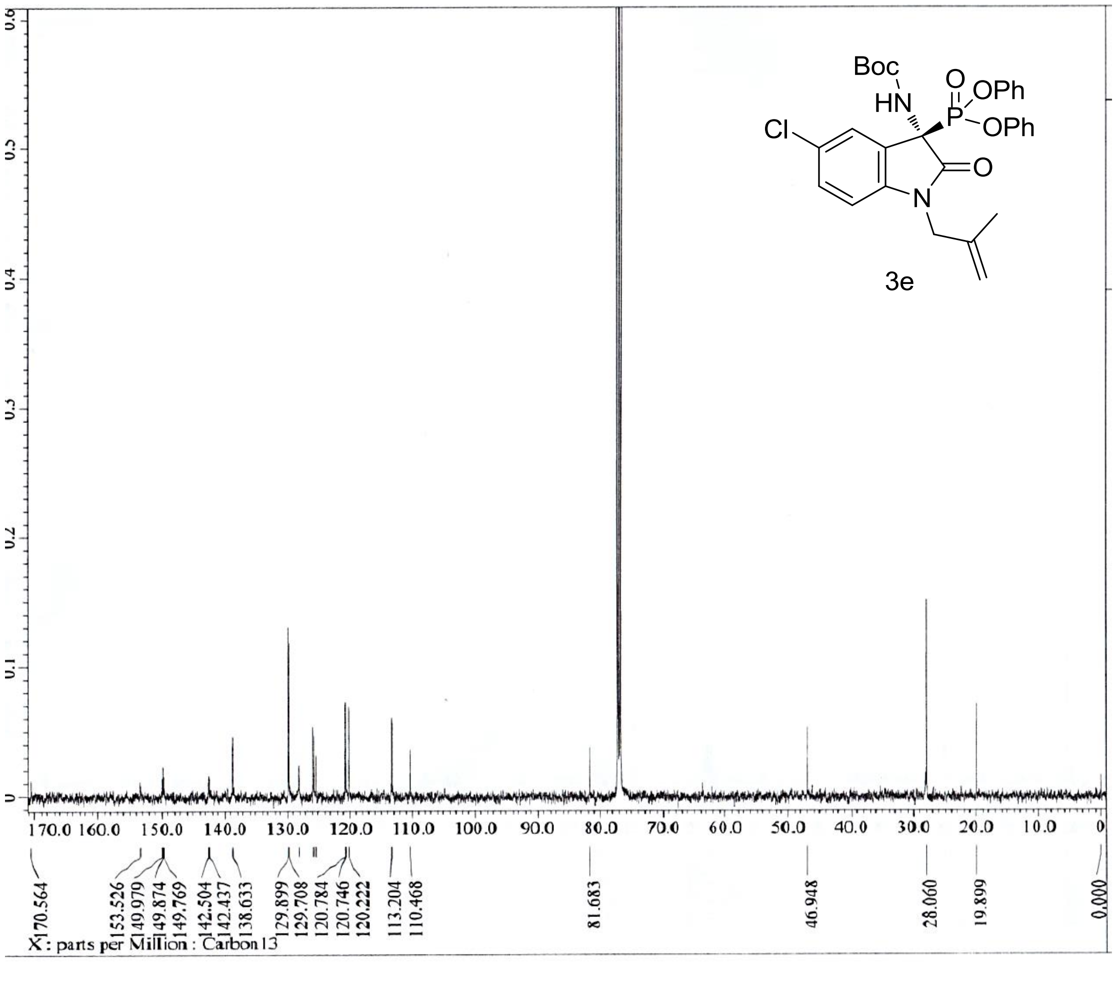
Boc
HN
O
OPh
P
OPh
Cl
O
N
3e
170.564
153.526
149.979
149.874
149.769
142.504
142.437
138.633
129.899
129.708
120.784
120.746
120.222
113.204
110.468
81.683
46.948
28.060
19.899
0.000
X : parts per Million : Carbon13

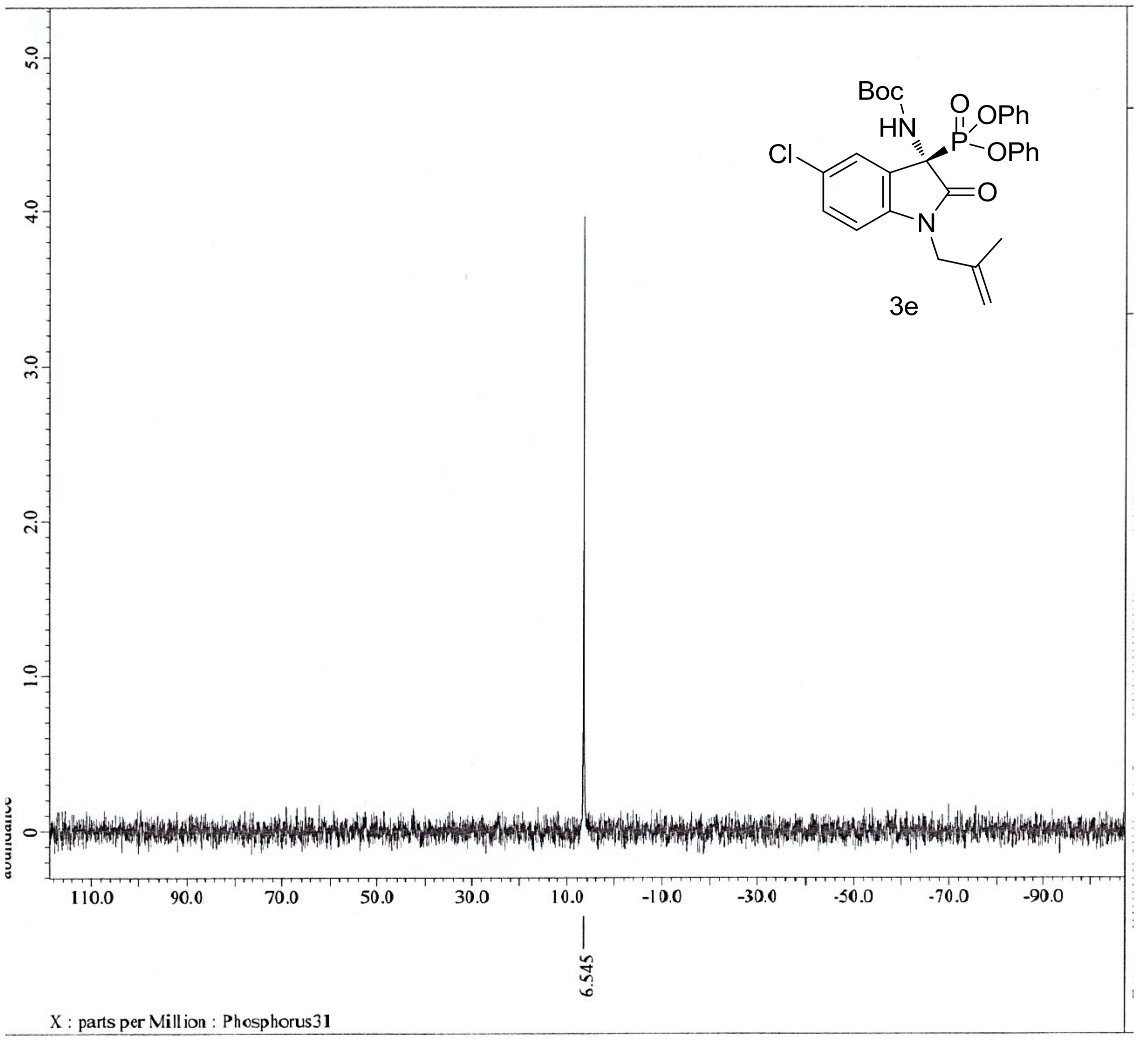
Boc
HN
O
OPh
P
OPh
Cl
O
N
3e
5.0
4.0
3.0
2.0
1.0
0
abundance
110.0
90.0
70.0
50.0
30.0
10.0
-10.0
-30.0
-50.0
-70.0
-90.0
6.545
X : parts per Million : Phosphorus31

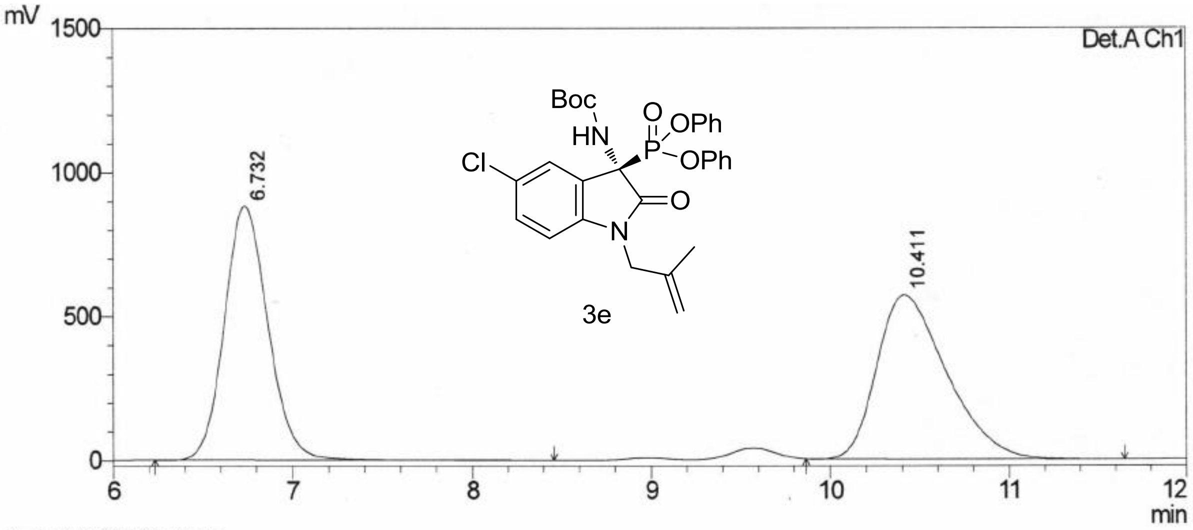

1 Det.A Ch1/254nm

PeakTable

Detector A Ch1 254nm

Peak#	Ret. Time	Area	Height	Area %	Height %
1	6.732	14823700	881646	49.020	60.728
2	10.411	15416559	570157	50.980	39.272
Total		30240259	1451803	100.000	100.000

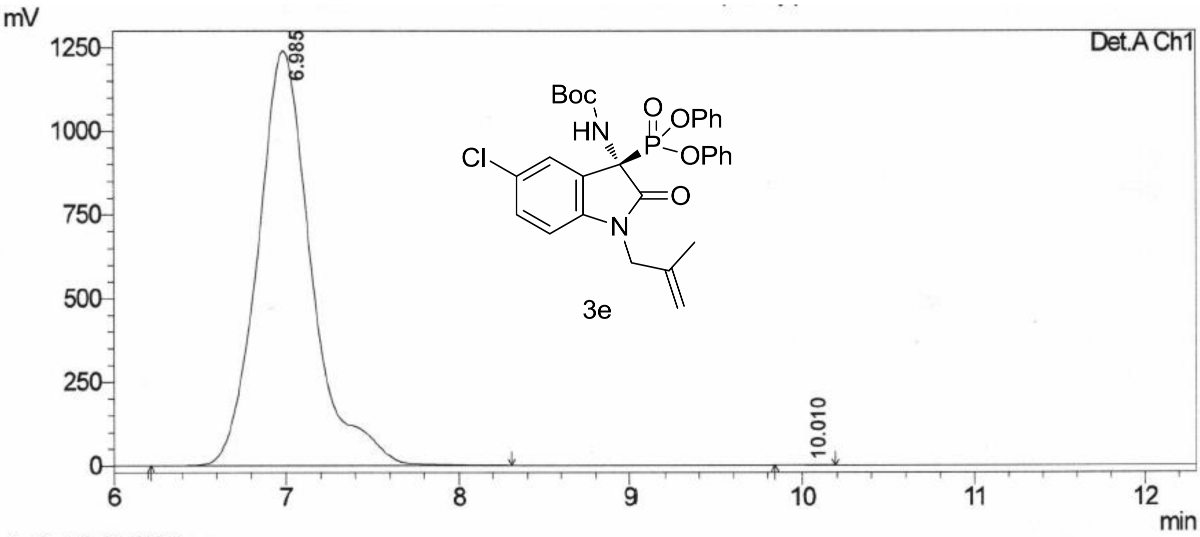

1 Det.A Ch1/254nm

PeakTable

Detector A Ch1 254nm

Peak#	Ret. Time	Area	Height	Area %	Height %
1	6.985	26576366	1239137	99.963	99.936
2	10.010	9919	788	0.037	0.064
Total		26586285	1239925	100.000	100.000

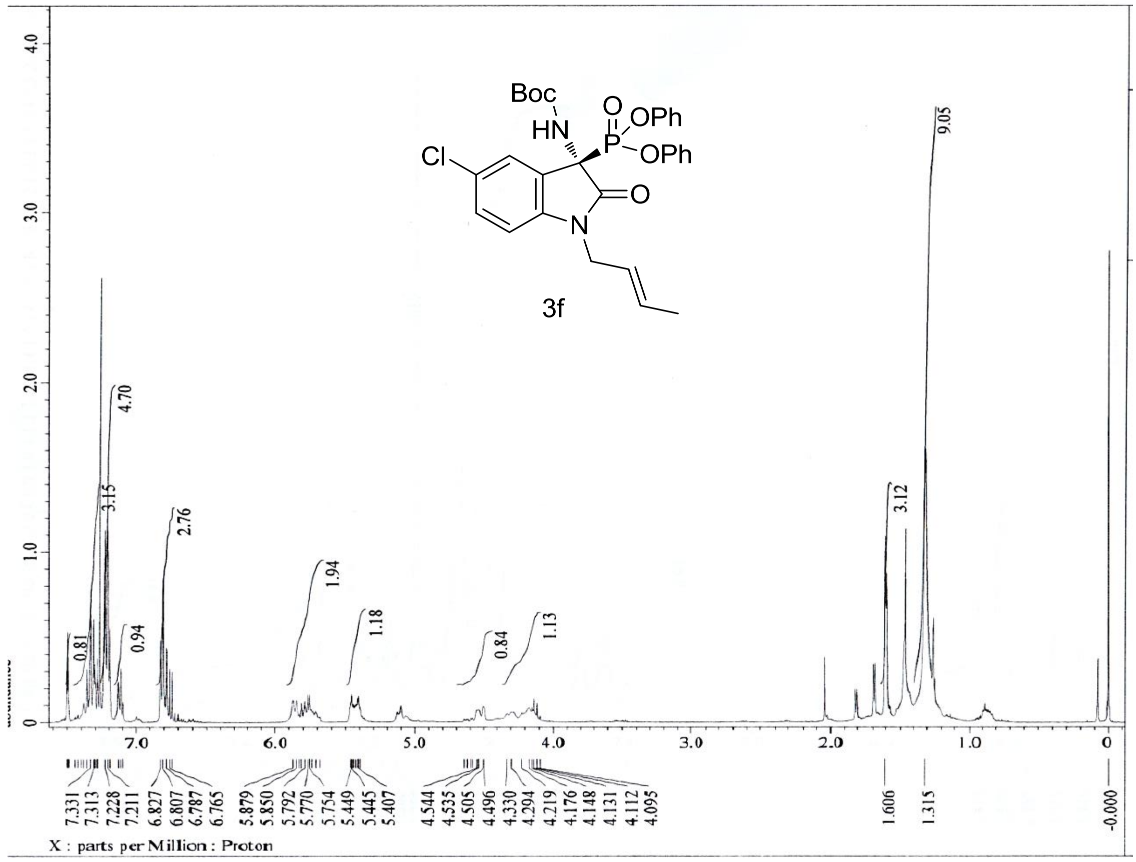
Boc
HN
O
OPh
P
OPh
Cl
O
N
3f
X : parts per Million : Proton

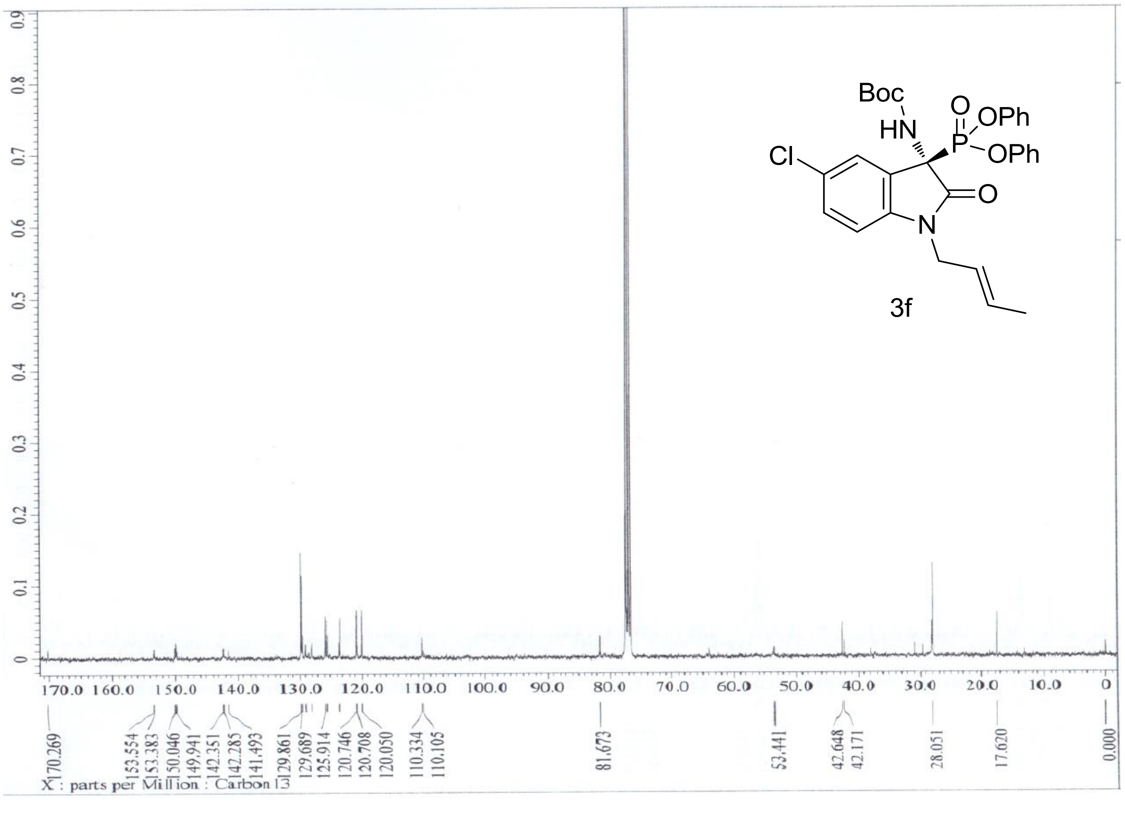
Boc
HN
O
OPh
P
OPh
Cl
O
N
3f
X : parts per Million : Carbon13

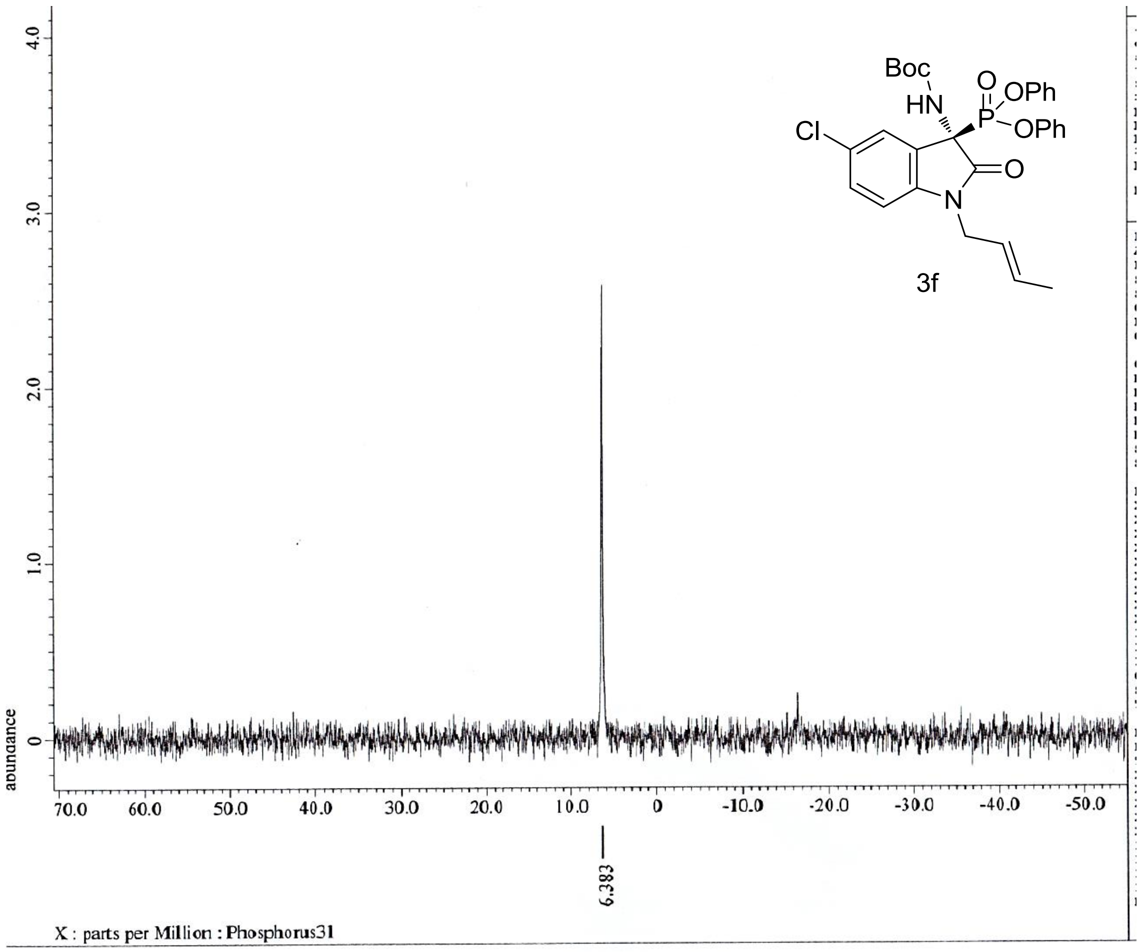
Boc
HN
O
P
OPh
OPh
Cl
O
N
3f
4.0
3.0
2.0
1.0
0
abundance
70.0
60.0
50.0
40.0
30.0
20.0
10.0
0
-10.0
-20.0
-30.0
-40.0
-50.0
6.383
X : parts per Million : Phosphorus31

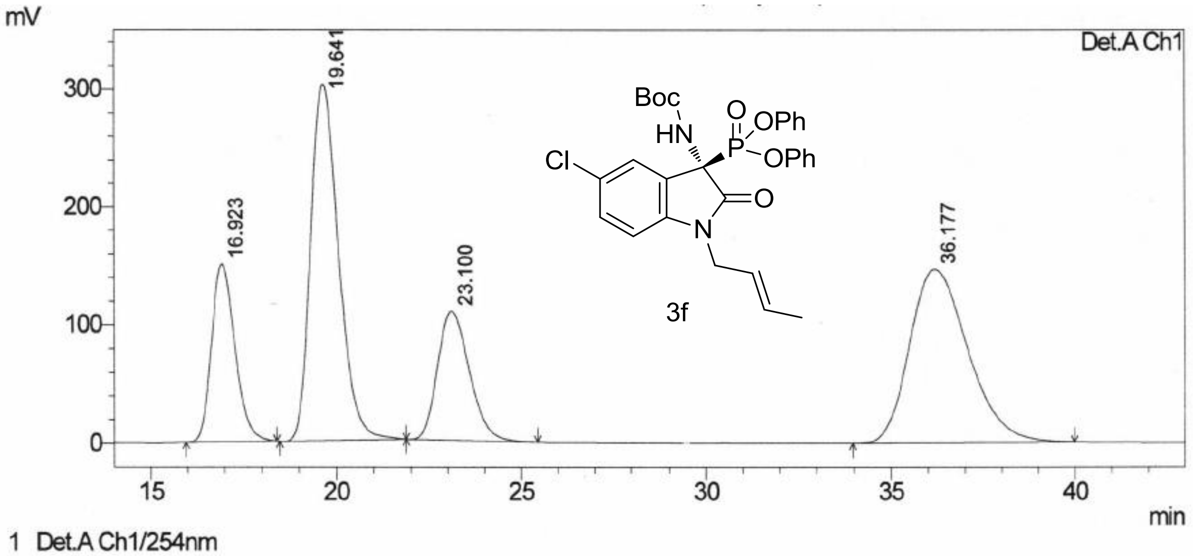

1 Det.A Ch1/254nm

PeakTable

Detector A Ch1 254nm

Peak#	Ret. Time	Area	Height	Area %	Height %
1	16.923	6618401	150146	14.733	21.219
2	19.641	15729707	302048	35.017	42.686
3	23.100	6510334	108858	14.493	15.384
4	36.177	16062385	146560	35.757	20.712
Total		44920827	707612	100.000	100.000

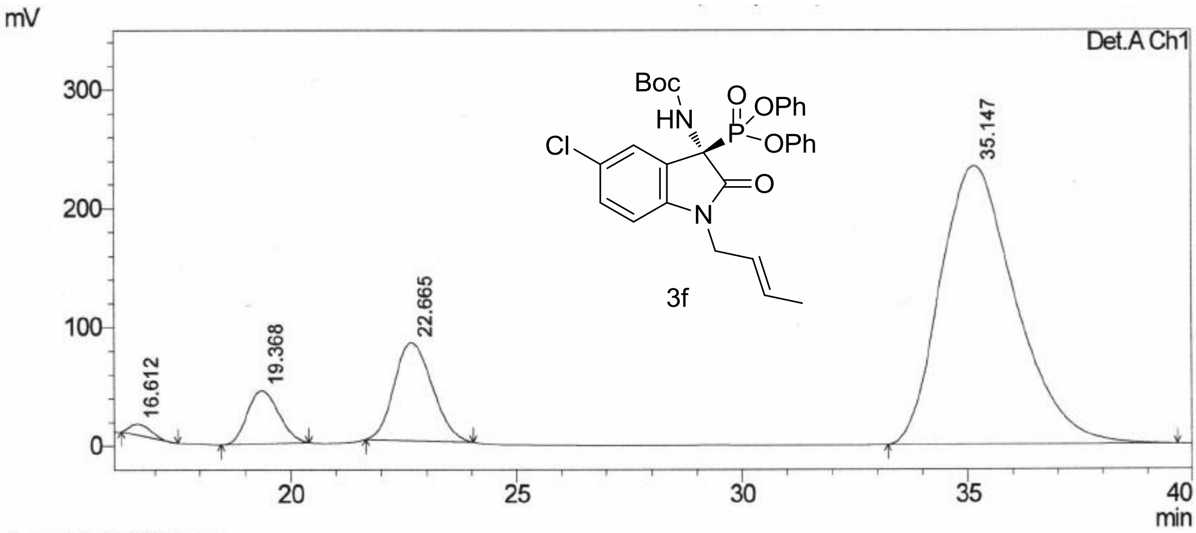

1 Det.A Ch1/254nm

PeakTable

Detector A Ch1 254nm

Peak#	Ret. Time	Area	Height	Area %	Height %
1	16.612	299402	9121	0.868	2.461
2	19.368	2142934	44614	6.210	12.039
3	22.665	4822768	82563	13.976	22.279
4	35.147	27243200	234288	78.947	63.221
Total		34508304	370587	100.000	100.000

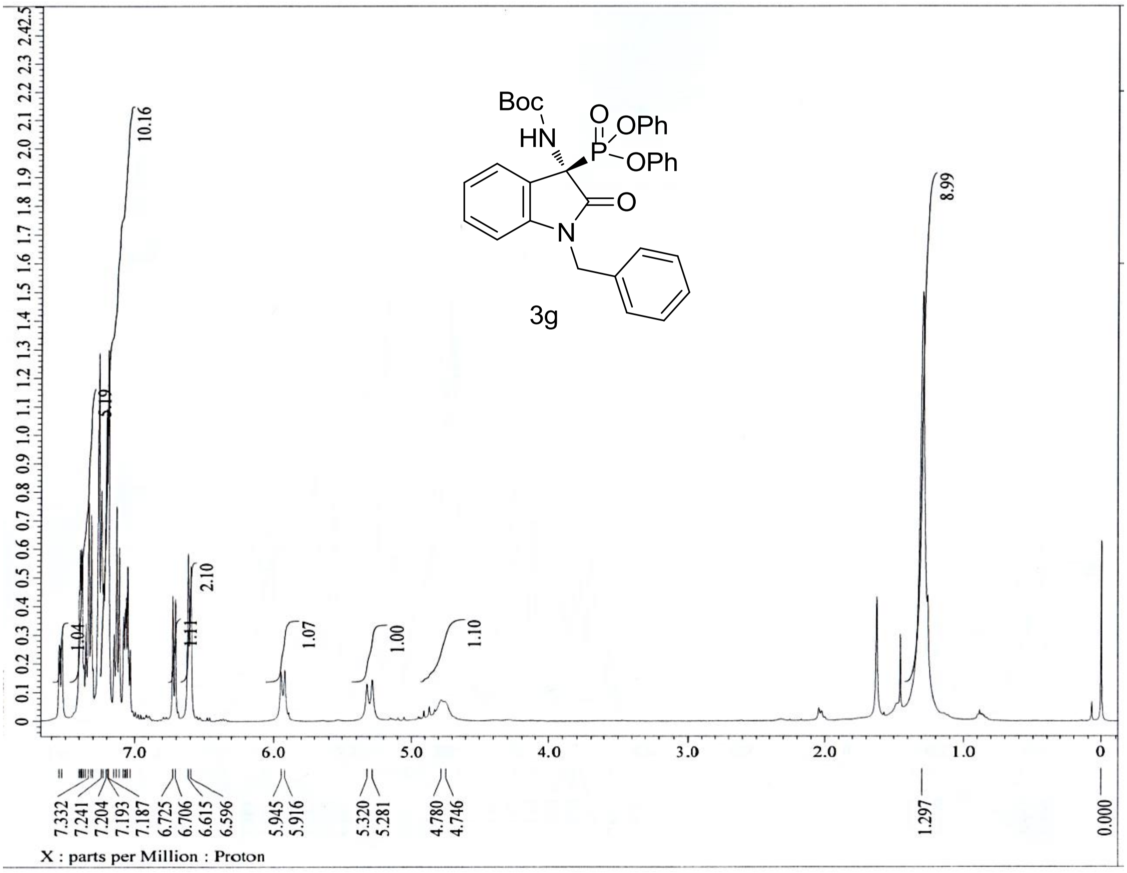
Boc
HN
O
P
OPh
OPh
O
N
3g
10.16
5.19
1.04
1.11
2.10
1.07
1.00
1.10
8.99
7.332
7.241
7.204
7.193
7.187
6.725
6.706
6.615
6.596
5.945
5.916
5.320
5.281
4.780
4.746
1.297
0.000
X : parts per Million : Proton

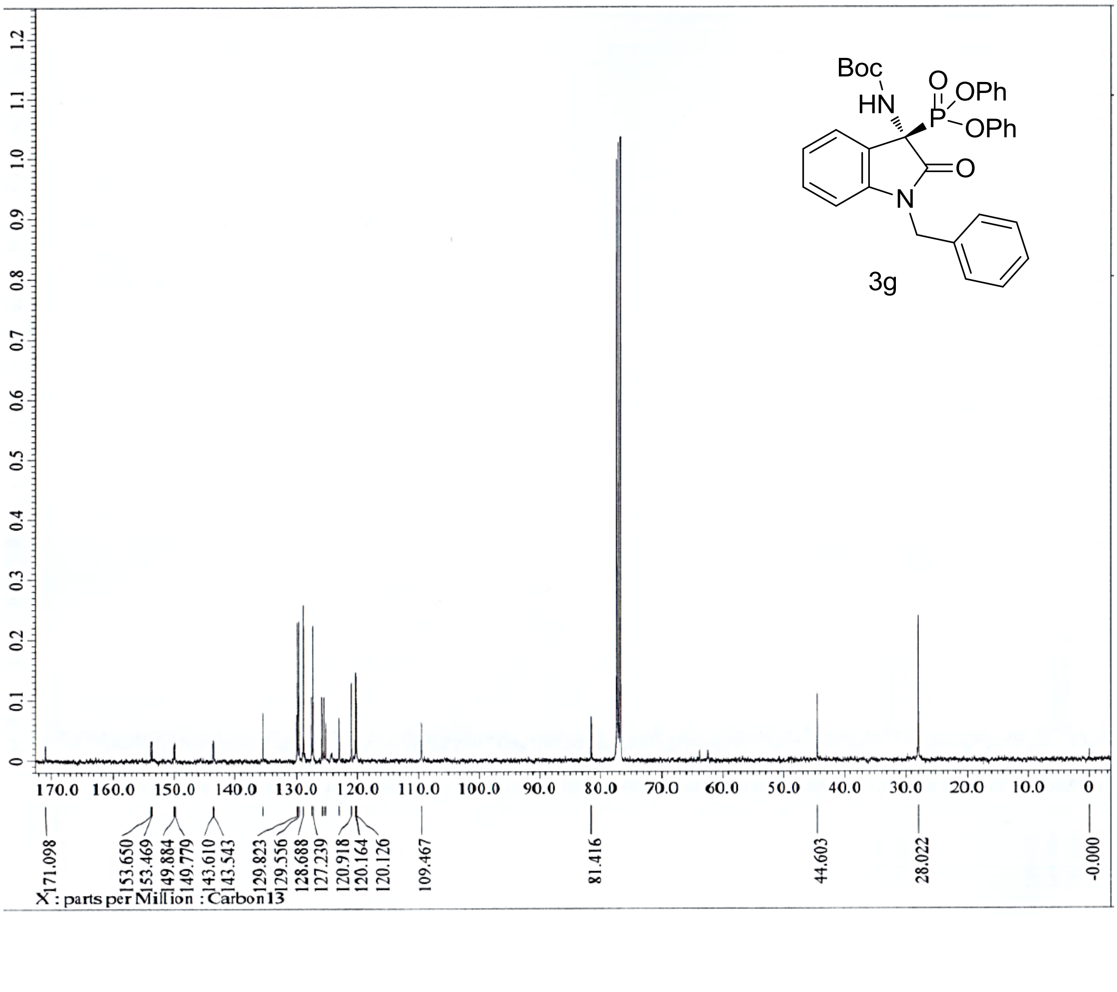
Boc
HN
O
P
OPh
OPh
O
N
3g
171.098
153.650
153.469
149.884
149.779
143.610
143.543
129.823
129.556
128.688
127.239
120.918
120.164
120.126
109.467
81.416
44.603
28.022
-0.000
X : parts per Million : Carbon13

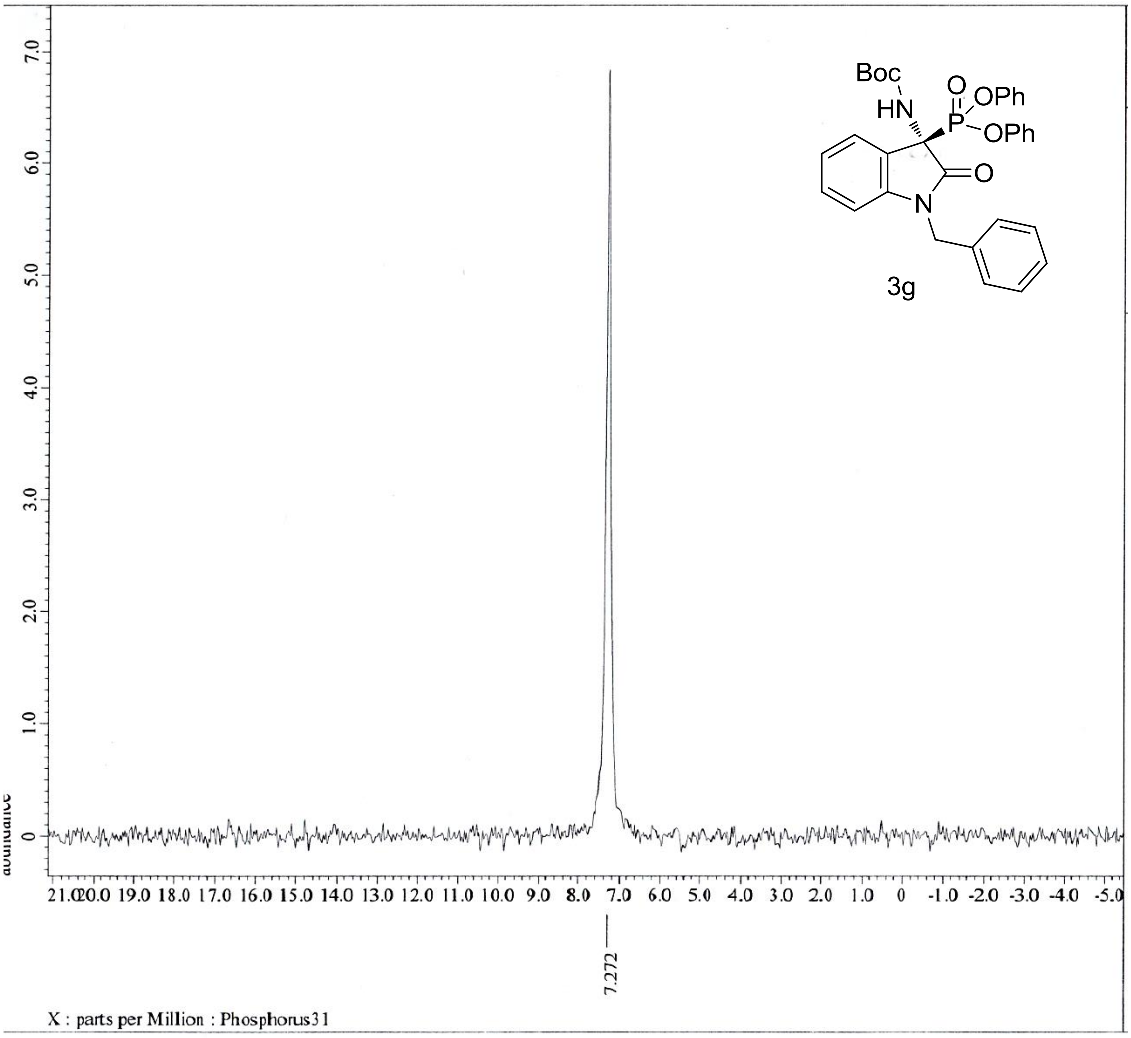
Boc
HN
O
P
OPh
OPh
O
N
3g
7.0
6.0
5.0
4.0
3.0
2.0
1.0
0
abundance
21.0 20.0 19.0 18.0 17.0 16.0 15.0 14.0 13.0 12.0 11.0 10.0 9.0 8.0 7.0 6.0 5.0 4.0 3.0 2.0 1.0 0 -1.0 -2.0 -3.0 -4.0 -5.0
7.272
X : parts per Million : Phosphorus31

<Chromatogram>

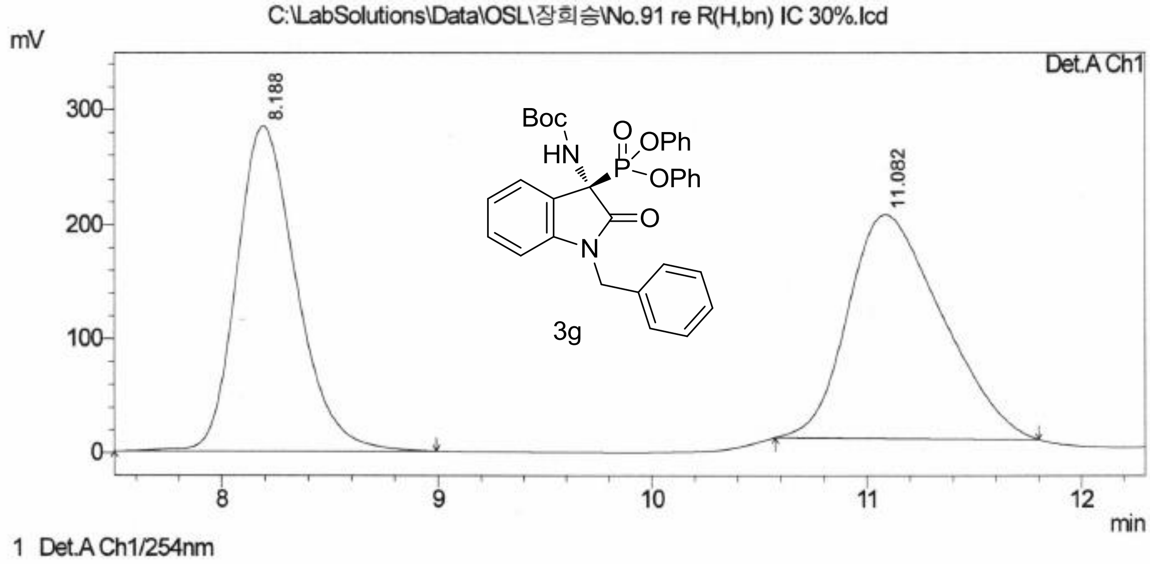

PeakTable

Detector A Ch1 254nm

Peak#	Ret. Time	Area	Height	Area %	Height %
1	8.188	5442520	284068	47.579	59.071
2	11.082	5996464	196821	52.421	40.929
Total		11438985	480889	100.000	100.000

<Chromatogram>

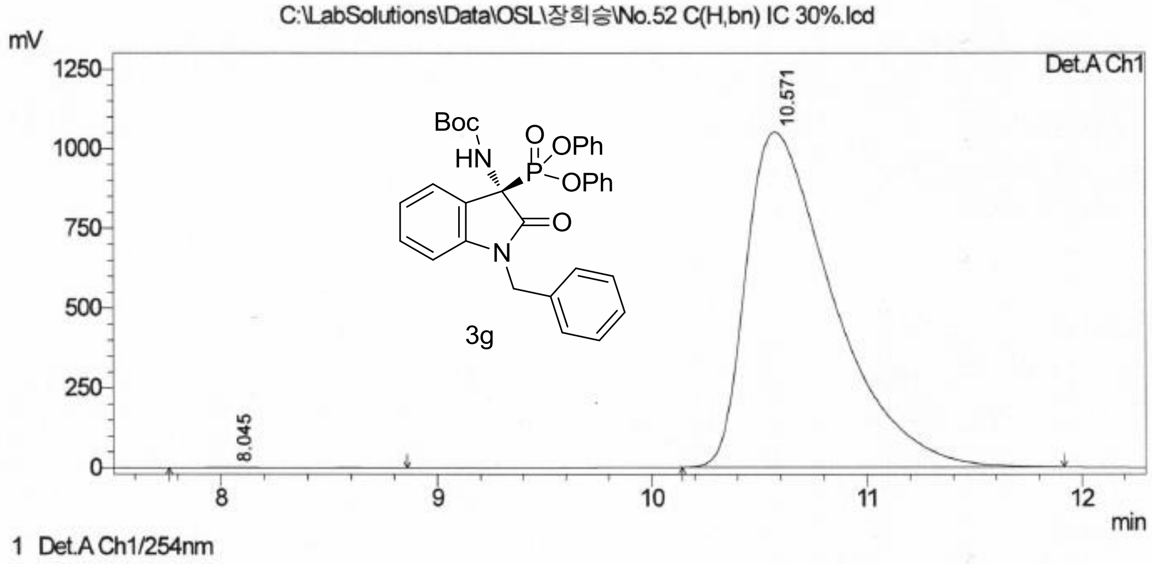

PeakTable

Detector A Ch1 254nm

Peak#	Ret. Time	Area	Height	Area %	Height %
1	8.045	30260	1005	0.103	0.096
2	10.571	29425567	1049787	99.897	99.904
Total		29455827	1050792	100.000	100.000

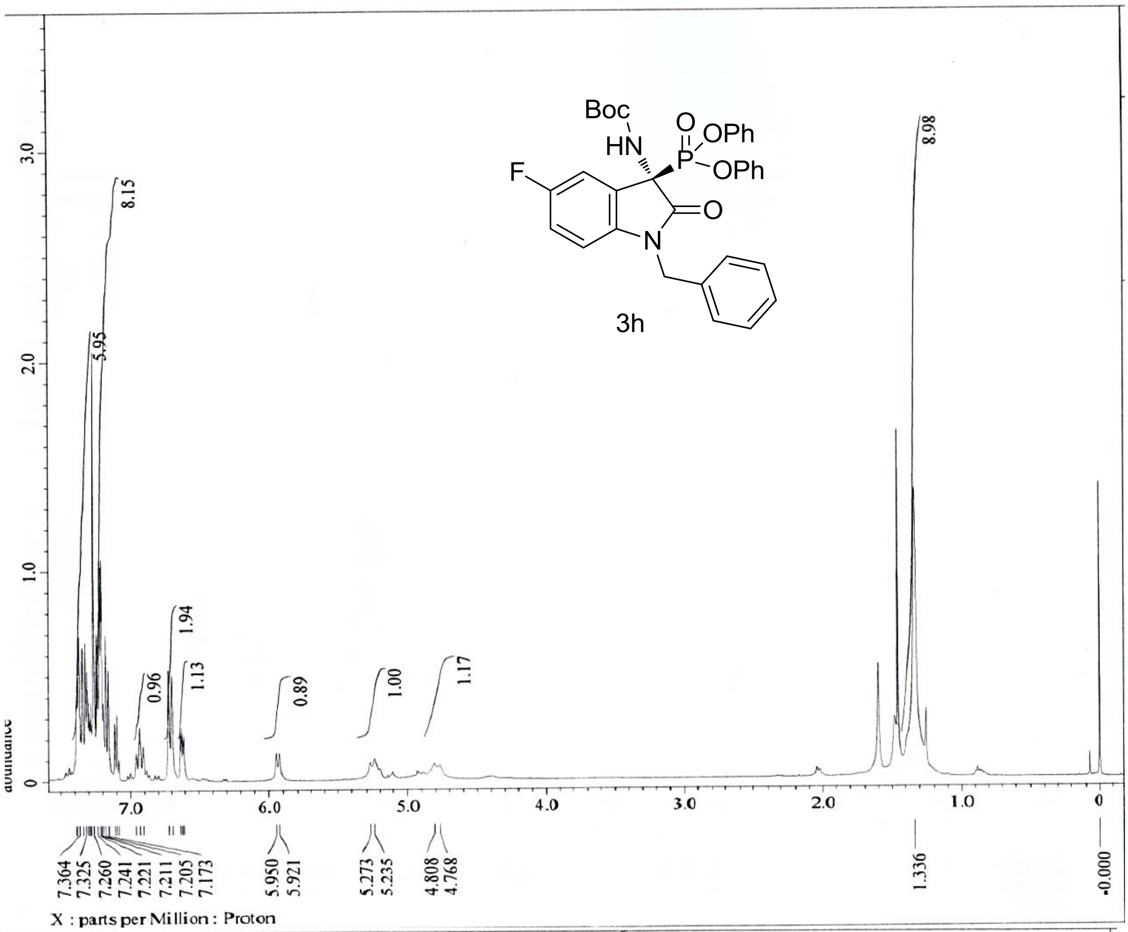
Boc
HN
O
OPh
P
OPh
F
O
N
3h
8.15
5.95
0.96
1.94
1.13
0.89
1.00
1.17
8.98
7.0
6.0
5.0
4.0
3.0
2.0
1.0
0
7.364
7.325
7.260
7.241
7.221
7.211
7.205
7.173
5.950
5.921
5.273
5.235
4.808
4.768
1.336
-0.000
X : parts per Million : Proton

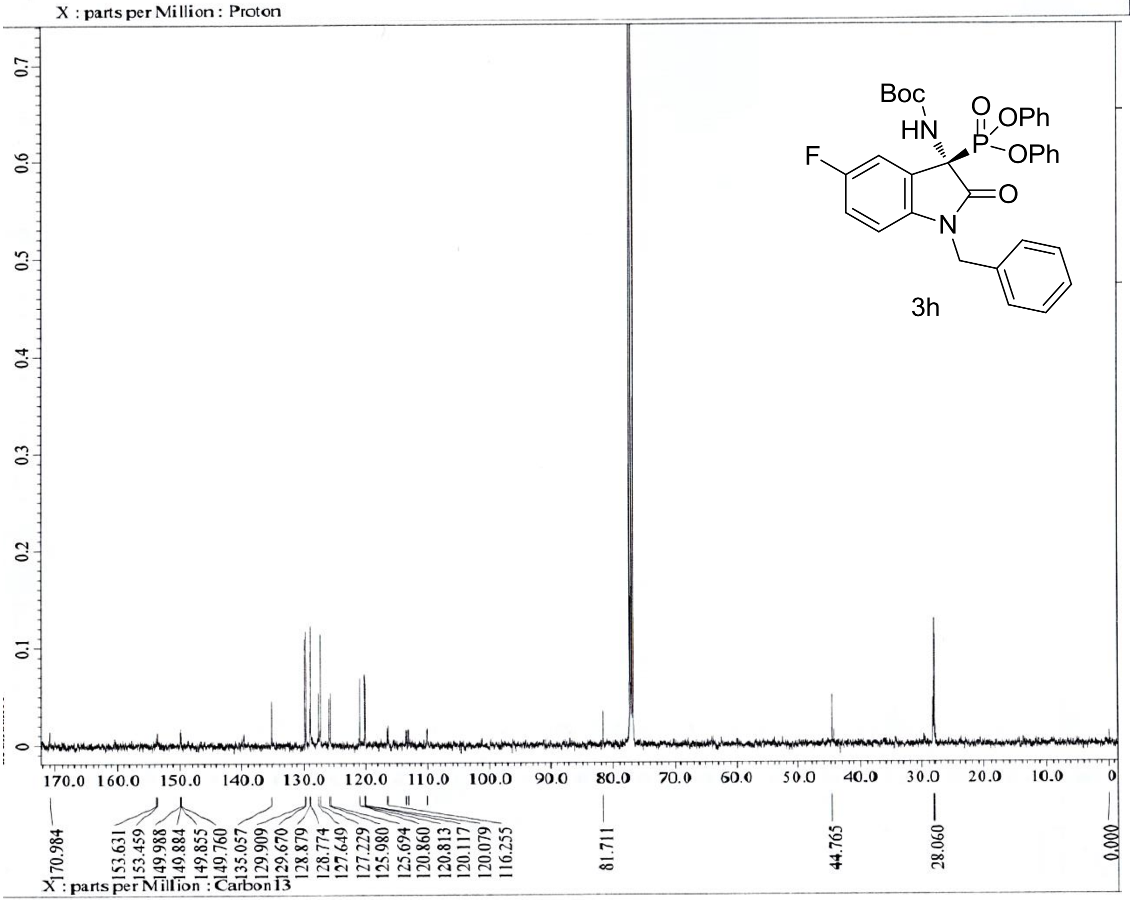
Boc
HN
O
OPh
P
OPh
F
O
N
3h
170.0
160.0
150.0
140.0
130.0
120.0
110.0
100.0
90.0
80.0
70.0
60.0
50.0
40.0
30.0
20.0
10.0
0
170.984
153.631
153.459
149.988
149.884
149.855
149.760
135.057
129.909
129.670
128.879
128.774
127.649
127.229
125.980
125.694
120.860
120.813
120.117
120.079
116.255
81.711
44.765
28.060
0.000
X : parts per Million : Carbon13

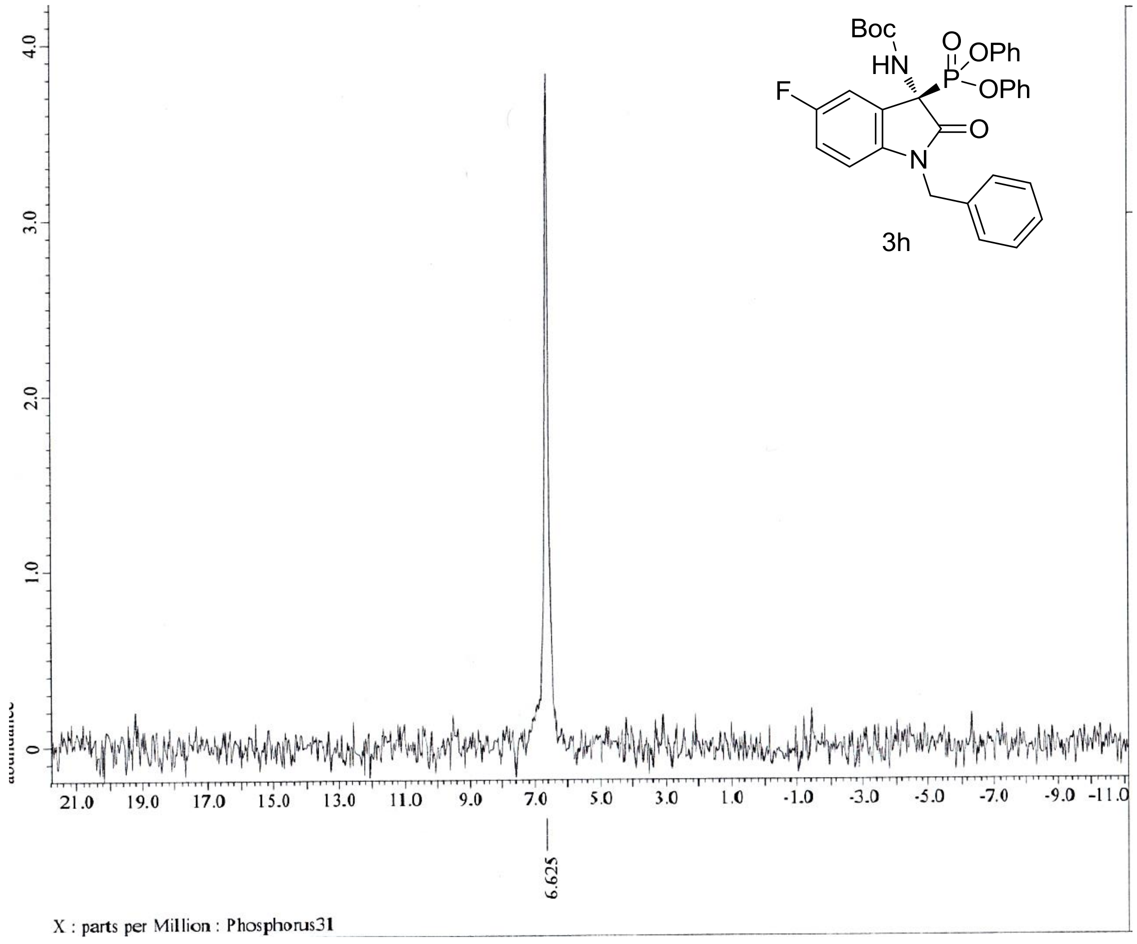
Boc
HN
O
OPh
P
OPh
F
O
N
3h
4.0
3.0
2.0
1.0
0
abundance
21.0
19.0
17.0
15.0
13.0
11.0
9.0
7.0
5.0
3.0
1.0
-1.0
-3.0
-5.0
-7.0
-9.0
-11.0
6.625
X : parts per Million : Phosphorus31

크로마토그램

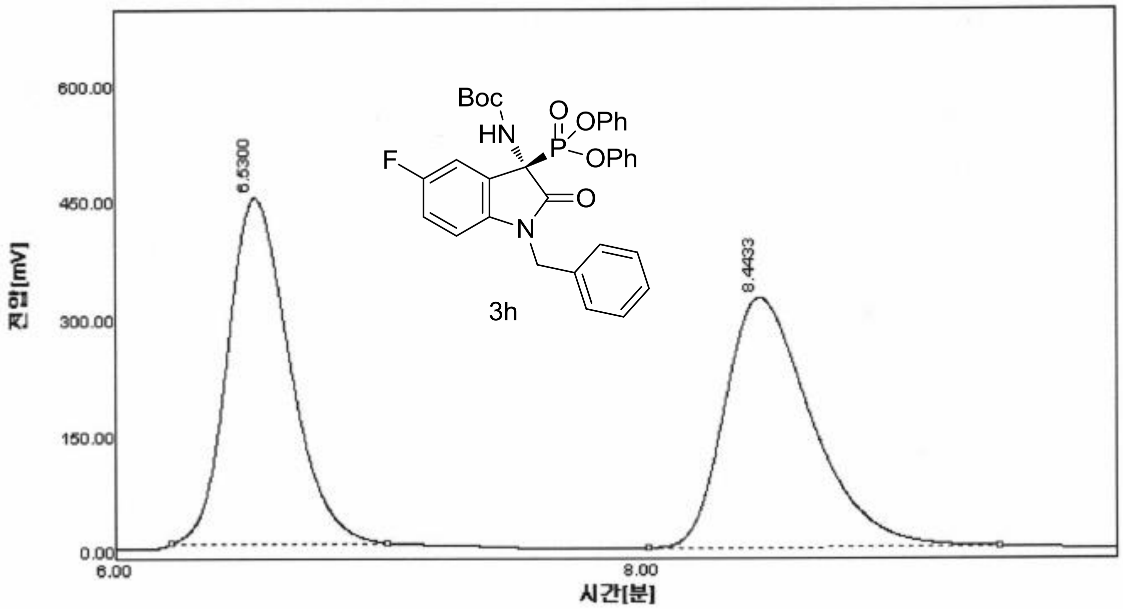

적분 결과

번호	RT[분]	면적비[%]	면적[mV*s]	폭[초]	형태
1	6.5300	49.64	7226.5016	25.29	FF
2	8.4433	50.36	7330.8398	32.61	FF
합계			14557.3414		

크로마토그램

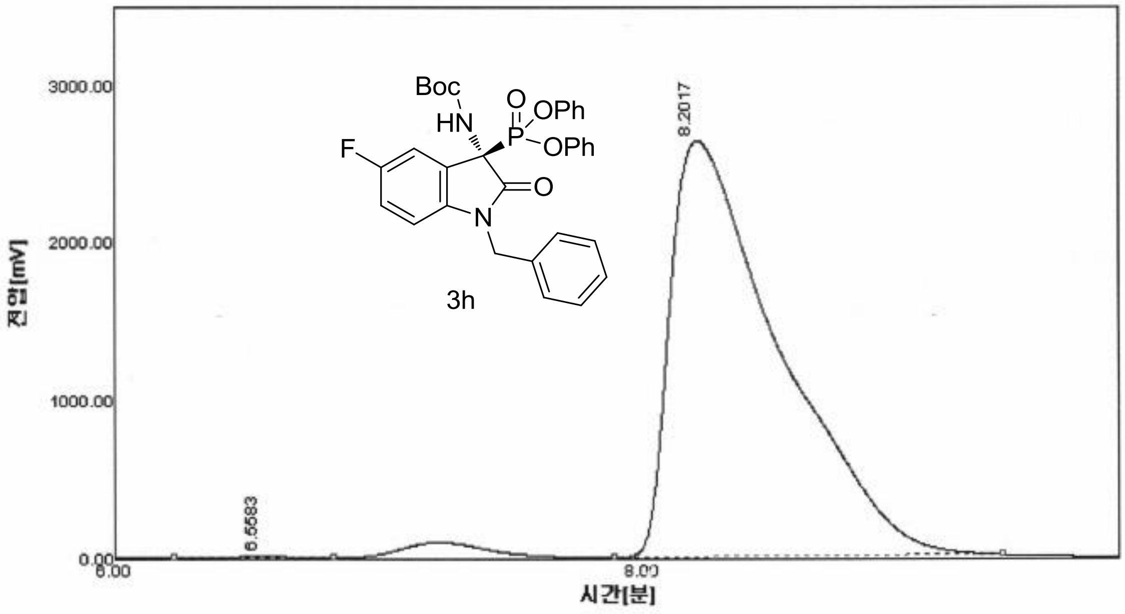

적분 결과

번호	RT[분]	면적비[%]	면적[mV*s]	폭[초]	형태
1	6.5583	0.23	172.1638	23.93	FF
2	8.2017	99.77	2643.9487	58.09	FF
합계			2816.1125		

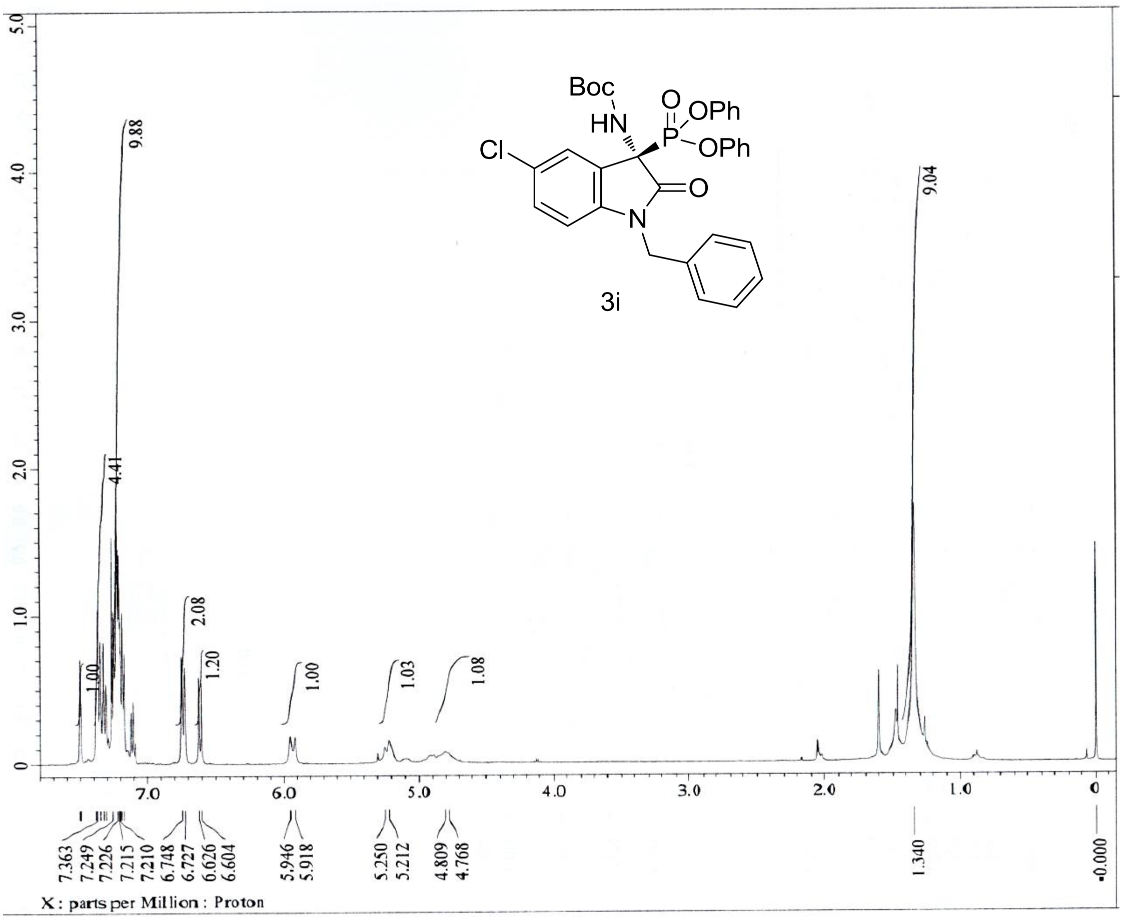
Boc
HN
O
OPh
P
OPh
Cl
O
N
3i
9.88
4.41
2.08
1.20
1.00
1.00
1.03
1.08
9.04
7.363
7.249
7.226
7.215
7.210
6.748
6.727
6.626
6.604
5.946
5.918
5.250
5.212
4.809
4.768
1.340
-0.000
X : parts per Million : Proton

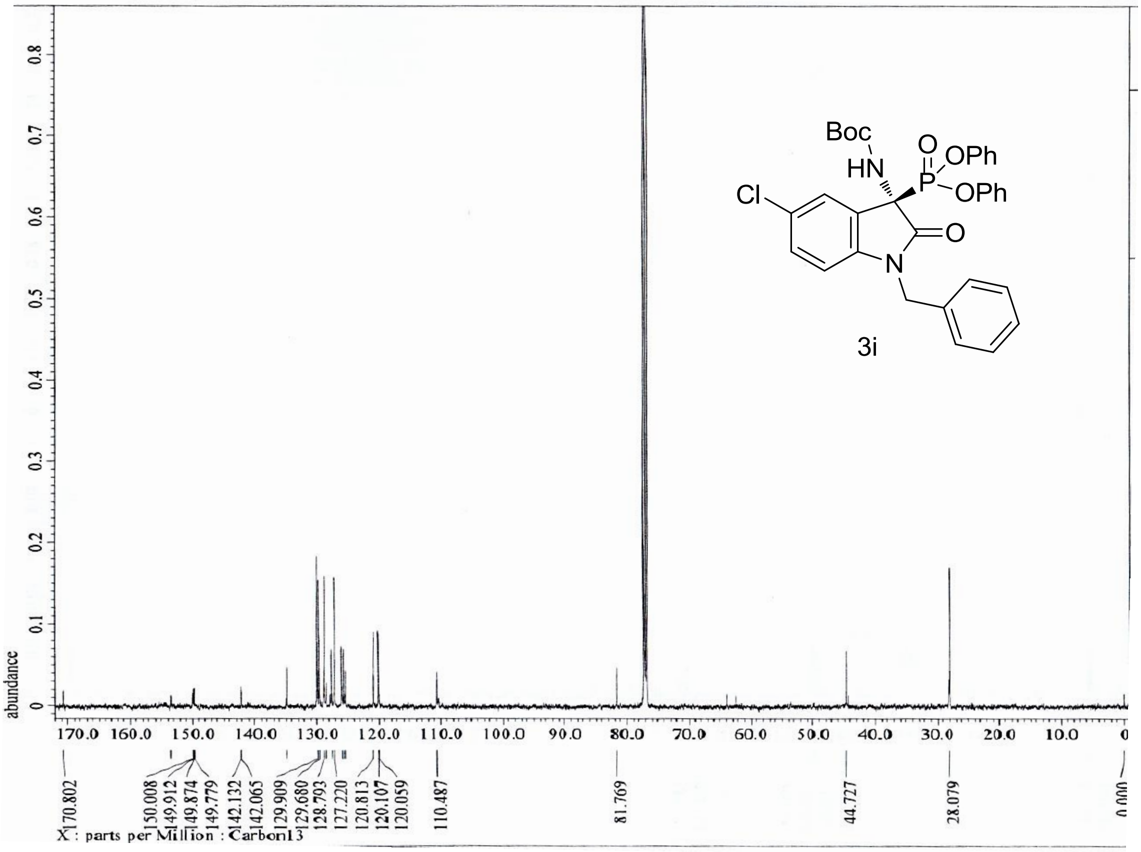
Boc
HN
O
OPh
P
OPh
Cl
O
N
3i
abundance
170.802
150.008
149.912
149.874
149.779
142.132
142.065
129.909
129.680
128.793
127.220
120.813
120.107
120.059
110.487
81.769
44.727
28.079
0.000
X : parts per Million : Carbon13

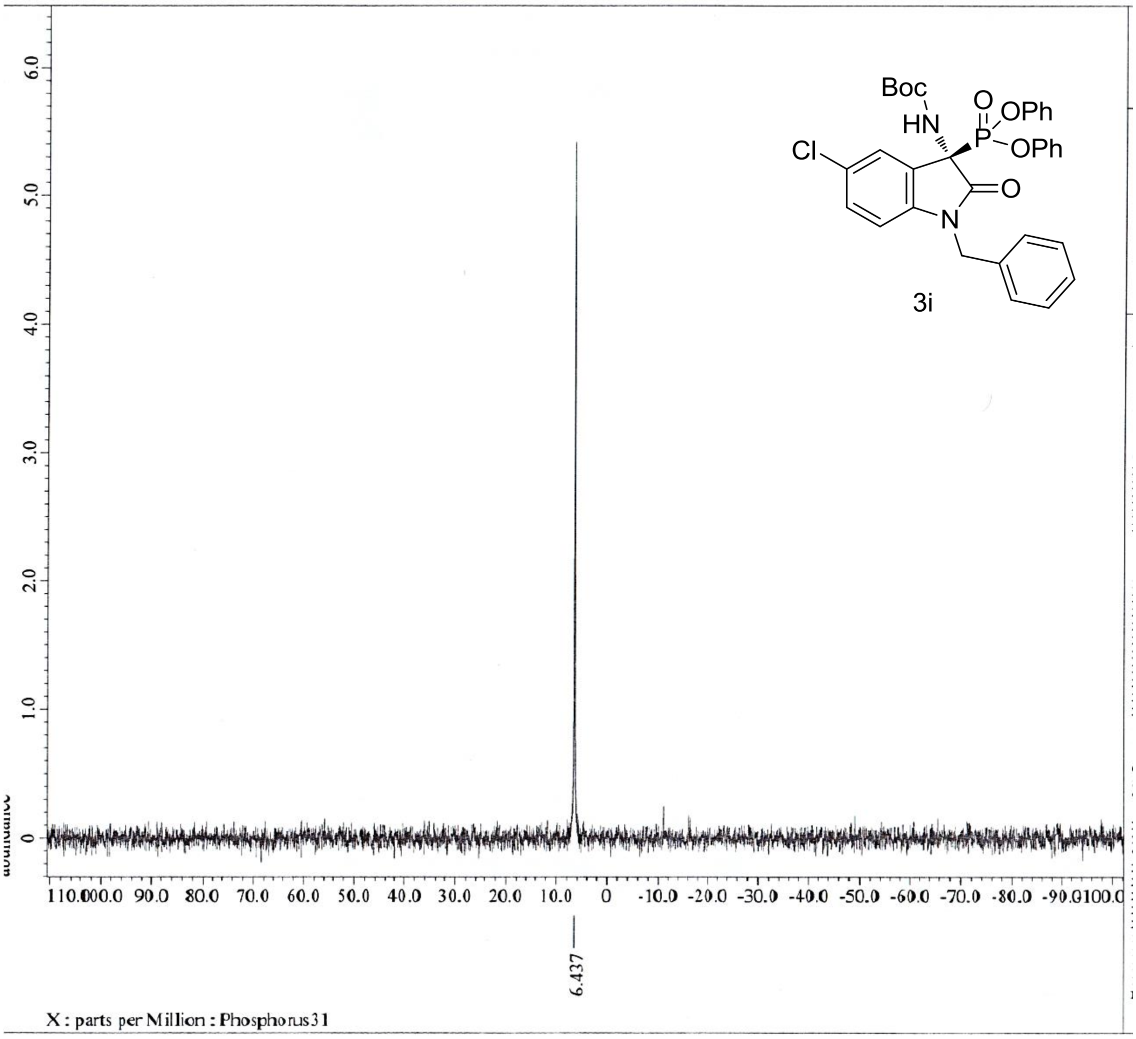

Boc
HN
O
OPh
P
OPh
Cl
O
N
3i
6.0
5.0
4.0
3.0
2.0
1.0
0
110.0100.0 90.0 80.0 70.0 60.0 50.0 40.0 30.0 20.0 10.0 0 -10.0 -20.0 -30.0 -40.0 -50.0 -60.0 -70.0 -80.0 -90.0100.0
6.437
X : parts per Million : Phosphorus31

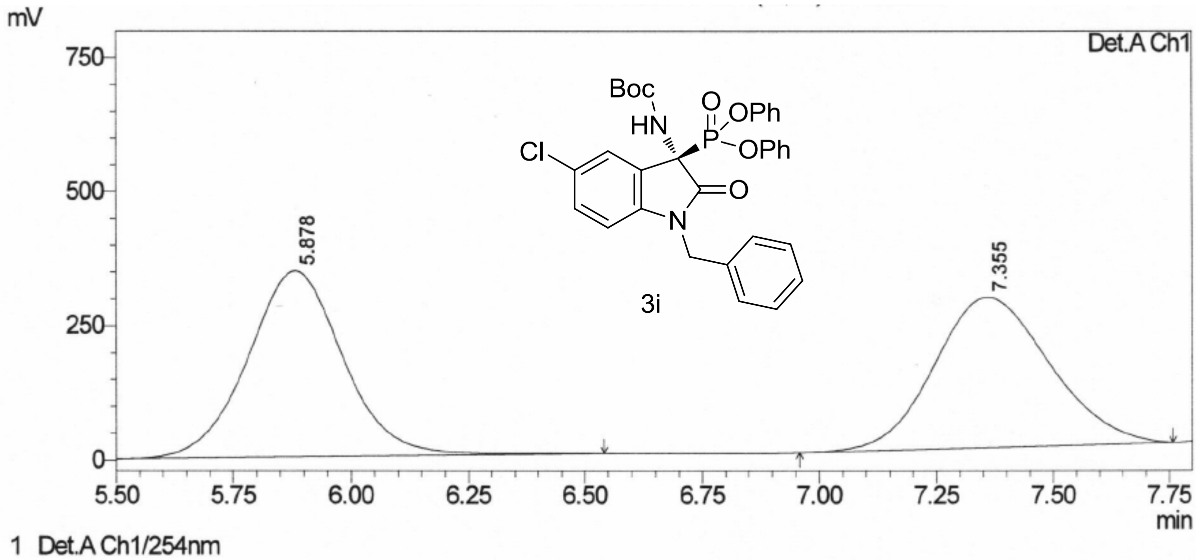

1 Det.A Ch1/254nm

PeakTable

Detector A Ch1 254nm

Peak#	Ret. Time	Area	Height	Area %	Height %
1	5.878	4868166	346176	50.302	55.263
2	7.355	4809797	280235	49.698	44.737
Total		9677964	626411	100.000	100.000

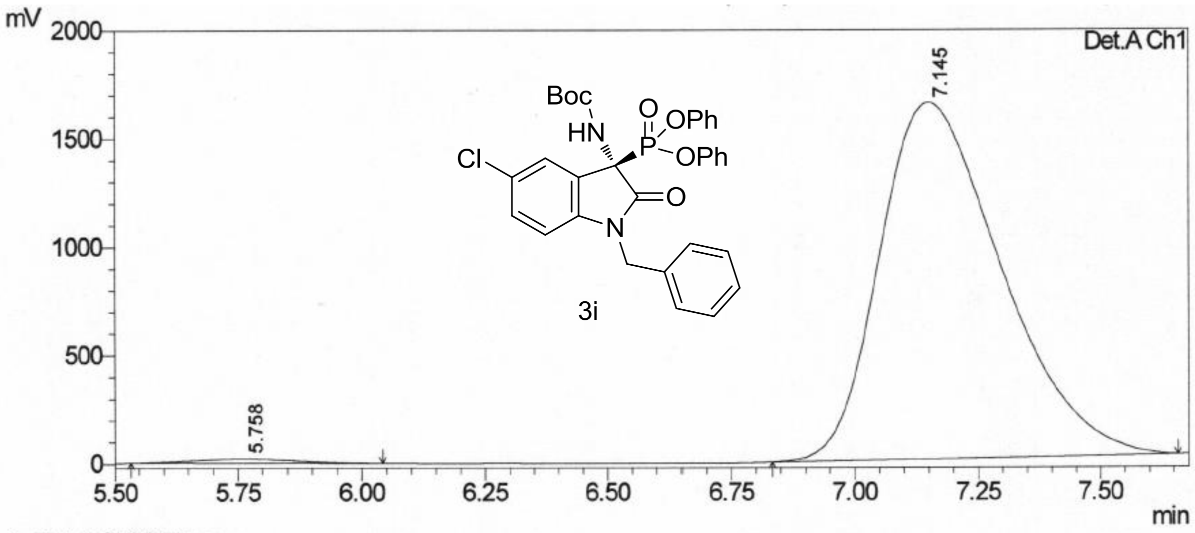

1 Det.A Ch1/254nm

PeakTable

Detector A Ch1 254nm

Peak#	Ret. Time	Area	Height	Area %	Height %
1	5.758	261958	18236	0.920	1.095
2	7.145	28213493	1646431	99.080	98.905
Total		28475450	1664667	100.000	100.000

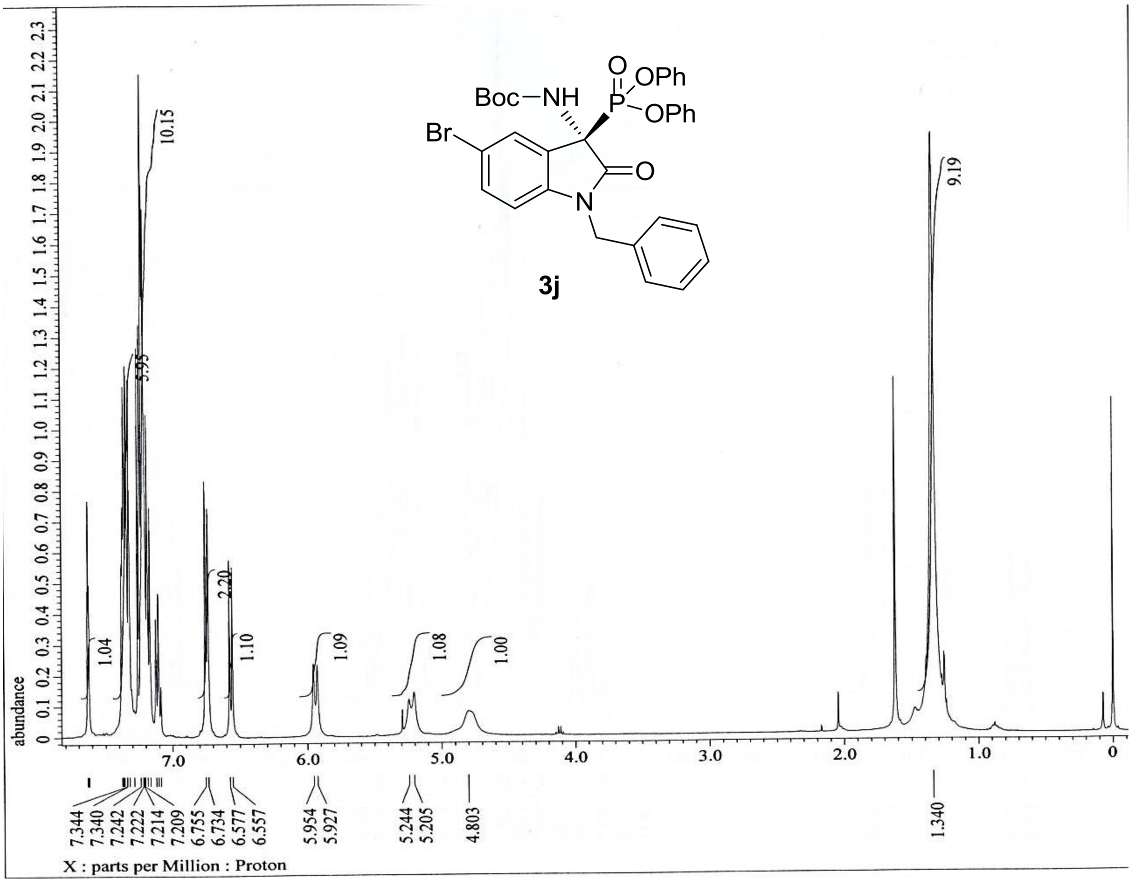
Boc—NH
OPh
OPh
Br
3j
10.15
5.95
2.20
1.04
1.10
1.09
1.08
1.00
9.19
abundance
7.344
7.340
7.242
7.222
7.214
7.209
6.755
6.734
6.577
6.557
5.954
5.927
5.244
5.205
4.803
1.340
X : parts per Million : Proton

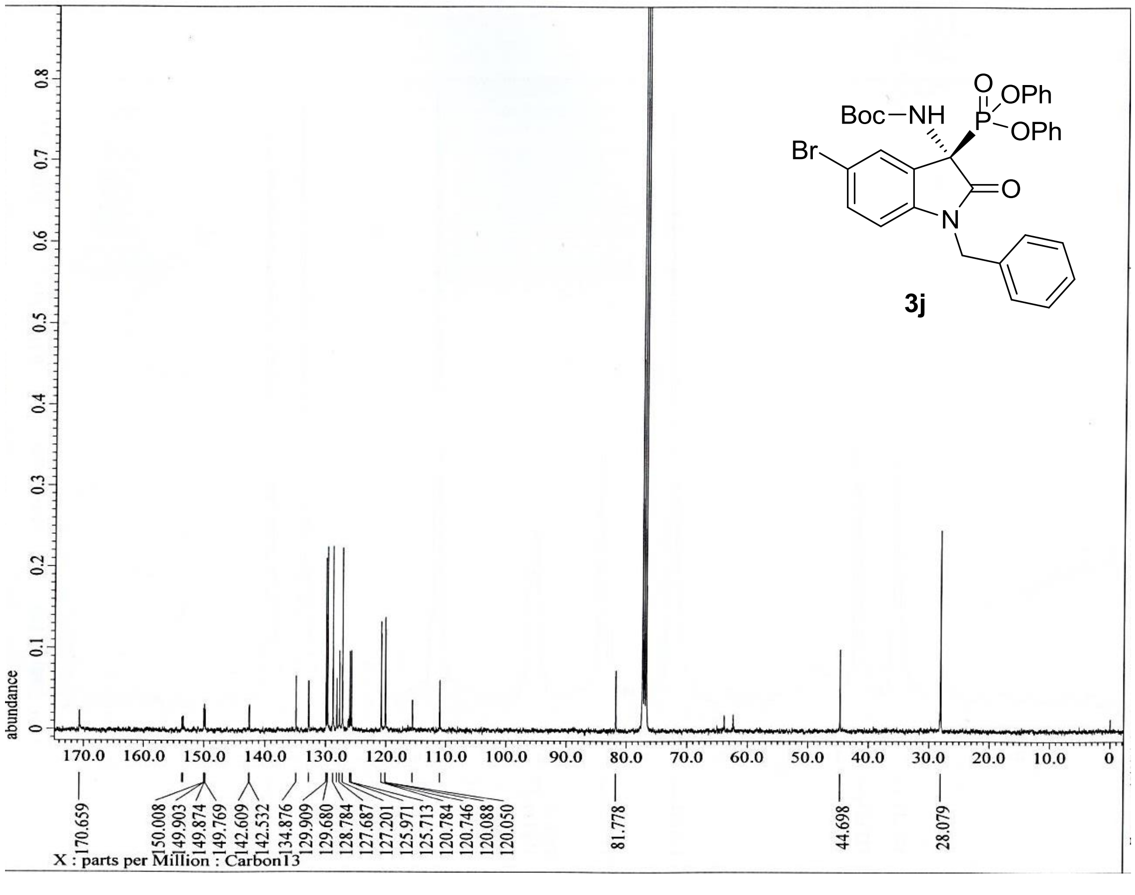
Boc—NH
OPh
OPh
Br
3j
abundance
170.659
150.008
149.903
149.874
149.769
142.609
142.532
134.876
129.909
129.680
128.784
127.687
127.201
125.971
125.713
120.784
120.746
120.088
120.050
81.778
44.698
28.079
X : parts per Million : Carbon13

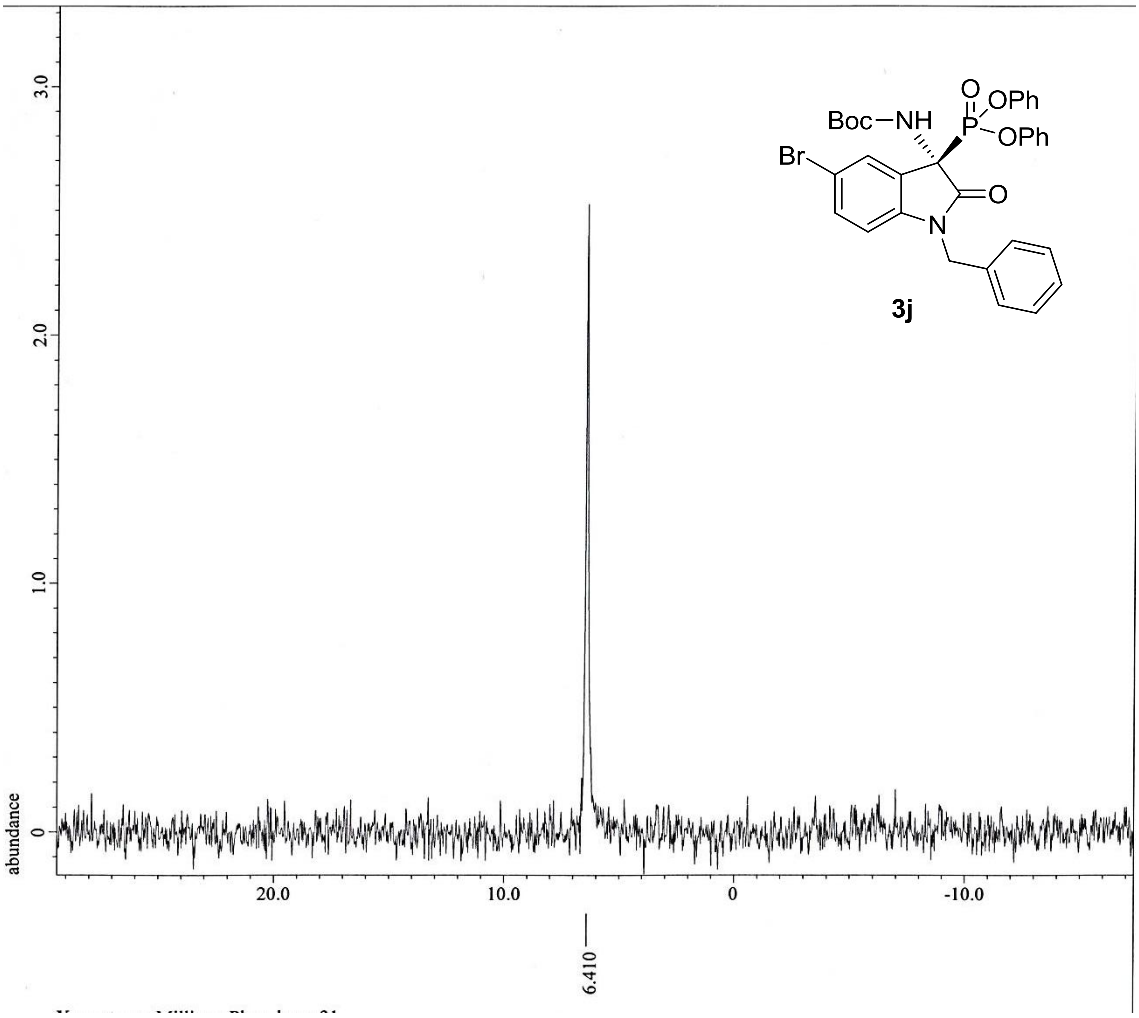
Boc—NH
O
P
OPh
OPh
Br
N
O
3j
abundance
0
1.0
2.0
3.0
20.0
10.0
0
-10.0
6.410

크로마토그램

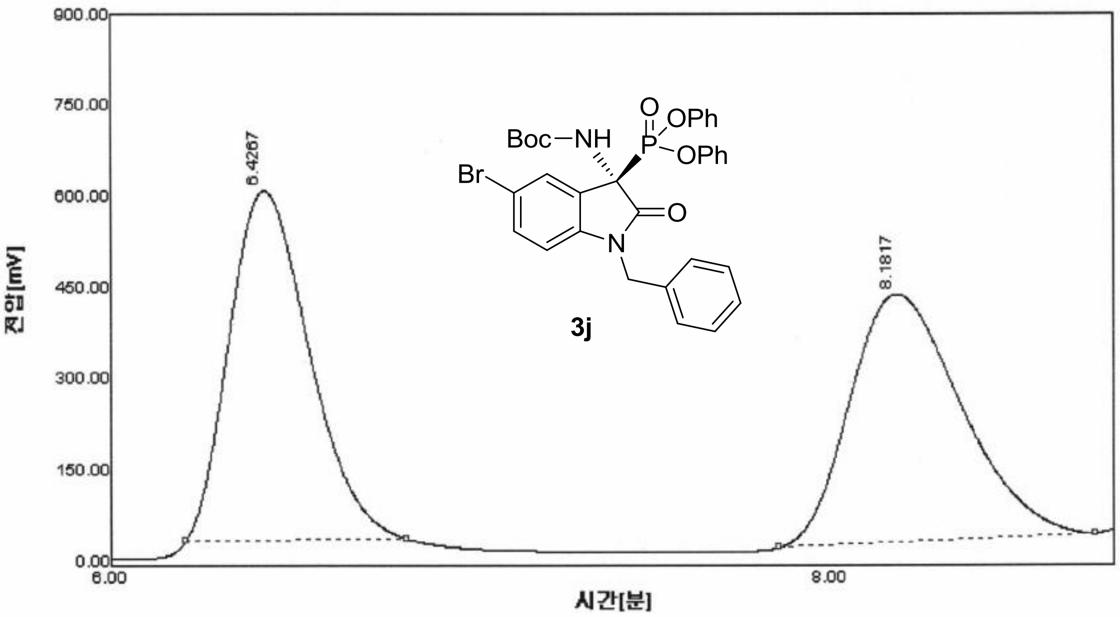

적분 결과

번호	RT[분]	면적비[%]	면적[mV*s]	폭[초]	형태
1	6.4267	50.26	8794.6422	24.45	FF
2	8.1817	49.74	8703.8578	31.17	FF
합계			17498.5000		

크로마토그램

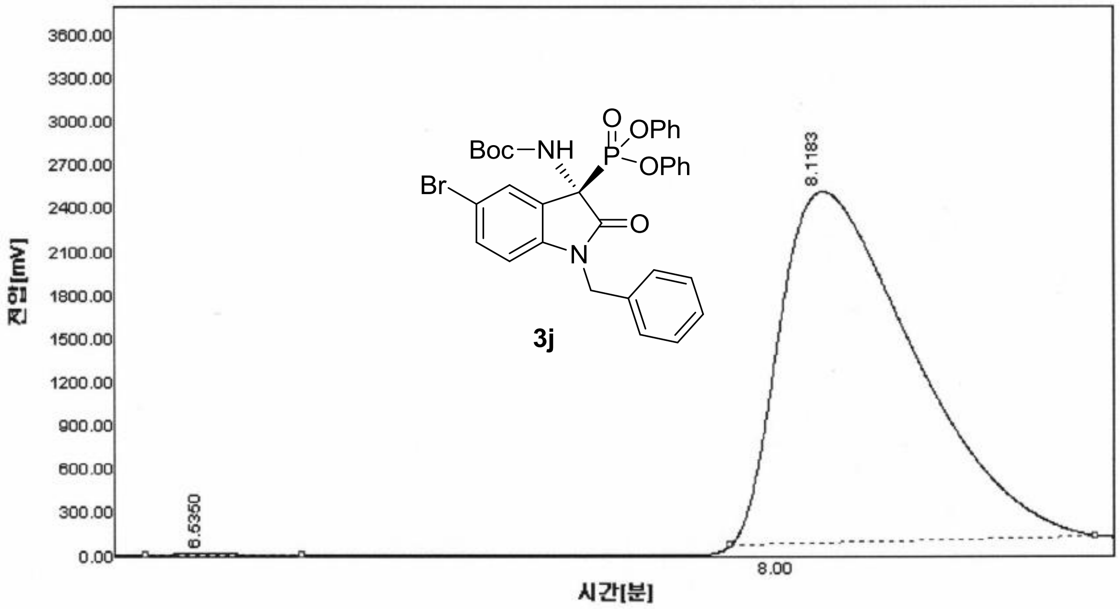

적분 결과

번호	RT[분]	면적비[%]	면적[mV*s]	폭[초]	형태
1	6.5350	0.23	131.4502	21.01	FF
2	8.1183	99.77	58057.2375	34.22	FF
합계			58188.6877		

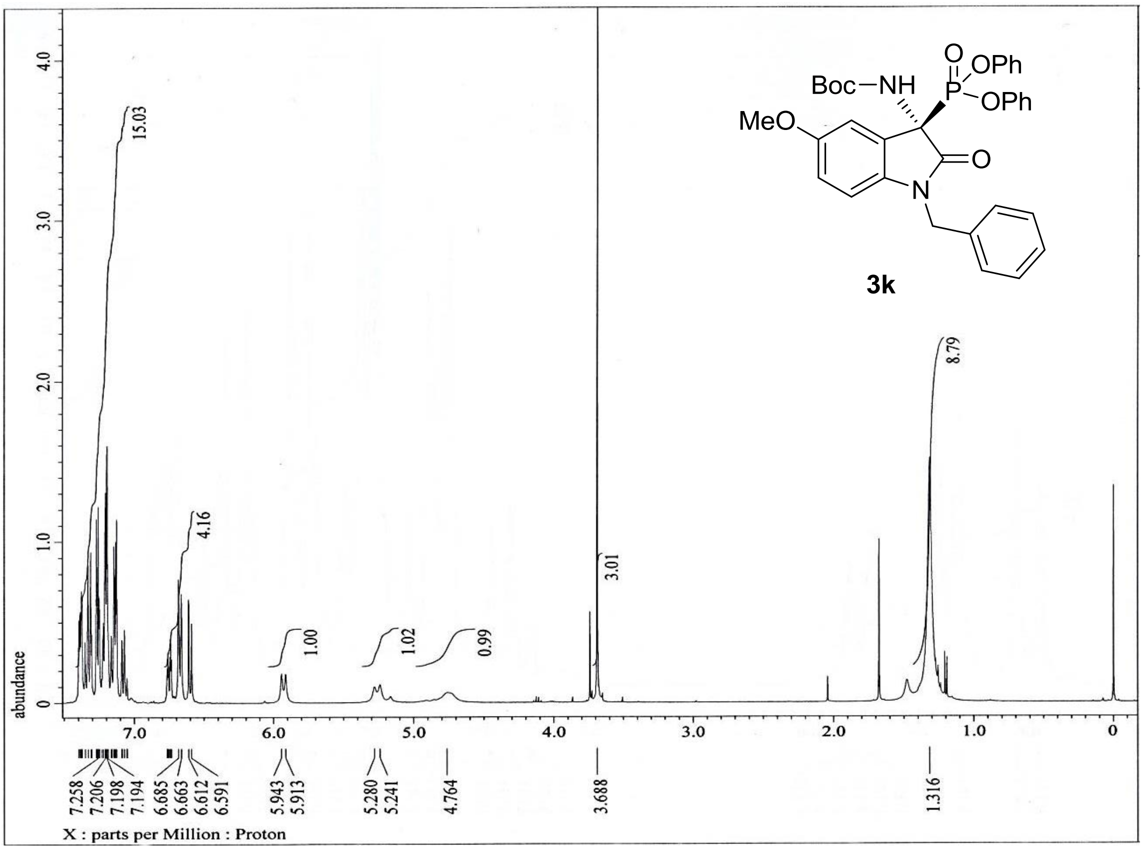
Boc—NH
O
OPh
P
OPh
MeO
O
N
3k
15.03
4.16
1.00
1.02
0.99
3.01
8.79
abundance
7.258
7.206
7.198
7.194
6.685
6.663
6.612
6.591
5.943
5.913
5.280
5.241
4.764
3.688
1.316
X : parts per Million : Proton

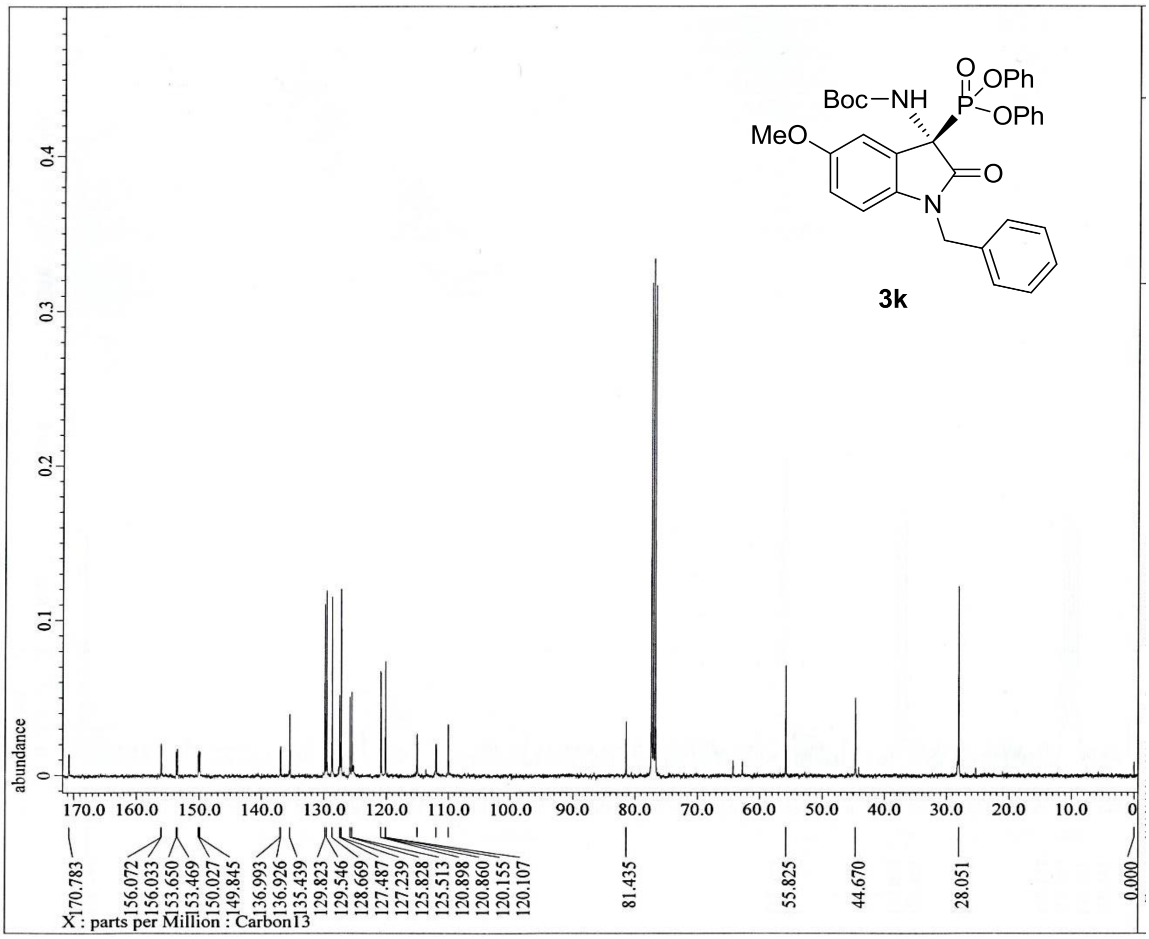
Boc—NH
O
OPh
P
OPh
MeO
O
N
3k
abundance
170.783
156.072
156.033
153.650
153.469
150.027
149.845
136.993
136.926
135.439
129.823
129.546
128.669
127.487
127.239
125.828
125.513
120.898
120.860
120.155
120.107
81.435
55.825
44.670
28.051
0.000
X : parts per Million : Carbon13

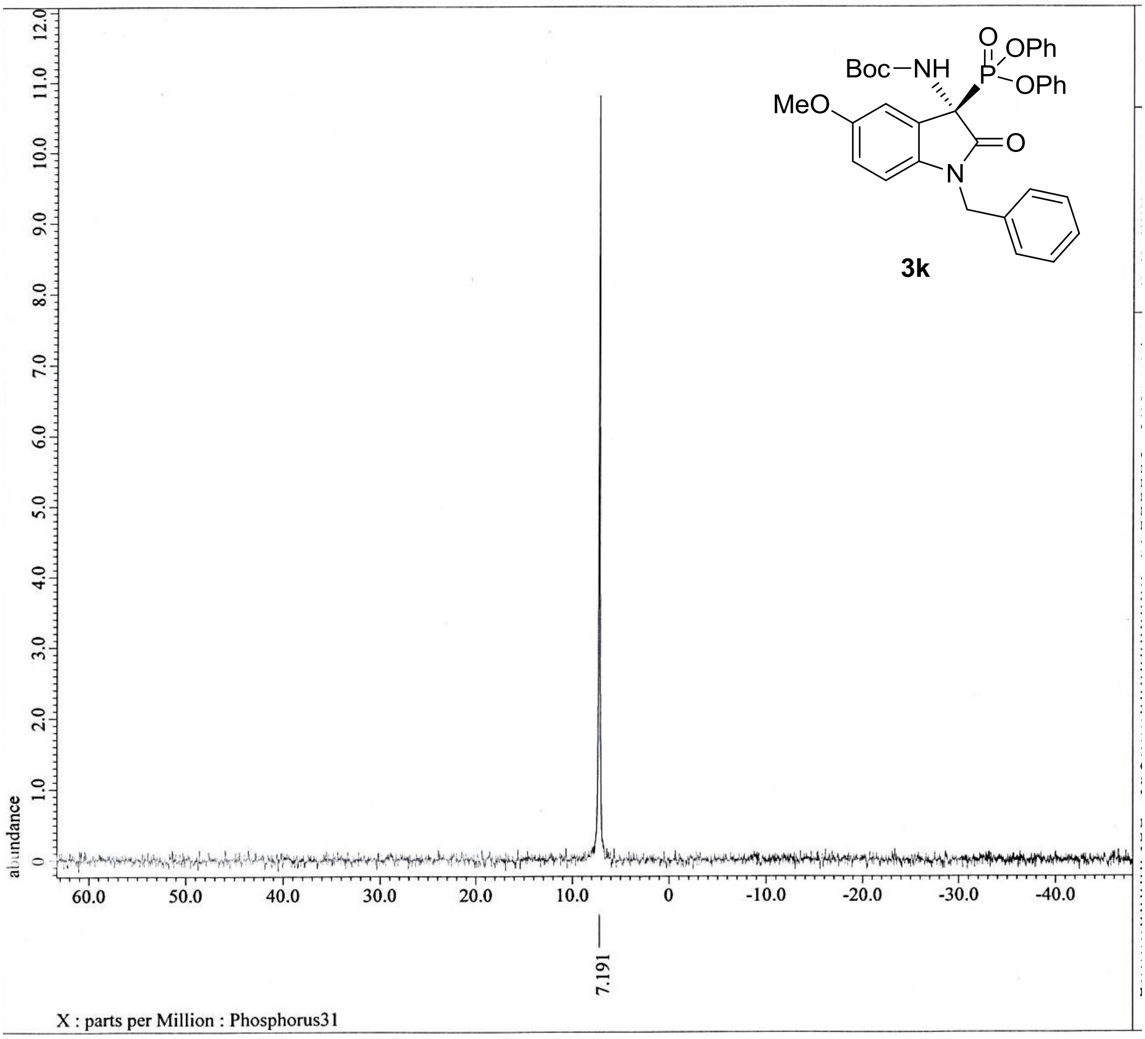
Boc—NH
OPh
OPh
MeO
O
N
3k
abundance
60.0
50.0
40.0
30.0
20.0
10.0
0
-10.0
-20.0
-30.0
-40.0
7.191
X : parts per Million : Phosphorus31

크로마토그램

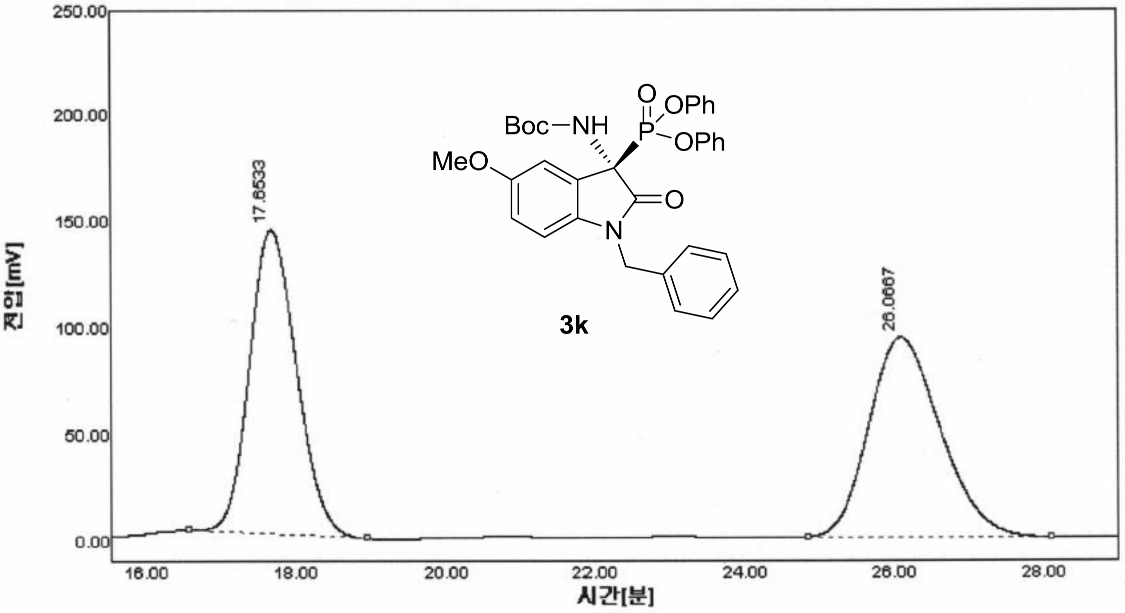

적분 결과

번호	RT[분]	면적비[%]	면적[mV*s]	폭[초]	형태
1	17.6533	49.95	6188.3461	68.90	FF
2	26.0667	50.05	6201.3781	105.46	FF
합계			12389.7242		

크로마토그램

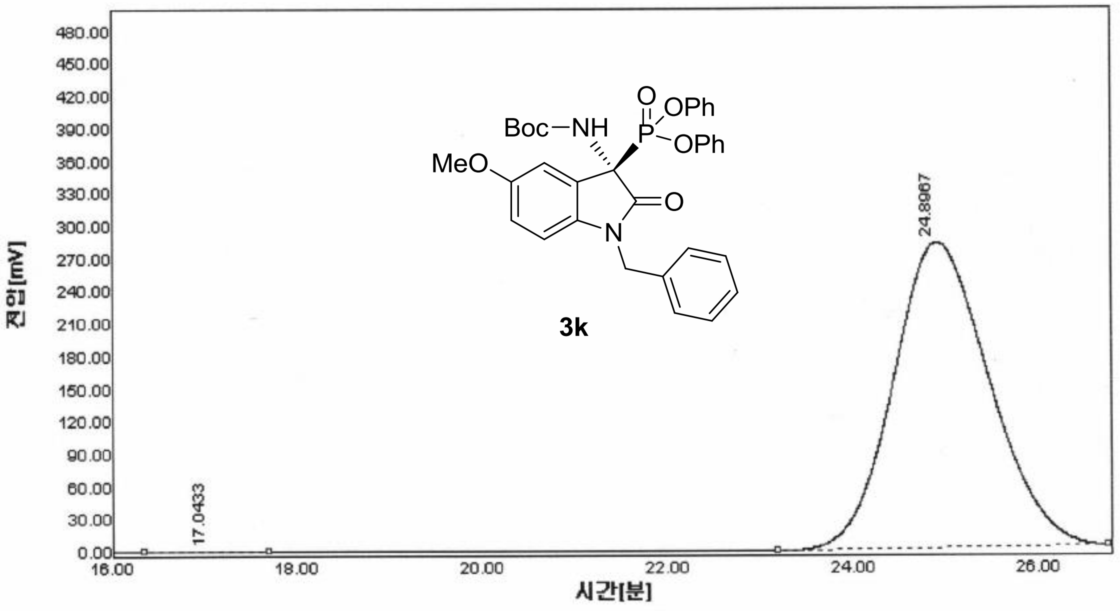

적분 결과

번호	RT[분]	면적비[%]	면적[mV*s]	폭[초]	형태
1	17.0433	0.22	45.3051	61.73	FF
2	24.8967	99.78	20232.5344	115.18	BB
합계			20277.8395		

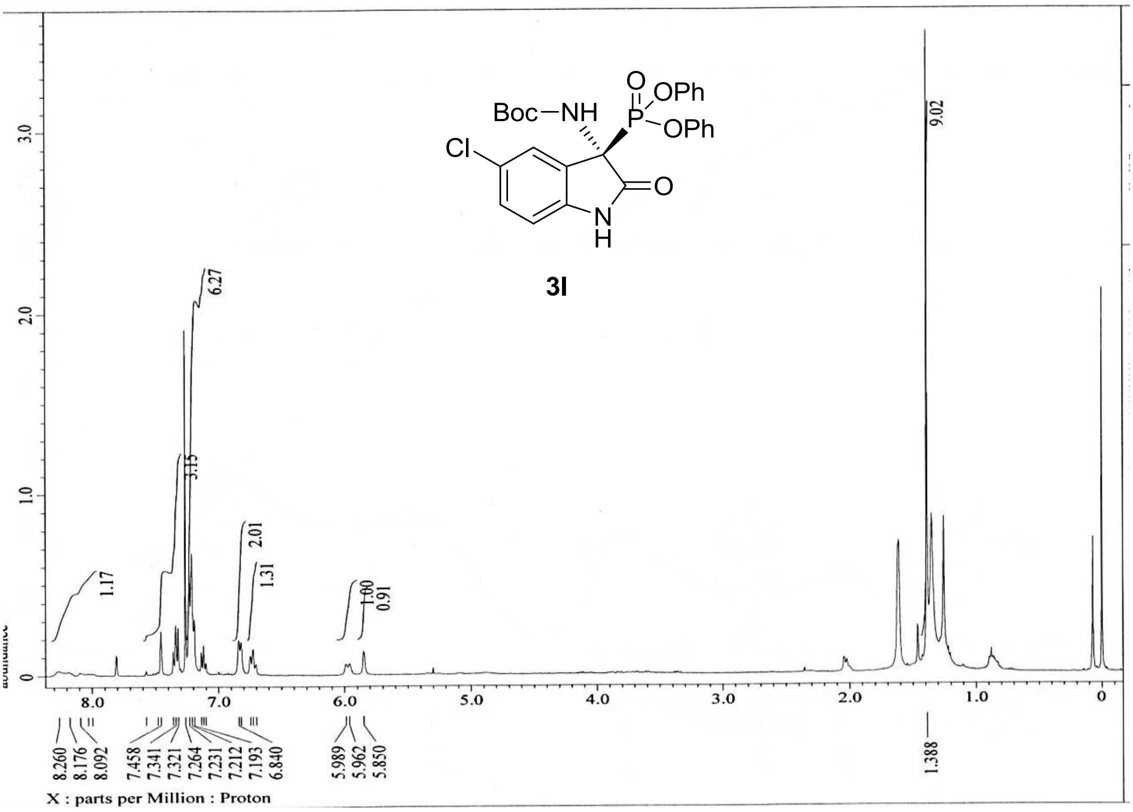
Boc—NH
OPh
OPh
Cl
O
N
H
3l
9.02
6.27
3.15
2.01
1.31
1.17
1.00
0.91
8.260
8.176
8.092
7.458
7.341
7.321
7.264
7.231
7.212
7.193
6.840
5.989
5.962
5.850
1.388
X : parts per Million : Proton

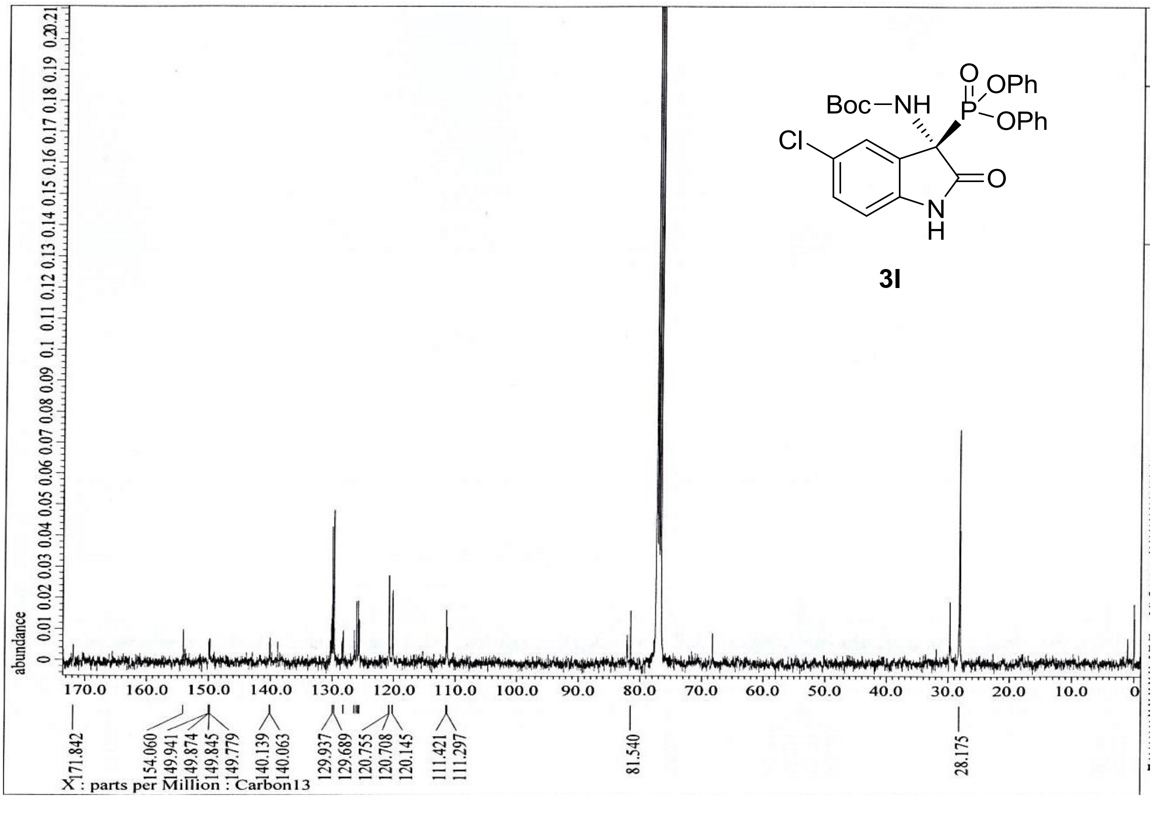
Boc—NH
OPh
OPh
Cl
O
N
H
3l
abundance
171.842
154.060
149.941
149.874
149.845
149.779
140.139
140.063
129.937
129.689
120.755
120.708
120.145
111.421
111.297
81.540
28.175
X : parts per Million : Carbon13

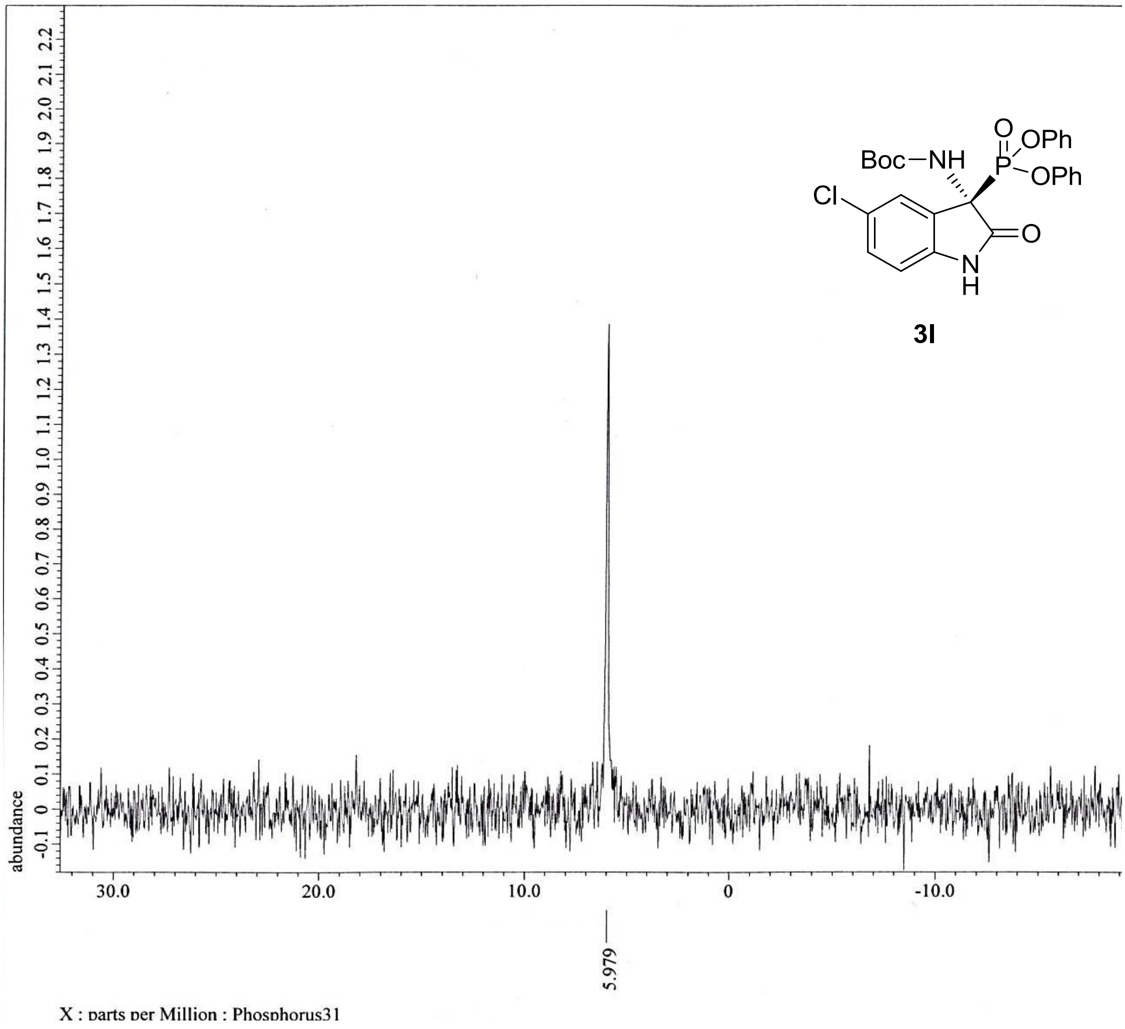
Boc—NH
OPh
OPh
Cl
O
O
N
H
3l
abundance
-0.1 0 0.1 0.2 0.3 0.4 0.5 0.6 0.7 0.8 0.9 1.0 1.1 1.2 1.3 1.4 1.5 1.6 1.7 1.8 1.9 2.0 2.1 2.2
30.0
20.0
10.0
0
-10.0
5.979
X : parts per Million : Phosphorus31

크로마토그램

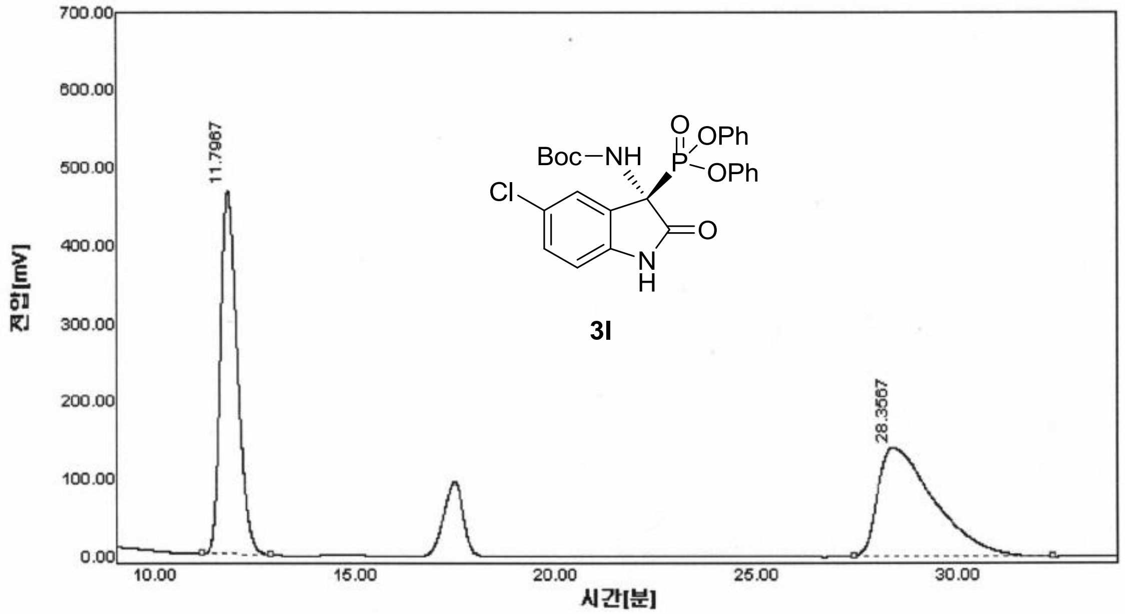

적분 결과

번호	RT[분]	면적비[%]	면적[mV*s]	폭[초]	형태
1	11.7967	50.01	13597.0187	45.74	FF
2	28.3567	49.99	13591.6969	137.88	FF
합계			27188.7156		

크로마토그램

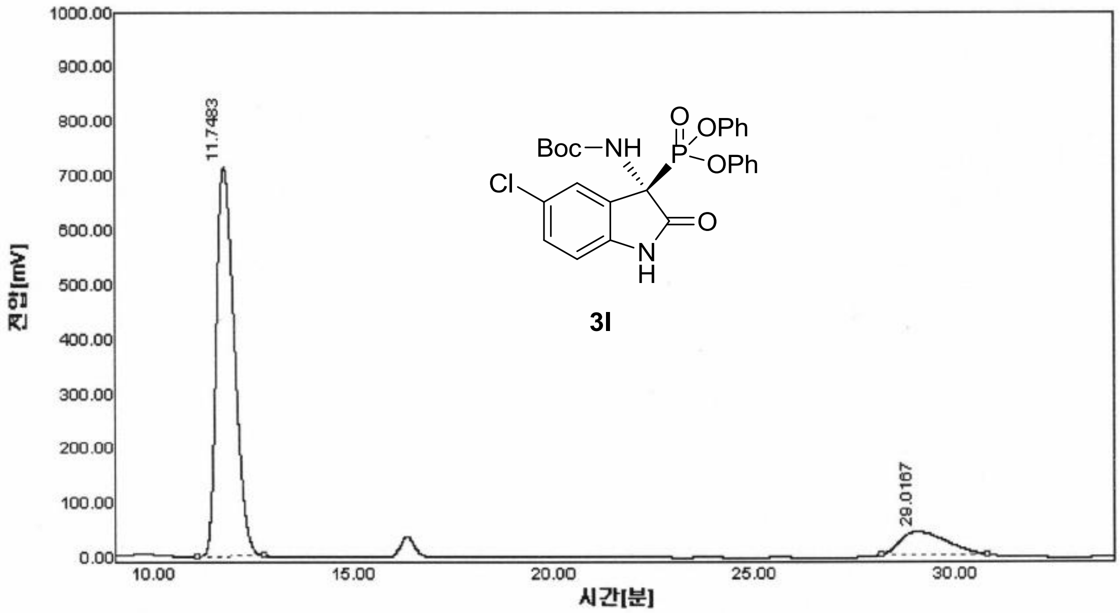

적분 결과

번호	RT[분]	면적비[%]	면적[mV*s]	폭[초]	형태
1	11.7483	86.66	22079.3812	49.21	FF
2	29.0167	13.34	3397.9391	130.30	FF
합계			25477.3203		

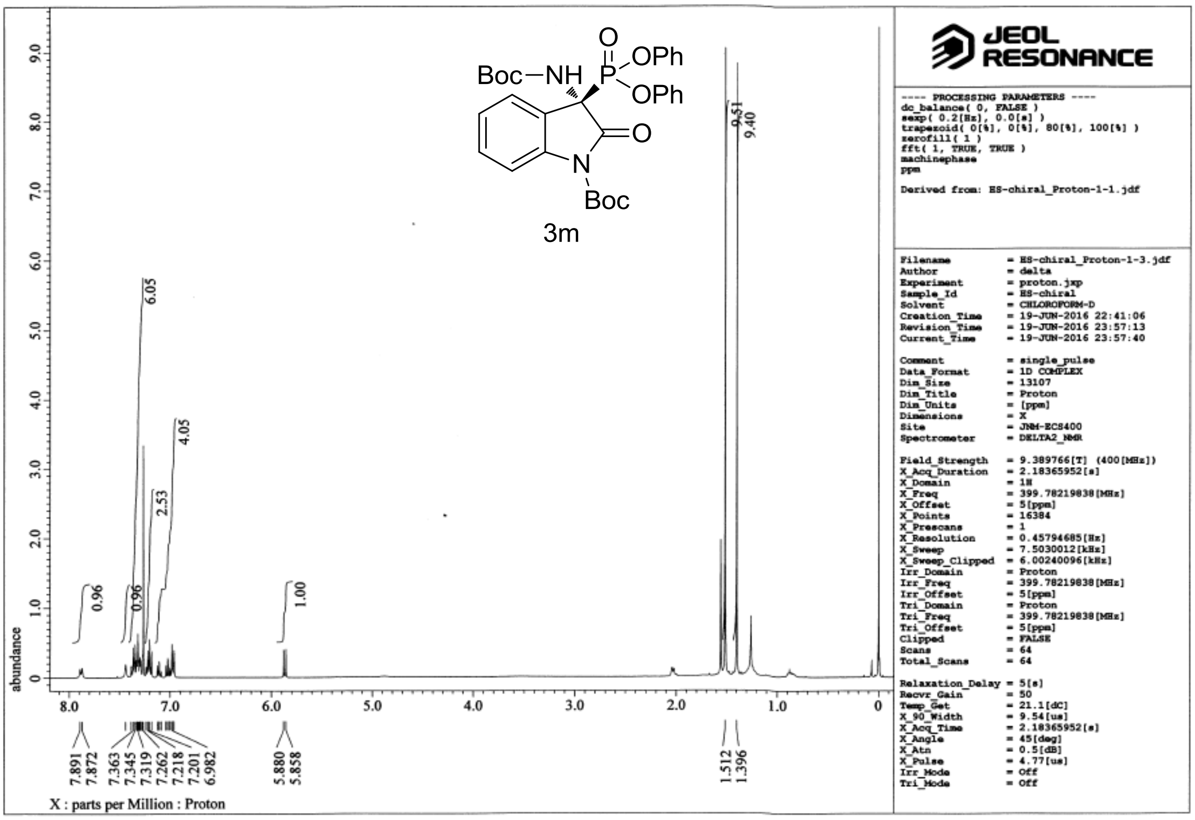

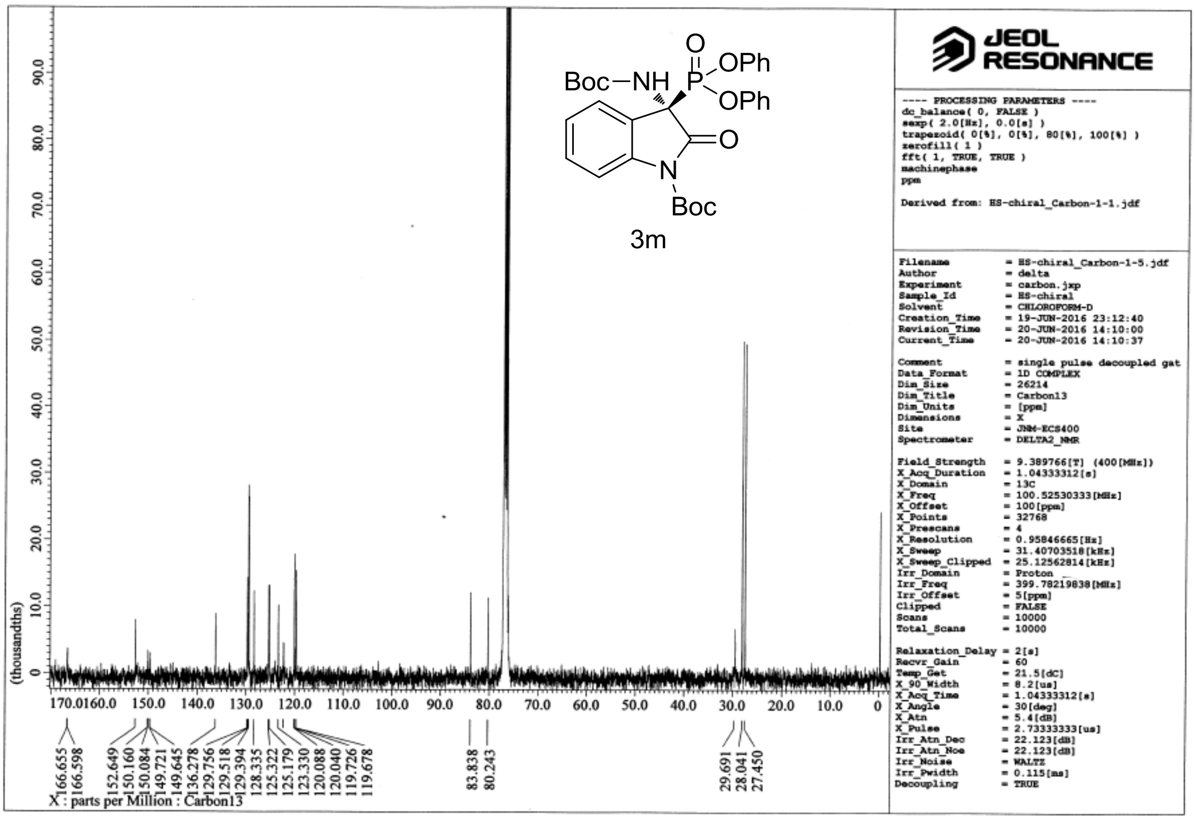

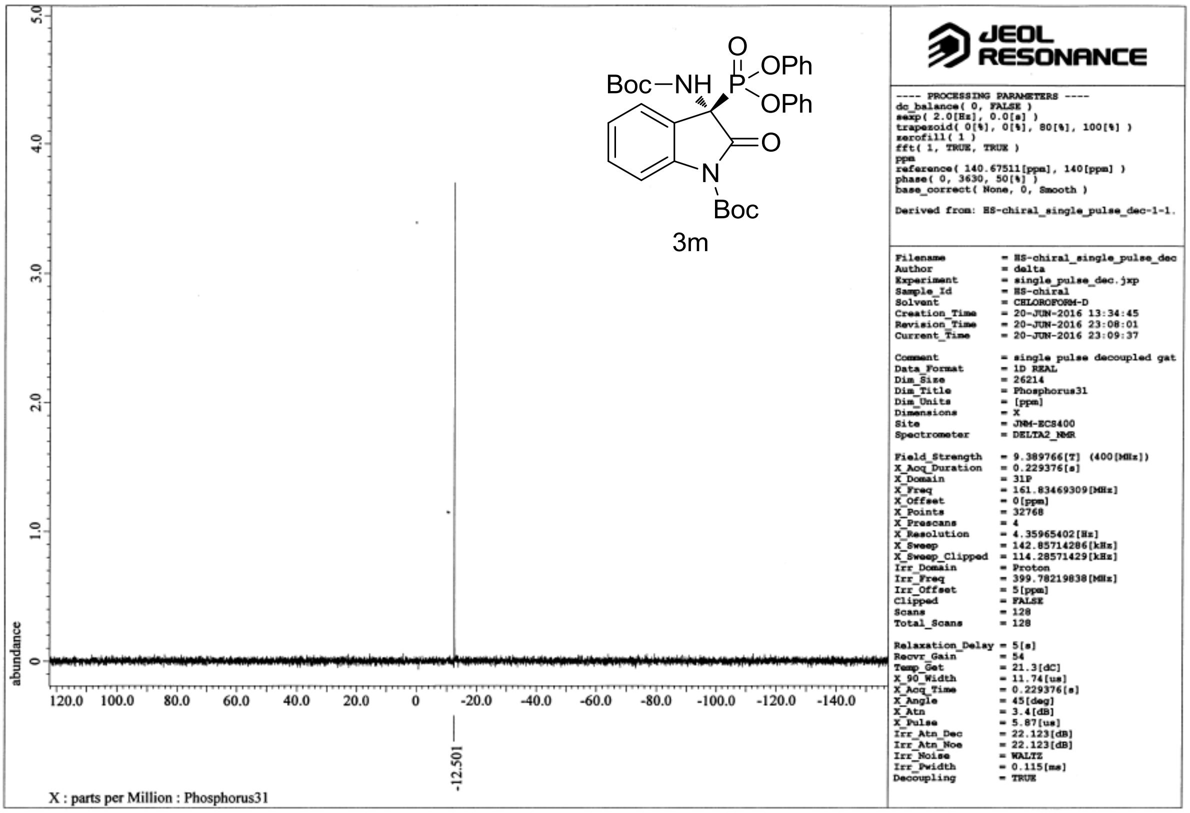
Boc—NH
O
P
OPh
OPh
O
N
Boc
3m
abundance
5.0
4.0
3.0
2.0
1.0
0
120.0 100.0 80.0 60.0 40.0 20.0 0 -20.0 -40.0 -60.0 -80.0 -100.0 -120.0 -140.0
-12.501
X : parts per Million : Phosphorus31
JEOL RESONANCE
---- PROCESSING PARAMETERS ----
dc_balance(0, FALSE)
sexp(2.0[Hz], 0.0[s])
trapezoid(0[%], 0[%], 80[%], 100[%])
zerofill(1)
fft(1, TRUE, TRUE)
ppm
reference(140.67511[ppm], 140[ppm])
phase(0, 3630, 50[%])
base_correct(None, 0, Smooth)
Derived from: HS-chiral_single_pulse_dec-1-1.
Filename = HS-chiral_single_pulse_dec
Author = delta
Experiment = single_pulse_dec.jxp
Sample_Id = HS-chiral
Solvent = CHLOROFORM-D
Creation_Time = 20-JUN-2016 13:34:45
Revision_Time = 20-JUN-2016 23:08:01
Current_Time = 20-JUN-2016 23:09:37
Comment = single pulse decoupled gat
Data_Format = 1D REAL
Dim_Size = 26214
Dim_Title = Phosphorus31
Dim_Units = [ppm]
Dimensions = X
Site = JNM-ECS400
Spectrometer = DELTA2_NMR
Field_Strength = 9.389766[T] (400[MHz])
X_Acq_Duration = 0.229376[s]
X_Domain = 31P
X_Freq = 161.83469309[MHz]
X_Offset = 0[ppm]
X_Points = 32768
X_Prescans = 4
X_Resolution = 4.35965402[Hz]
X_Sweep = 142.85714286[kHz]
X_Sweep_Clipped = 114.28571429[kHz]
Irr_Domain = Proton
Irr_Freq = 399.78219838[MHz]
Irr_Offset = 5[ppm]
Clipped = FALSE
Scans = 128
Total_Scans = 128
Relaxation_Delay = 5[s]
Recvr_Gain = 54
Temp_Get = 21.3[dC]
X_90_Width = 11.74[us]
X_Acq_Time = 0.229376[s]
X_Angle = 45[deg]
X_Atn = 3.4[dB]
X_Pulse = 5.87[us]
Irr_Atn_Dec = 22.123[dB]
Irr_Atn_Noe = 22.123[dB]
Irr_Noise = WALTZ
Irr_Pwidth = 0.115[ms]
Decoupling = TRUE

<Chromatogram>

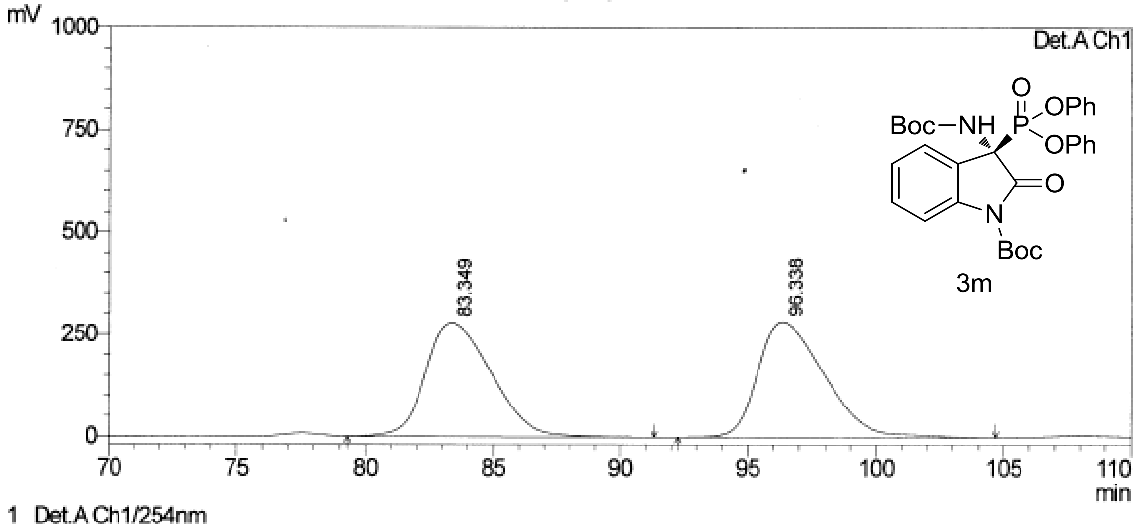

1 Det.A Ch1/254nm

PeakTable

Detector A Ch1 254nm

Peak#	Ret. Time	Area	Height	Area %	Height %
1	83.349	49728671	278460	49.625	49.665
2	96.338	50480778	282215	50.375	50.335
Total		100209449	560676	100.000	100.000

<Chromatogram>

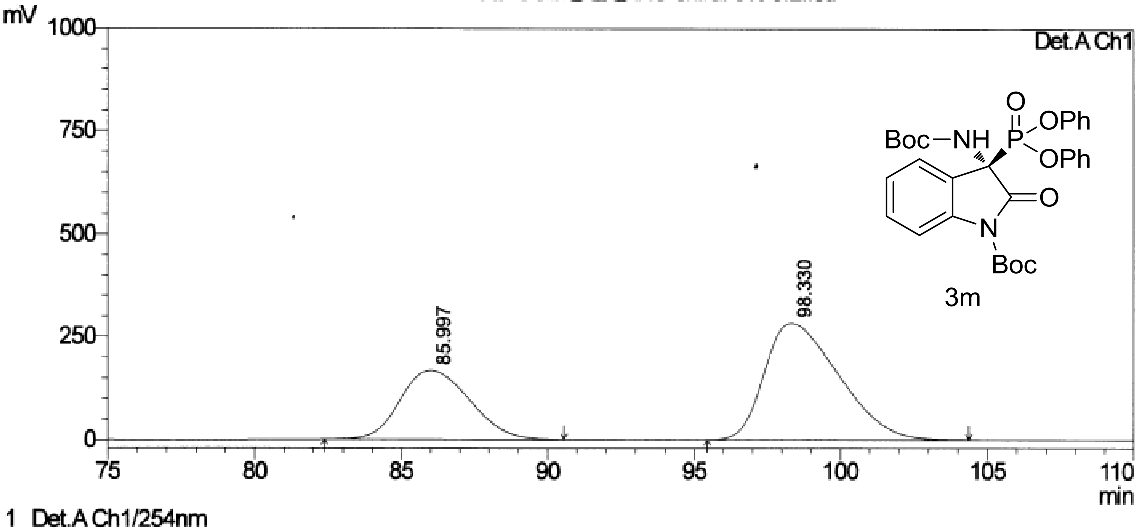

1 Det.A Ch1/254nm

PeakTable

Detector A Ch1 254nm

Peak#	Ret. Time	Area	Height	Area %	Height %
1	85.997	27756936	166772	35.720	37.080
2	98.330	49950735	282993	64.280	62.920
Total		77707671	449766	100.000	100.000
